# Metabologenomic Hallmark‐Based Discovery of Bacterial Thioamides as a New Lead against Drug‐Resistant Pancreatic Cancer

**DOI:** 10.1002/advs.202517849

**Published:** 2026-03-12

**Authors:** Young Eun Du, Eun Seo Bae, Thinh T. M. Bui, Seok Beom Lee, Dahan Kim, Jin‐Gyeong Park, Yongjoo Park, Soo Yeon Park, Sangwook Kang, Beomsu Lee, Daniel Shin, Yun Pyo Kang, In‐Gyun Lee, Dae‐Duk Kim, Seokhee Kim, Suckchang Hong, Kyuho Moon, Sang Kook Lee, Dong‐Chan Oh

**Affiliations:** ^1^ Natural Products Research Institute and Research Institute of Pharmaceutical Sciences, College of Pharmacy Seoul National University Seoul Republic of Korea; ^2^ College of Pharmacy Kyung Hee University Seoul Republic of Korea; ^3^ Research Institute of Pharmaceutical Sciences College of Pharmacy Seoul National University Seoul Republic of Korea; ^4^ Department of Chemistry Seoul National University Seoul Republic of Korea

**Keywords:** metabologenomic targeting, natural product, pancreatic cancer, stereochemistry, thioamide

## Abstract

Thioamides constitute an important class of pharmaceutically active natural products, yet their discovery and development are limited. A targeted metabologenomic method is developed to logically and efficiently discover thioamide compounds in bacteria. To this end, two strains were identified to possess genetic capacity for biosynthesizing thioamides from the bacterial genomic DNA library (1,192 strains) using the polymerase chain reaction to target the TfuA‐encoding gene, a genomic hallmark of thioamide biosynthesis. Mass spectrometric isotopic patterns of sulfur‐bearing compounds serve as metabolomic hallmarks to detect thioamide production from the extracts of the selected strains without chromatography. Applying this metabologenomic targeting approach, two new thioamides, thiogochangamides A and B, belonging to the thioviridamide family whose stereochemistry has remained unresolved for two decades, were discovered in *Streptomyces* sp. Their absolute configurations were fully assigned through chemical derivatizations, including the advanced Marfey method, Mosher method, partial hydrolysis, synthesis of an unusual amino acid, and desulfurization, combined with computational methods. Thiogochangamide B exhibits potent inhibitory activity against gemcitabine‐resistant pancreatic cancer cells both in vitro and in vivo. Mechanistically, thiogochangamide B effectively downregulates Wnt/β‐catenin signaling, thereby suppressing the metastatic potential of drug‐resistant cancer cells. This study provides a new therapeutic strategy for overcoming recalcitrant drug‐resistant pancreatic cancer.

## Graphical abstract

A targeted metabologenomic strategy integrating genomic and metabolomic hallmarks enabled the discovery of two new thioamides, thiogochangamides A and B, from bacteria. Their absolute configurations, previously unresolved within the thioviridamide family, were fully assigned through various chemical derivatizations and computational analysis. Thiogochangamide B displayed strong activity against gemcitabine‐resistant pancreatic cancer cells. This study highlights its potential as a therapeutic lead for drug‐resistant cancer.

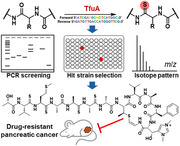

## 1 Introduction

Thioamides are an important class of organic molecules characterized by a unique structural feature: the thioamide group (─CSNH─), which is an isosteric replacement of the amide functionality (─CONH─) commonly found in many pharmaceutically active compounds [1]. Compounds containing thioamides exhibit promising bioactivities, including anticancer, anti‐neurodegenerative, anti‐inflammatory, analgesic, and anti‐hypertension/hyperglycemic effects, making this class of molecules valuable synthons in synthetic chemistry for drug development [2, 3, 4]. Reports of natural thioamides are rare, with cycasthioamides from the sago palm *Cycas revoluta* in 1997 representing the first instance [5]. Although the first natural thioamide was a phytochemical, thioamides are predominantly of bacterial origin.

Since the discovery in 2006 of the first bacterial thioamide, thioviridamide, which was isolated from the fermentation of *Streptomyces olivoviridis* and exhibited nanomolar cytotoxicity against the adenovirus E1A oncogene, thioviridamide‐like compounds have occasionally been identified through genome mining and heterologous expression, leading to the characterization of JBIR‐140, thioholgamides, thioalbamide, and TVA‐YJ‐4 [6, 7, 8, 9]. More recently, the structural diversity of thioholgamides has been expanded using a *Streptomyces*‐based system for thioholgamide library generation, enabling structure–activity relationship studies [10]. The continuous discovery of thioviridamide‐like compounds with potent anticancer activities has established this family as a major group of natural thioamides. Beyond thioviridamide‐like compounds, the structure of methanobactin has been reported in *Methylosinus trichosporium*, whereas saalfelduracin B was isolated from *Amycolatopsis saalfeldensis* as a ribosomally synthesized, post‐translationally modified peptide (RiPP) [11, 12]. In addition, a new polythioamide antibiotic, closthioamide, originating from a nonribosomal peptide synthetase pathway, was discovered in *Clostridium cellulolyticum*, further expanding the structural diversity of thioamides [13].

However, research on thioamide natural products currently faces three major limitations that hinder their discovery and development: (i) genome mining and heterologous expression are restricted to bacterial strains with fully sequenced genomes; (ii) the absolute configurations of thioamide compounds remain poorly characterized, as studies have relied primarily on X‐ray crystallography owing to the perceived lability of thioamide groups in degradation chemistry; and (iii) despite their promising in vitro activity, these compounds have rarely been evaluated in mammalian efficacy models and their mechanism of action have been under‐investigated, limiting their advancement toward biomedical applications.

In this work, we addressed these limitations through the following approaches: (i) a targeted search for bacterial thioamide natural products guided by metabologenomic hallmarks led to the discovery of two new thioamides, thiogochangamides A and B (compounds **1** and **2**, respectively); (ii) extensive chemical derivatizations, including desulfurization, partial hydrolysis, and the synthesis of unusual amino acids, combined with spectroscopic analysis and computational molecular modeling, enabled the assignment of the absolute configurations of the new compounds, representing the first stereochemical determination within the thioviridamide family; and (iii) evaluation of compound **2** in a mouse xenograft model, along with mechanistic studies, established it as a promising therapeutic lead against drug‐resistant pancreatic cancer for drug discovery and development.

## 2 Results and Discussion

**FIGURE 1:**
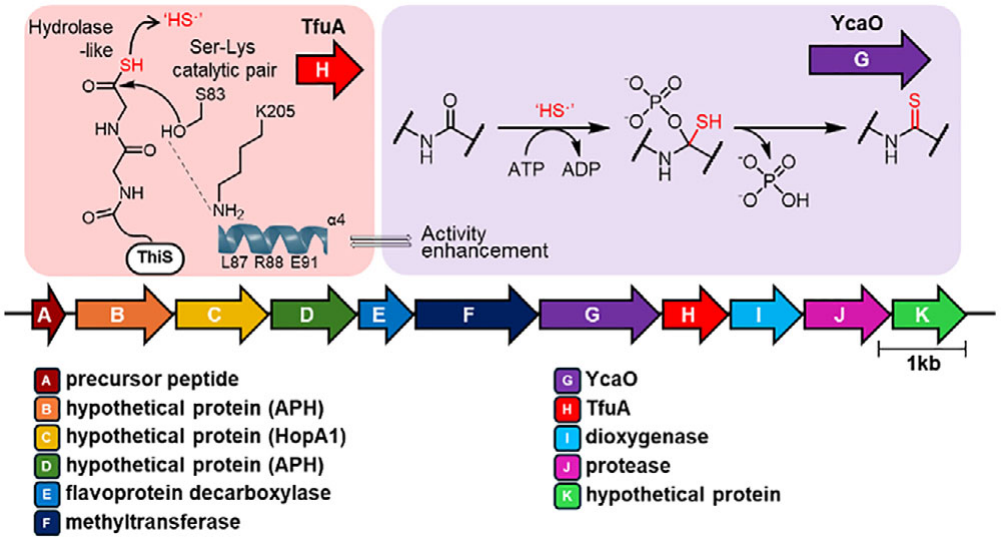
Representative biosynthetic pathway of thioamides within ribosomally synthesized and post‐translationally modified peptides.

TfuA and YcaO have been identified as essential enzymes in the major thioamide biosynthetic pathways (Figure 1) [14, 15, 16]. TfuA employs a Ser–Lys catalytic pair to hydrolyze ThiS‐COSH, generating a sulfide equivalent that is subsequently delivered to the YcaO active site. There, the sulfide acts as a nucleophile to form a thioamide through an *O*‐phosphorylated hemiorthoamide intermediate. Because these steps are unique to thioamide formation, the genes encoding these two enzymes can serve as genomic hallmarks for identifying bacterial strains capable of producing thioamide‐bearing peptides.

**FIGURE 2:**
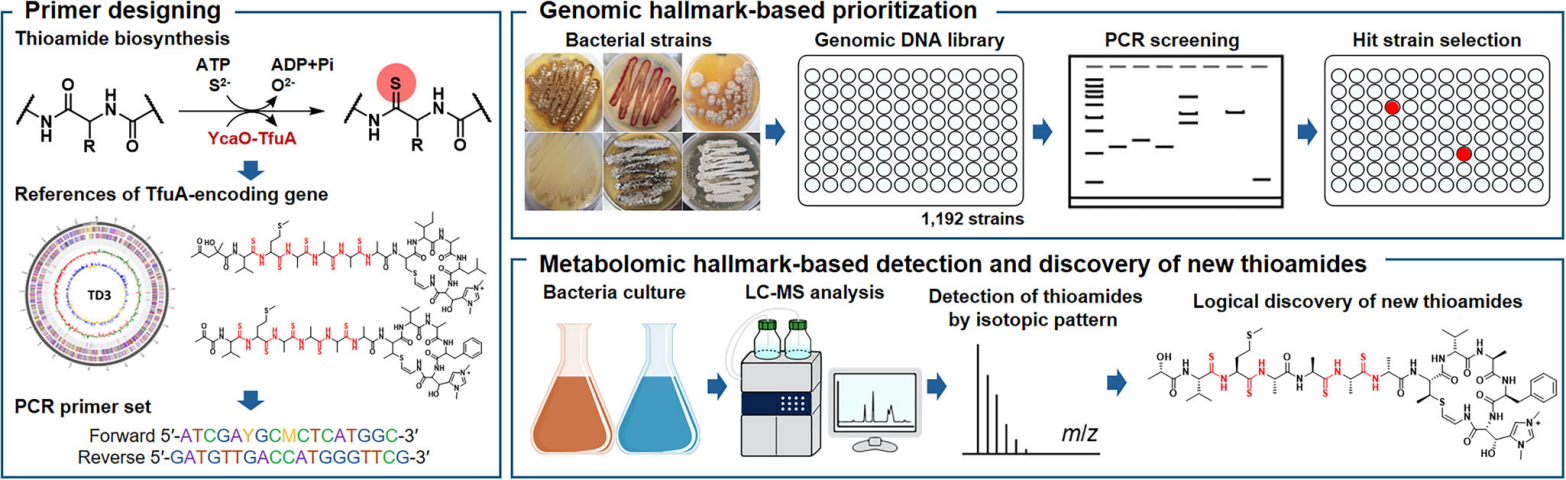
Schematic diagram of the discovery of new thioamides based on metabologenomic hallmarks.

However, because YcaO also catalyzes a biochemical reaction unrelated to thioamide biosynthesis [17], polymerase‐chain‐reaction (PCR) primer sets were designed to target the TfuA‐encoding gene, which serves as the genomic hallmark of thioamide biosynthesis. To design degenerate PCR primers, TfuA‐coding genes from five reference bacterial strains were used, along with our in‐house strain *Streptomyces* sp. TD3, which was isolated from Alaskan permafrost soil, possesses the TfuA gene in its complete genome (Table S12 and Figure S57). Mass spectrometry (MS) analysis of the culture extract suggested the production of thioamide‐like compounds. The major compound was purified and structurally elucidated by NMR, which confirmed its identity as thiostreptamide S4 (Figures S3–S9 and Table S10). Although this compound was previously reported in 2017 through a genome‐based approach using MS data [18], our study provides the first NMR assignment of thiostreptamide S4. These findings validate the use of strain TD3 as a reference for thioamide biosynthesis. Furthermore, optimization and validation of the primers with the TD3 strain identified an effective primer set, which amplified a 306‐bp fragment of the TfuA gene. PCR screening of our in‐house bacterial genomic DNA library comprising 1,192 strains, followed by amplicon sequencing, yielded two hits (*Streptomyces* sp. GC2 and DS4), corresponding to a hit rate of 0.17%. This low frequency indicates that thioamide biosynthetic pathways are rare in nature (Figure 2).

The hit strains were cultivated and subjected to chemical analysis to detect thioamides in the extracts using liquid chromatography (LC)–MS. In nature, sulfur has a characteristic isotopic ratio (^32^S:^34^S = 100:4.5), which generates the metabolomic hallmarks of sulfur‐bearing natural products. Because natural thioamide products contain one or more sulfur atoms in their structures, the production of thioamides in the hit strains selected based on the genomic hallmark essential for thioamide biosynthesis was monitored using this mass spectrometric metabolic hallmark (Figure 2).

LC‐HRMS chemical profiling of the GC2 and DS4 extracts unequivocally confirmed the production of sulfur‐bearing compounds. In particular, both GC2 and DS4 produced identical natural products with a molecular ion at *m*/*z* 1307.5093 (Figure S1a). High‐resolution mass spectrometric data revealed isotopic patterns characteristic of compounds containing multiple sulfur atoms. The molecular formula of the compound was predicted to be C_56_H_87_N_14_O_10_S_6_^+^, and the observed isotopic pattern of the ion was consistent with the simulation based on the molecular formula (Figure S1b). The detection of the metabolomic hallmarks of sulfur‐bearing compounds in the bacterial strains selected by genomic hallmarks prompted the scale‐up of bacterial cultures for the characterization of thioamide compounds. Since the two strains produced the same compounds, further chemical investigation focused on *Streptomyces* sp. GC2, which was isolated from the Gochang Ramsar site in the Republic of Korea, displayed higher yields based on LC‐HRMS analysis.

Thiogochangamide A (**1**) was obtained as a white powder after successive chromatographic purifications. The molecular formula C_56_H_87_N_14_O_9_S_6_^+^ was determined from high‐resolution electrospray ionization MS data (Figure S2) together with ^1^H and ^13^C NMR data in CD_3_CN (Figures S10 and S11). Comprehensive analysis of the ^1^H and HSQC spectra of **1** identified 12 amide/thioamide protons (δ_H_ 11.03, 10.60, 10.47, 8.87, 8.80, 8.25, 7.86, 7.84, 7.63, 7.60, 7.19, and 6.64) and all single‐bond ^1^H–^13^C correlations. The ^13^C NMR spectrum of **1** revealed four thioamide carbonyl carbons (δ_C_ 209.3, 206.8, 206.6, and 206.5) and eight amide carbons (δ_C_ 180.2, 176.4, 174.8, 174.1, 174.0, 172.9, 172.0, and 168.0), confirming the structural features of the thioamide‐bearing peptide.

Comprehensive analysis of the COSY, HMBC, and TOCSY NMR data (Figures S13–S16 and Table S11) revealed that **1** possessed one thiovaline, one thiomethionine, two thioalanines, one phenylalanine, one valine, and three alanines. Further examination of the 2D NMR spectra identified partial structures of lactic acid, *S*‐aminovinyl(‐methyl)‐cysteine (AviMeCys), and *N*,*N*‐dimethylhistidinium (dmHis). Through combined analysis of ^1^H–^15^N HSQC and HMBC spectra (Figures S17 and S18), the chemical shifts and positions of the nitrogen atoms in the structure were accurately assigned. In particular, the thioamide nitrogen atoms, resonating at δ_N_ 158.9–170.2, were clearly distinguished from ordinary amide nitrogens (δ_N_ 107.2–120.7).

**FIGURE 3:**
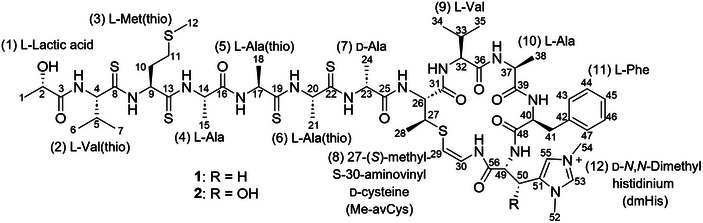
Structures of thiogochangamides A and B (compounds **1** and **2**, respectively).

The connections between the partial structures were elucidated based on two‐ or three‐bond ^1^H–^13^C couplings (Figure 3). HMBC correlations from NH‐4 (δ_H_ 7.60) to C‐3 (δ_C_ 180.2) connected lactic acid and valine in the second residue. *α*‐Amino H‐14 (δ_H_ 4.60) and H_2_‐10 (δ_H_ 2.24 and 2.57) to C‐13 (δ_C_ 206.6) showed the linkage of methionine to alanine in the fourth residue. Thus, other amino acid units can also be connected to each other. HMBC correlations from NH‐17 (δ_H_ 8.25) to C‐16 (δ_C_ 176.4), NH‐20 (δ_H_ 8.87) to C‐19 (δ_C_ 209.3), *α*‐amino H‐23 (δ_H_ 4.74) and H‐20 (δ_H_ 5.32) to C‐22 (δ_C_ 206.5), NH‐26 (δ_H_ 4.00) to C‐25 (δ_C_ 174.0), H‐26 (δ_H_ 4.00) and NH‐32 (δ_H_ 6.64) to C‐31 (δ_C_ 172.0), NH‐37 (δ_H_ 7.19) to C‐36 (δ_C_ 174.1), NH‐40 (δ_H_ 7.84) to C‐39 (δ_C_ 174.8), and NH‐49 (δ_H_ 7.63) to C‐48 (δ_C_ 172.9) determined the connections of the lactic acid–Val(thio)–Met(thio)–Ala–Ala(thio)–Ala(thio)–Ala–AviMeCys–Val–Ala–Phe–dmHis sequence, along with the ROESY correlation between H‐4 (δ_H_ 4.46) and NH‐9 (δ_H_ 10.60) of Val(thio) and Met(thio) (Figures S14 and S15). In addition, the macrocyclic ring was established based on the HMBC coupling of NH‐30 (δ_H_ 10.47) to C‐56 (δ_C_ 168.0). Compound **1** was identified as a new cyclic polythioamide containing lactic acid, AviMeCys, and dmHis, belonging to the thioviridamide family (Figure 3). This structure was further supported by the ^1^H–^15^N HMBC correlations and ^15^N chemical shifts (Figure S18 and Table S11).

Thiogochangamide B (**2**) was obtained as a powder together with **1** from *Streptomyces* sp. GC2. The molecular formula of **2** was established as C_56_H_87_N_14_O_10_S_6_^+^ by high‐resolution electrospray ionization MS (Figure S2), together with ^1^H and ^13^C spectra (Figures S19 and S20 and Table S11). The molecular formula of **2** indicated that it contained one more oxygen atom than **1**. As expected, **2** showed high similarity in its NMR spectrum to those of **1** (Figures S19–S25). Careful comparison of the 1D and 2D NMR data of **1** and **2** revealed that the β carbon of the dmHis moiety was hydroxylated. COSY correlations from NH‐49 (δ_H_ 7.61), H‐49 (δ_H_ 4.15), and H‐50 (δ_H_ 5.77), together with HMBC correlations from H‐50 (δ_H_ 5.77), H_3_‐52 (δ_H_ 3.91), H‐53 (δ_H_ 8.31), and H‐55 (δ_H_ 7.60) to C‐51 (δ_C_ 134.3), clarified the β‐hydroxy‐dmHis structure. Therefore, compound **2** was identified as a new member of the thioviridamide family (Figure 3).

**FIGURE 4:**
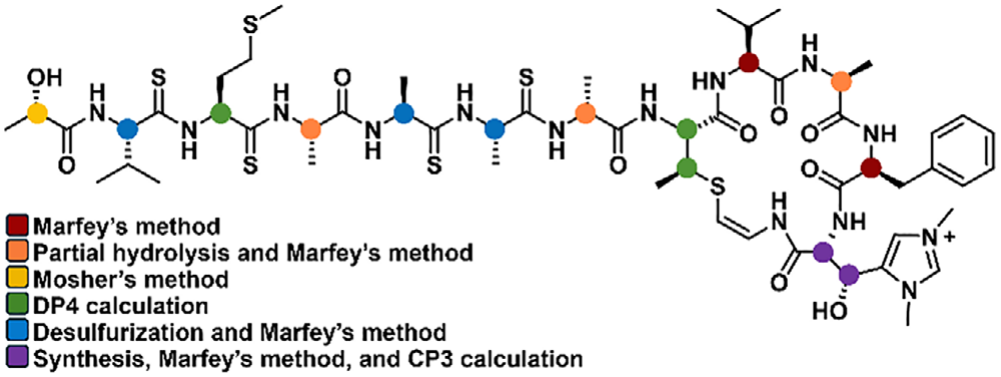
Strategy for the stereochemical analysis of **2**.

Although the first natural product of the thioviridamide family was reported nearly 20 years ago [7, 8], the absolute configurations of the compounds in this family have not been fully assigned, and some studies have assumed l‐configurations for all amino acid‐derived residues, sometimes arbitrarily [6]. This motivated us to investigate the stereochemistry of thiogochangamides A and B (**1**‐**2**,) applying multiple chemical reactions and computational methods (Figure 4).

**FIGURE 5:**
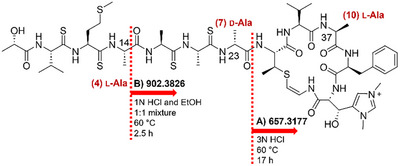
Partial hydrolysis and Marfey's analysis result for Ala in **2**.

The absolute configurations at the α‐carbons of the proteinogenic amino acid units in **1** and **2** were determined using the advanced Marfey method [19]. The acid hydrolysates of **1** and **2** were separately derivatized with the l‐ and d‐forms of 1‐fluoro‐2,4‐dinitrophenyl‐5‐alanine amide (FDAA). LC–MS analysis of the FDAA derivatives of the hydrolysates showed that both possessed l‐Val and l‐Phe (Table S1). Advanced Marfey analysis further revealed that thiogochangamides contain two l‐Ala and one d‐Ala residues, which complicates stereochemical assignment by necessitating partial hydrolysis (Table S1). Because **2** was the major product, further chemical reactions were conducted with it. By adjusting the ethanol concentration and acid conditions during the partial acid hydrolysis of **2**, a partial structure containing one Ala unit and another substructure containing two Ala units was obtained and purified by HPLC (Figure 5; Figure S47). Each hydrolysis product was analyzed by HR‐MS and Marfey's method after complete hydrolysis, which ultimately assigned the 14*S*, 23*R*, and 37*S* configurations. Therefore, the Ala unit at residue 7 was determined to be d‐Ala (Table S2).

**FIGURE 6:**
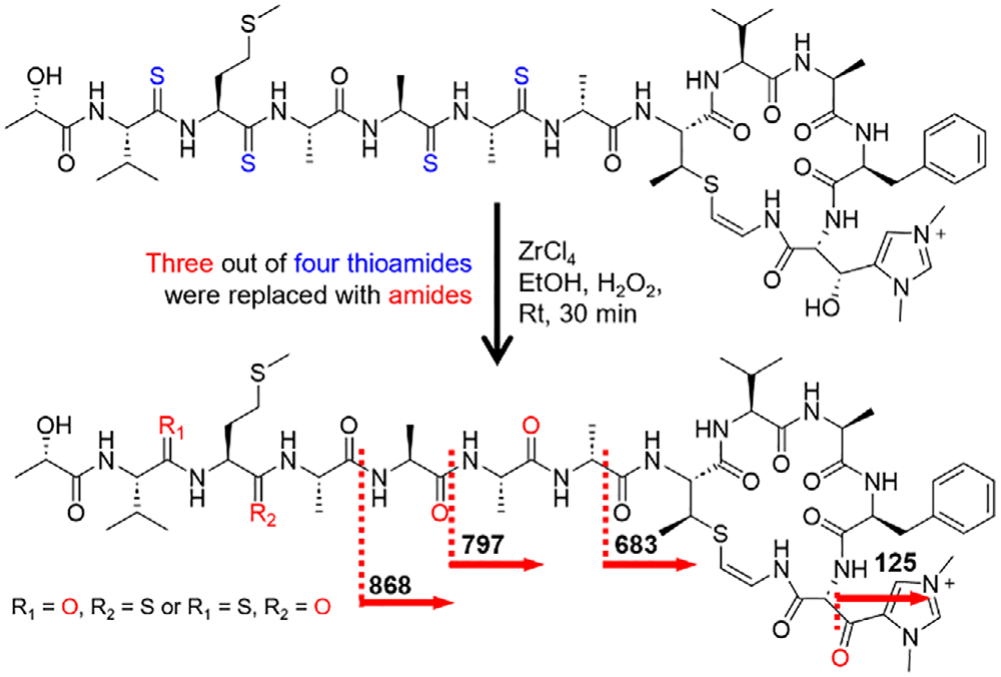
Amidation (desulfurization) of **2**.

The Val(thio), Met(thio), and two Ala(thio) units at residues 2, 3, 5, and 6 were degraded during acid hydrolysis. This problem was overcome by converting the thioamides into amides in **2** via amidation with ZrCl_4_ and H_2_O_2_ [20]. Extensive optimization of the desulfurization reaction and chromatography enabled the characterization of the major amidation products by HR‐MS/MS (Figure 6; Figure S48). Subsequent acid hydrolysis and Marfey derivatization increased the ratio of l‐ and d‐Ala–FDAA adducts from 2:1 to 4:1, allowing assignment of the l‐configuration to the two Ala(thio) units (Table S3). Marfey's analysis also showed an increase in the number of l‐Val–FDAA derivatives compared with that of the l‐Phe–FDAA adducts, indicating that the additional l‐Val–FDAA originated from Val(thio), thereby establishing its l‐configuration (Table S3). However, the desired Marfey derivatives from Met were not detected because Met(thio) was not converted to Met in the major amidated product, requiring further stereochemical analysis.

The C‐2 secondary alcohol‐bearing stereogenic center in lactic acid was determined using a modified Mosher's method [21]. The C‐2 hydroxy group in **2** was acylated with *R*‐(−)‐ and *S*‐(+)‐α‐methoxy‐α‐(trifluoromethyl)phenyl acetyl chloride (MTPA‐Cl) to furnish the corresponding *S*‐ and *R*‐MTPA esters, respectively (Figure S45). Analysis of ^1^H NMR and COSY spectra allowed the assignment of the proton chemical shifts for both esters. Calculation of the ∆δ*_S_*_‐_ *_R_* values enabled the assignment of the 2*S* configuration, thereby establishing l‐lactic acid (Figures S26–S31 and S46).

As mentioned above, the difference between **1** and **2** lies in the His‐derived unit. Compound **1** contains dmHis, whereas **2** contains β‐hydroxy‐dmHis. Subsequently, l‐ and d‐dmHis were synthesized and derivatized with l‐ and d‐FDAA, respectively (Figures S49 and S50). After the acid hydrolysis of **1** and FDAA derivatization, comparison of the elution order of the FDAA products of dmHis from **1** with the synthesized products established the d‐configuration (*R*) for this unit (Table S1). Based on the common biosynthetic origins of **1** and **2**, the C‐49 stereogenic center of β‐OH‐dmHis was also deduced to be *R*. *J*‐based configuration analysis, using ^1^H–^1^H and ^1^H–^13^C coupling constants acquired from a *J*‐resolved HMBC NMR experiment, assigned C‐50 as *R* (3*R*‐OH‐dmHis) (Figure S52). This assignment was further supported by the DFT modeling of the FDAA products of β‐OH‐dmHis and their elution order in advanced Marfey's analysis (Figure S54).

The stereochemically unassigned AviMeCys and Met(thio) residues were further analyzed using quantum‐mechanics‐based DP4 calculations [22]. Four possible truncated diastereomers of AviMeCys in **1**, namely **1a** (26*R* and 27*R*), **1b** (26*R* and 27*S*), **1c** (26*S* and 27*R*), and **1d** (26*S* and 27*S*), were constructed. Subsequently, ^1^H and ^13^C chemical shifts of the 71 conformers were calculated and averaged according to their Boltzmann populations. DP4 calculations, based on statistical comparison of the calculated and experimental chemical shifts, indicated that diastereomer **1b** (26*R* and 27*S*: *S*‐methyl‐d‐Cys) matched **1** with 100.0% probability (Figure S55 and Tables S6 and S7). Similarly, DP4 calculations assigned the absolute configuration of Met(thio) as l with 100.0% probability (Figure S49 and Tables S8 and S9).

Thiogochangamide A (**1**) was identified as a new thioamide‐bearing compound containing l‐lactic acid, l‐Val(thio), l‐Met(thio), l‐Ala, l‐Ala (thio), l‐Ala(thio), l‐Ala(thio), d‐Ala, aminovinyl‐3*S*‐methyl‐d‐Cys, l‐Val, l‐Ala, l‐Phe, and d‐dmHis. Thiogochangamide B (**2**) was found to incorporate 3*R*‐OH‐d‐dmHis while retaining the same stereochemistry as **1** at all other chiral centers (Figure 3).

The biosynthetic gene cluster (BGC) for thiogochangamides in *Streptomyces* sp. GC2 was identified using AntiSMASH and showed a high similarity to thioholgamide (Figure 2; Tables S13–S15) [23]. The putative RiPP‐derived biosynthetic pathway of **1** and **2** was also proposed based on those of the reported thioviridamide family compounds (Figure S58) [15, 24]. To date, four types of enzymes are known to mediate the production of d‐amino acid in ribosomally synthesized, post‐translationally modified peptides: radical SAM enzymes, thiazoline‐dependent epimerases, dehydroalanine reductases, and a pair of membrane‐associated epimerases [25, 26, 27, 28]. No enzymes in the thiogochangamide biosynthetic gene cluster contain domains homologous to known epimerases, suggesting that its d‐amino acids may be generated by novel mechanisms (Table S15), warranting further biosynthetic investigation. Furthermore, the hypothetical ThioK homologue in the thiogochangamide BGC showed no predicted catalytic activity (Table S15).

Many thioamide‐bearing compounds have been previously reported to exhibit cytotoxic activities [2]. Therefore, we evaluated the anticancer activity of the major metabolite. In the bioassays against a panel of human cancer cell lines, including A549 (lung), HCT116 (colon), SNU638 (gastric), SK‐HEP1 (liver), and MDA‐MB231 (breast) cells, compound **2** exhibited remarkable cytotoxicity (Table S16).

Pancreatic cancer is an aggressive malignancy with a poor prognosis, low early‐diagnosis rates, and limited treatment options. Owing to its anatomical location, asymptomatic progression, and rapid disease course, only 15%–20% of patients are eligible for surgery, and most experience recurrence [29, 30]. Mortality ranks among the leading causes of cancer‐related deaths, with a 5‐yr survival rate of only 10% [31]. Acquired gemcitabine resistance remains a major obstacle to effective treatment, underscoring the need to elucidate the underlying molecular mechanisms and develop small molecules capable of overcoming this resistance.

**FIGURE 7:**
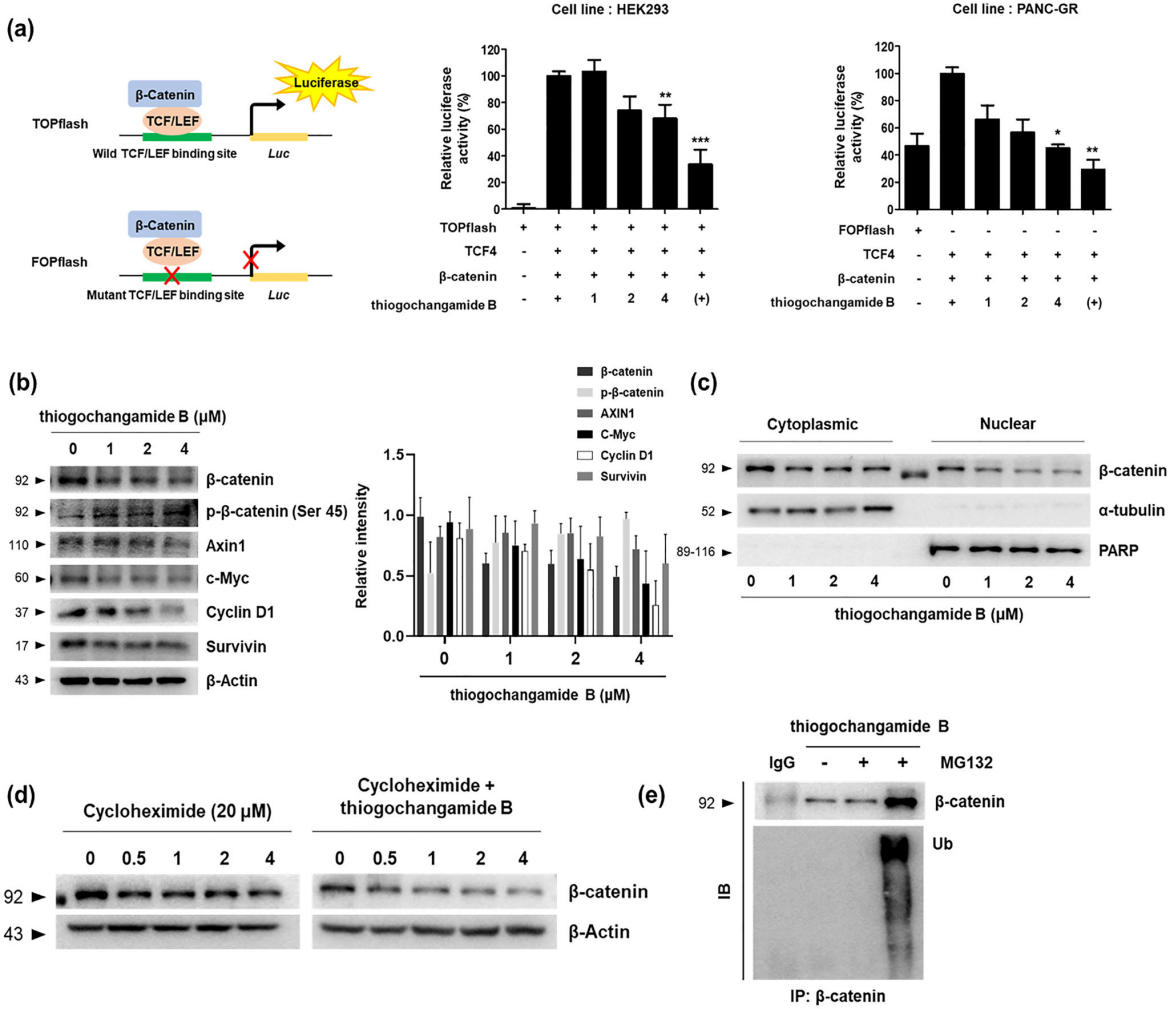
Thiogochangamide B (**2**) downregulates β‐catenin and its target genes via proteasomal degradation. (a) TCF/LEF reporter assay (TopFlash) showing decreased β‐catenin transcriptional activity upon treatment with **2** (1, 2, and 4 µm) for 24 h. (b) Western blot analysis of β‐catenin and Wnt target genes (c‐Myc, Cyclin D1, and Survivin) following 24‐h treatment with **2** at the indicated concentrations. β‐Actin was used as an internal control (left panel). Densitometric quantification of Wnt target protein levels was normalized to β‐actin (right panel). (c) Western blot analysis of β‐catenin in nuclear and cytoplasmic fractions after 24‐h treatment with **2** (1, 2, and 4 µm). PARP and α‐tubulin were used as nuclear and cytoplasmic markers, respectively. (d) Time‐course cycloheximide chase assay evaluating the β‐catenin protein stability in the presence or absence of **2** (4 µm). (e) β‐Catenin and ubiquitin protein levels in cells co‐treated with **2** (4 µm) and the proteasome inhibitor MG132 (10 µm) for 4 h. Data are presented as the means ± SD from three independent experiments.

Next, we evaluated thiogochangamide B (**2**) as a potential therapeutic agent for treating pancreatic cancer and overcoming acquired drug resistance to gemcitabine. Notably, **2** demonstrated potent cytotoxicity against parental and gemcitabine‐resistant pancreatic cancer cells, with IC_50_ values in the low micromolar range (Table 1; Figure S59). Aberrant activation of the Wnt/β‐catenin signaling pathway is known to contribute to chemoresistance and maintenance of cancer stem‐like properties in pancreatic cancer [32, 33]. Based on these insights, we hypothesized that **2** may exert its anticancer effects, at least in part, by interfering with the β‐catenin signaling activity. Indeed, β‐catenin expression was found to be elevated in gemcitabine‐resistant pancreatic cancer cell lines, and its clinical significance was analyzed using KM plots (Figure S60). To evaluate the effect of **2** on Wnt/β‐catenin signaling, we first conducted a TCF/LEF luciferase reporter assay using the TopFlash system. Treatment with **2** significantly reduced the luciferase activity in PANC‐GR cells, indicating inhibition of the β‐catenin‐mediated transcriptional activity (Figure 7a). To examine whether thiogochangamide B (**2**) functionally engages β‐catenin in a cellular context, a cellular thermal shift assay (CETSA) was conducted (Figure S61). CETSA evaluates ligand‐induced changes in protein thermal stability, as binding interactions can stabilize the folded state of a protein and shift its apparent aggregation temperature upon heat challenge. By comparing the soluble protein fraction across a temperature gradient in intact cells, CETSA allows direct assessment of compound‐target engagement under physiologically relevant conditions [34]. Given that β‐catenin functions within multiprotein complexes in cells, this approach provides a relevant means to evaluate its interaction with thiogochangamide B. Consistently, compound **2** induced thermal stabilization of β‐catenin, supporting cellular target engagement.

**TABLE 1:** Antiproliferative activity of **2** in human pancreatic cancer cells.

IC_50_ (µm)	PANC‐1^a^	PANC‐GR^a^
**2**	1.2 ± 0.17	2.9 ± 0.17
^b^Gemcitabine	2.1± 0.05	>50

^a^ Cancer cell lines: PANC‐1 (pancreatic) and PANC‐GR (gemcitabine‐resistant PANC‐1).

^b^ Gemcitabine was used as the positive control.

Next, we examined the protein levels of β‐catenin and downstream proteins of the Wnt target genes using western blotting. Treatment with **2** led to a concentration‐dependent decrease in the total β‐catenin levels, which was accompanied by an increase in phosphorylated β‐catenin (Ser45) and a marked reduction in c‐Myc, Cyclin D1, and Axin1 expression (Figure 7b). To further assess its transcriptional activity, we investigated the subcellular localization of β‐catenin following treatment with **2**. As shown in Figure 7c, the cytoplasmic and nuclear fractions were isolated and analyzed by western blotting. The findings demonstrated a decrease in the β‐catenin levels in the nuclear fraction, indicating that **2** suppresses the nuclear translocation of β‐catenin.

To further investigate the mechanism of β‐catenin downregulation, cells were treated with cycloheximide to inhibit new protein synthesis, and accelerated degradation of β‐catenin was observed upon **2** treatment (Figure 7d). To validate whether **2** affects β‐catenin degradation, we assessed β‐catenin levels in the presence of the proteasome inhibitor MG132. The reduction of β‐catenin by **2** was reversed upon MG132 co‐treatment, suggesting that **2** promotes β‐catenin degradation via the proteasome pathway (Figure 7e). Collectively, these data demonstrate that **2** effectively suppresses Wnt/β‐catenin signaling in PANC‐GR cells by downregulating β‐catenin expression and impairing its nuclear accumulation. Furthermore, β‐catenin depletion was shown to restore gemcitabine sensitivity, and treatment with **2** induced cell cycle arrest and apoptosis in resistant cells (Figures S62–S64).

Previous studies have reported that β‐catenin plays a critical role in maintaining cancer stem cell properties and promoting differentiation processes associated with metastasis [35]. Therefore, we hypothesized that the downregulation of β‐catenin expression could impair the metastatic characteristics of gemcitabine‐resistant pancreatic cancer cells. To validate this point, RNA interference was used to knock down β‐catenin expression in PANC‐GR cells, followed by performing migration and invasion assays. As shown in Figure S65, β‐catenin knockdown significantly decreased cell motility and invasiveness, accompanied by the altered expression of epithelial–mesenchymal transition (EMT) markers.

**FIGURE 8:**
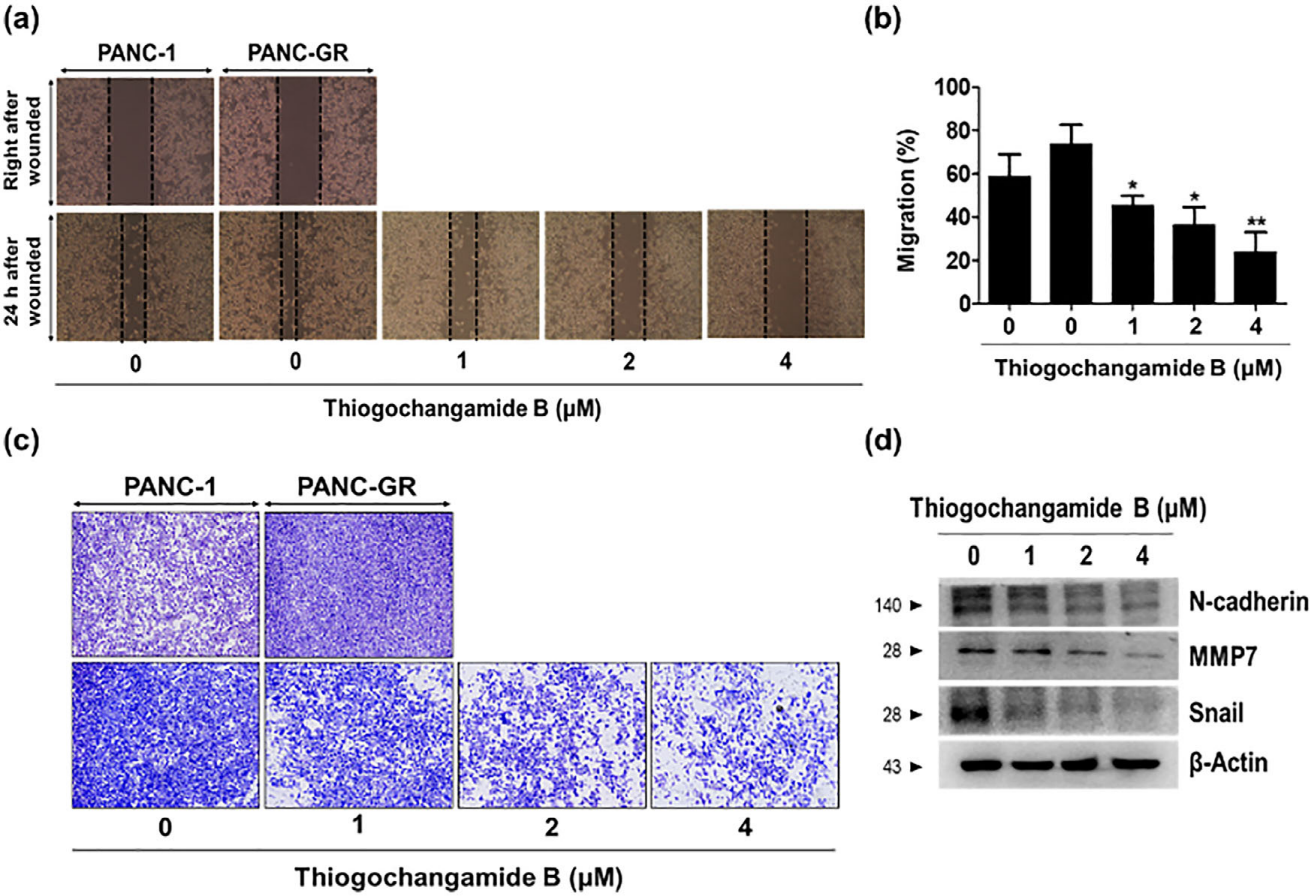
Thiogochangamide B (**2**) alters the migratory and invasive properties of PANC‐GR cells. (a,b) Wound‐healing assay of PANC‐GR cells treated with **2** (1, 2, and 4 µm) for 24 h and compared with those obtained using PANC‐1 cells. Representative images were taken at 0 and 24 h after scratching and quantified with ImageJ software. (c) Transwell invasion assays after the 24‐h treatment of **2**. Invaded cells were stained with a crystal‐violet solution. (d) Western blot analysis of epithelial–mesenchymal transition markers (N‐cadherin, MMP7, and Snail) in PANC‐GR cells treated with **2** (1 µm, 2 µm, and 4 µm) for 24 h. β‐Actin was used as a loading control. All experiments were performed in triplicate.

To determine whether **2** exerts similar antimetastatic effects, we first performed a wound‐healing assay. Treatment with **2** (1 µm, 2 µm, and 4 µm for 24 h) significantly inhibited wound closure in a concentration‐dependent manner compared with vehicle‐treated controls, indicating a reduced migratory capacity (Figure 8a,b). Next, we evaluated the effect of **2** on cell invasion using transwell assays. PANC‐GR cells treated with **2** displayed marked suppression of both migration and invasion through the membrane relative to those of the vehicle‐treated control group (Figure 8c). Quantitative analyses confirmed that cell migration and invasion were significantly inhibited in a concentration‐dependent manner. To elucidate the underlying molecular mechanism, we examined the expressions of key EMT‐related proteins by western blotting. Treatment with **2** resulted in the downregulation of mesenchymal markers, including *N*‐cadherin, MMP7, and Snail (Figure 8d). These data suggest that **2** suppresses EMT, thereby contributing to its antimigratory and anti‐invasive properties. Collectively, these findings indicate that **2** impairs the metastatic potential of gemcitabine‐resistant pancreatic cancer cells by inhibiting β‐catenin‐mediated signaling, ultimately suppressing cell migration, invasion, and EMT progression.

To validate the therapeutic efficacy and safety of **2** in vivo, we employed a cancer cell‐line‐derived xenograft model in Balb/c nude mice. PANC‐GR cells were subcutaneously inoculated into the right flank, and treatment was initiated once the tumor volume reached approximately 100 mm^3^. Mice received intraperitoneal administration of the vehicle (DMSO/cremophor/saline = 5:5:90), gemcitabine (20 mg/kg), or **2** (1 mg/kg), three times per week for 20 d [36]. A PANC‐1 cell‐implanted group was included for comparison of tumor‐tissue characteristics.

**FIGURE 9:**
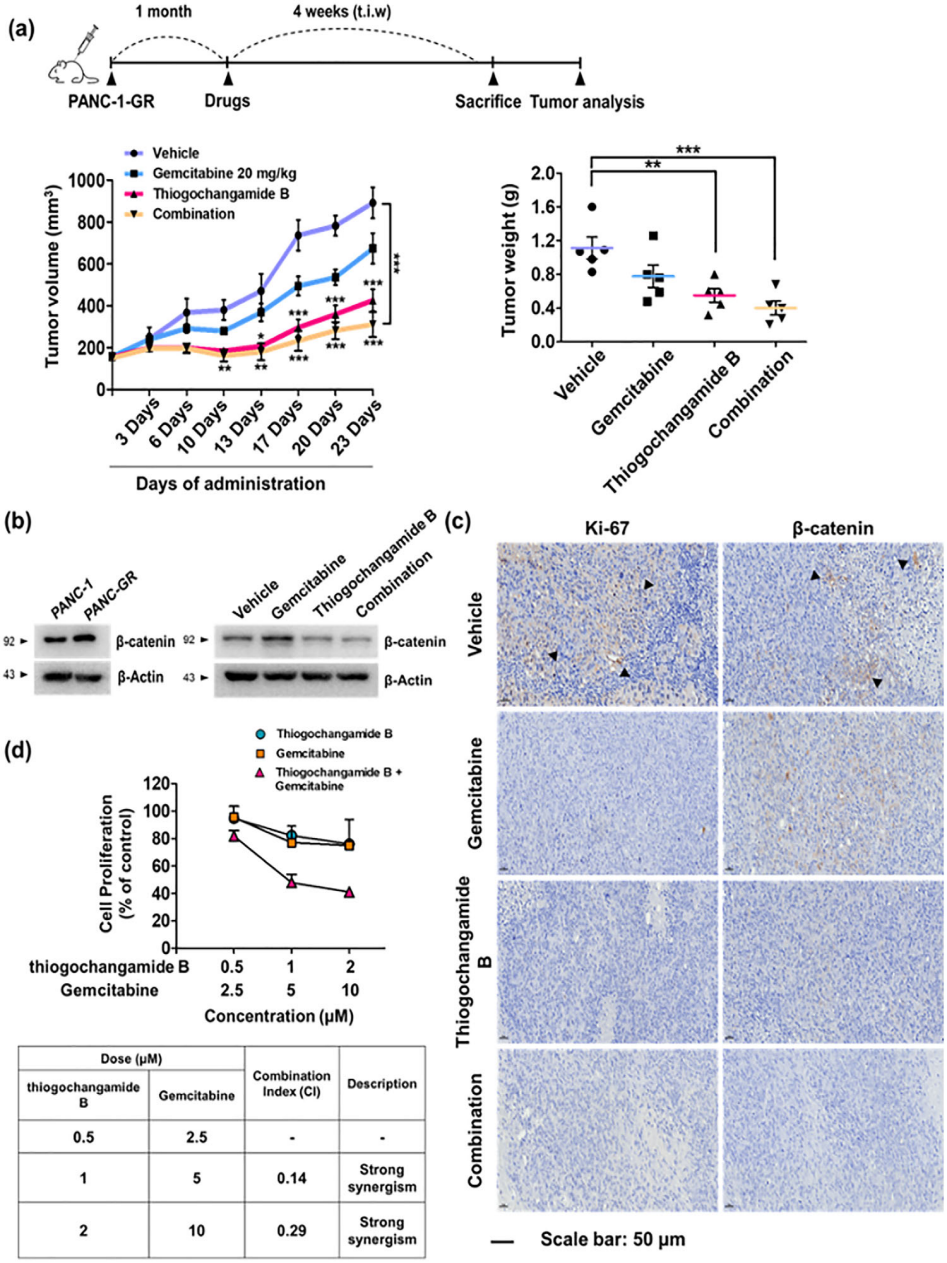
In vivo efficacy of **2** in a PANC‐GR cancer cell‐line‐derived xenograft (CDX) model. (a) Tumor volumes in PANC‐GR CDX mice (*n* = 5) treated with **2** and gemcitabine (left panel). Tumor weights were measured from excised tumors in each group at the end of the experiment (right panel). ^**^ *p* < 0.01 and ^***^ *p* < 0.001 indicate statistical significance compared with the vehicle‐treated control. (b) Western blot analysis of the β‐catenin protein expression in homogenized tumor tissues from each group. β‐Actin was used as an internal control. (c) Immunohistochemistry analysis of excised tumors from PANC‐GR CDX mice at the end of the experiment. (d) Antiproliferative activity of 2 and gemcitabine in combination in PANC‐GR cells. Cells were treated with either 2 alone or in combination with gemcitabine for 72 h. Cell proliferation was assessed by the SRB assay, and the combination effect was evaluated using the Chou–Talalay method with combination indexes.

Consistent with our in‐vitro findings, treatment with **2**, either alone or in combination with gemcitabine, notably reduced the tumor volume and weight compared with those of the vehicle‐treated control groups (Figure 9a). Importantly, no significant changes in body weight or overt signs of toxicity were observed throughout the treatment period, indicating a favorable safety profile (Figure S66). Biochemical analysis of excised tumor tissues revealed markedly elevated β‐catenin expression in gemcitabine‐resistant tumors derived from PANC‐GR cells compared with those from PANC‐1 cells (Figure 9b). Immunohistochemical analysis further confirmed that β‐catenin expression was significantly downregulated in tumors from mice treated with **2**, either alone or in combination with gemcitabine, compared with that in the vehicle‐treated control groups. In addition, the expression of the cell proliferation marker Ki‐67 was consistently downregulated in thiogochangamide B‐treated groups, further supporting its inhibitory effect on tumor growth (Figure 9c).

To further evaluate the combination effect, PANC‐GR cells were treated with varying concentrations of **2** and gemcitabine for 72 h. Cell viability was assessed using an SRB assay, and the combination index (CI) was calculated. The results showed synergistic antiproliferative effects (CI < 1) (Figure 9d), corroborating the in vivo findings and suggesting the restoration of the gemcitabine sensitivity in PANC‐GR cells (Figure S67). Collectively, the results from both the gemcitabine‐resistant cell line and cancer cell‐line‐derived xenograft model support **2** as a promising antitumor lead compound for overcoming chemoresistance and inhibiting pancreatic cancer progression, both in vitro and in vivo.

Although gemcitabine has long served as a first‐line chemotherapeutic agent for pancreatic cancer, the development of acquired resistance severely limits its long‐term efficacy. Therefore, the identification of novel agents capable of overcoming gemcitabine resistance remains an urgent and unmet clinical need in pancreatic cancer management. Moreover, despite its high potential as a novel drug target, no Wnt/β‐catenin‐targeted inhibitors have yet been approved for the treatment of this malignancy.

**FIGURE 10:**
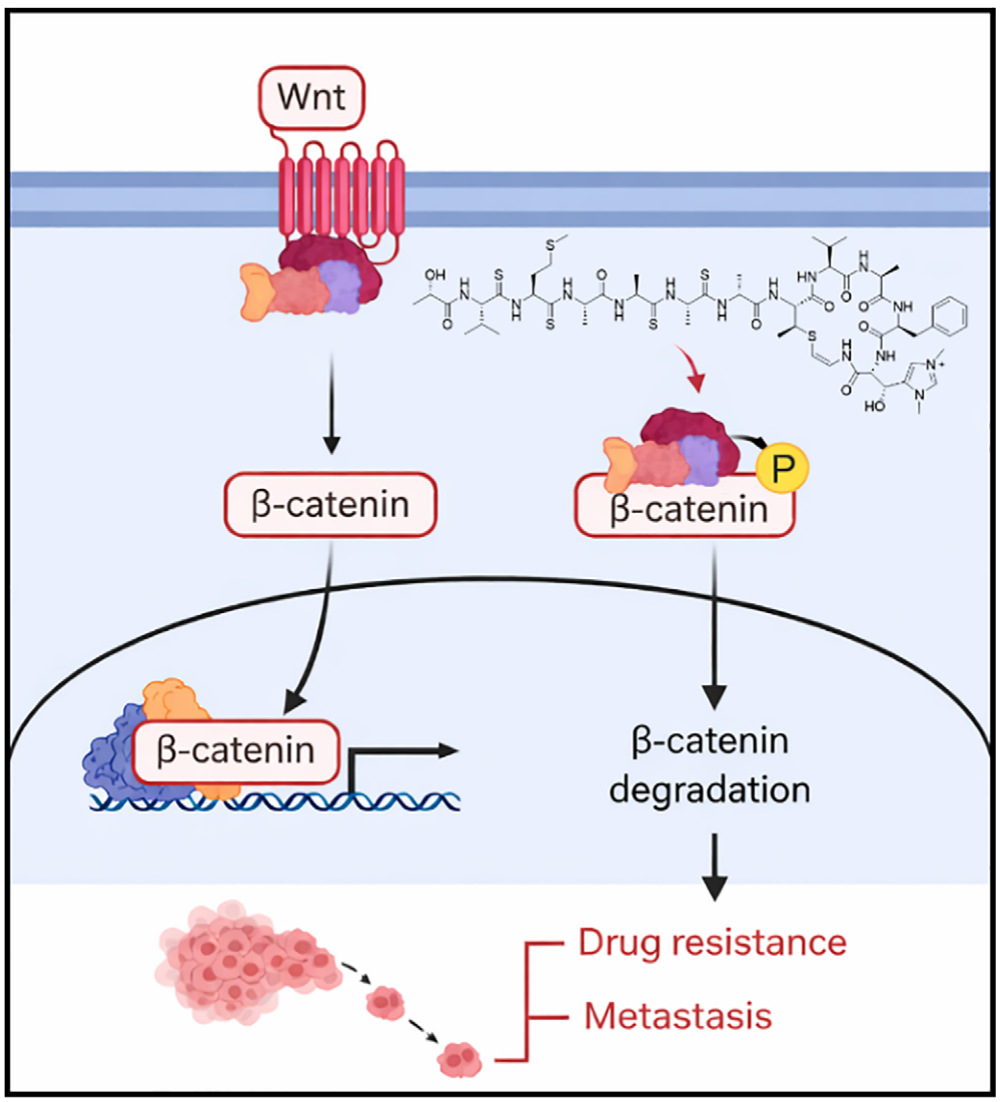
Summarized mechanism of action of thiogochangamide B (**2**) against pancreatic cancer.

Treatment with **2** led to a pronounced downregulation of β‐catenin expression and transcriptional activity, accompanied by significant antitumor effects. Notably, its ability to overcome gemcitabine resistance through inhibition of Wnt/β‐catenin signaling highlights a novel and promising therapeutic approach to address one of the most critical clinical challenges in pancreatic cancer (Figure 10).

To assess metabolic stability, thiogochangamide B was incubated with mouse liver S9 fractions and Balb/c mouse plasma. Minimal degradation was observed in the absence of NADPH, whereas time‐dependent metabolism occurred in its presence, indicating NADPH‐dependent enzymatic turnover (Figure S69). The intrinsic clearance (CL_int, in vitro_) was 4.96 µL/min/mg protein with a half‐life of 139.7 min. In plasma, the compound remained stable for at least 120 min, suggesting resistance to proteolytic degradation (see the Supporting Information for further details). These results indicate moderate metabolic stability and support further pharmacokinetic evaluation.

## 3 Conclusion

PCR‐based genomic screening of a bacterial genomic library (1,192 strains) using primers targeting the TfuA‐encoding gene identified two hit strains (0.17%), circumventing full genome analysis. Sequence analysis of the 306‐bp amplicons confirmed the presence of the TfuA‐encoding gene, the genomic hallmark of thioamide biosynthesis, selecting these strains as potential thioamide producers. LC‐HRMS analysis of crude extracts revealed the characteristic isotopic pattern of sulfur‐containing metabolites, based on the ^32^S:^34^S ratio of 100:4.5, serving as a metabolomic hallmark. This approach allowed the detection of new thioamide natural products, thiogochangamides A and B (**1** and **2**) from the *Streptomyces* hit strain isolated from a Ramsar site without the need for preparative chromatography, thereby accelerating their discovery.

Comprehensive spectroscopic analysis revealed the structures of **1** and **2** as new members of the thioviridamide family, whose stereochemistry had previously remained undetermined. The absolute configurations of the thiogochangamides were fully assigned for the first time in the thioviridamide family using a combination of the advanced Marfey's method, Mosher method, partial hydrolysis, desulfurization, synthesis of the unusual amino acid, and computational analyses. This approach provides a generalized strategy for the stereochemical analysis of thioamides. Unexpectedly, **1** and **2** were found to contain three d‐amino acid units.

Biological assays showed that **2** potently inhibited the growth of gemcitabine‐resistant pancreatic cancer cells both in vitro and in vivo. In addition, **2** displayed a synergistic effect with gemcitabine in suppressing the proliferation of pancreatic cancer cells. Mechanistic studies demonstrated that **2** downregulates the Wnt/β‐catenin signaling pathway and impairs the metastatic potential of pancreatic cancer cells. Importantly, **2** represents the first peptide molecule identified to inhibit Wnt/β‐catenin signaling while effectively targeting gemcitabine‐resistant pancreatic cancer cells. These results highlight the potential of **2** as a lead compound for overcoming drug resistance in pancreatic cancer, which is particularly malignant and has a very low survival rate (∼10%). In addition, our findings show that a natural product can provide a new chemotype that concurrently inhibits Wnt/β‐catenin signaling and suppresses drug‐resistant cancer cells, representing a significant advancement in the field. Notably, thiogochangamide B exhibited moderate metabolic turnover and substantial plasma stability, suggesting a favorable stability profile for a peptide‐derived natural product. Combined with its in vivo efficacy and mechanism‐based activity, these properties enhance its potential as a drug lead for further development against drug‐resistant pancreatic cancer.

The integrated targeted metabolomic approach developed in this study combines the genomic hallmarks of key biosynthetic enzyme‐coding genes with the HR‐MS‐based detection of sulfur‐containing metabolites as metabolomic hallmarks. Given that bacterial strains possessing the TfuA gene are very rare, with a hit rate of 0.17%, this method proved highly effective in discovering new thioamide compounds from approximately 1,200 bacterial strains lacking genomic data. It circumvents the need for whole‐genome sequencing in genome‐based discovery, chromatographic purification during the initial identification of thioamide metabolites from bacterial extracts, and individual chemical analysis of numerous (∼1,200) bacterial strains. By focusing on the structural motifs of pharmacological interest, this method enables the rapid prioritization of strains and streamlines logical natural product discovery. Owing to its broad applicability, this strategy has strong potential as a universal and rational platform for uncovering structurally and functionally novel bioactive molecules from diverse microorganisms.

## Conflicts of Interest

The authors submitted patent applications for thiogochangamide B and its biological activity.

## Supporting information




**Supporting File 1**: advs74755‐sup‐0001‐SuppMat.pdf.

## Data Availability

The data that support the findings of this study are available from the corresponding author upon reasonable request.
