# Supplementary material for: Metabologenomic Hallmark‐Based Discovery of Bacterial Thioamides as a New Lead against Drug‐Resistant Pancreatic Cancer
Source: Adv Sci (Weinh). 2026 Mar 12;13(28):e17849. doi: 10.1002/advs.202517849 (PMC13185837; doi:10.1002/advs.202517849)
Supplement: Supplementary file 1 — Supporting File 1: advs74755‐sup‐0001‐SuppMat.pdf. [file ADVS-13-e17849-s001.pdf]

SUPPORTING INFORMATION

Metabologenomic Hallmark-Based Discovery of Bacterial Thioamides as a New Leads against Drug-Resistant Pancreatic Cancer

Young Eun Du,+ Eun Seo Bae,+ Thinh T. M. Bui, Seok Beom Lee, Dahan Kim, Jin-Gyeong Park, Yongjoo Park, Soo Yeon Park, Sangwook Kang, Beomsu Lee, Daniel Shin, Yun Pyo Kang, In-Gyun Lee, Dae-Duk Kim, Seokhee Kim, Suckchang Hong, Kyuho Moon, Sang Kook Lee,* and Dong-Chan Oh*

Abstract: Thioamides constitute an important class of pharmaceutically active natural products, yet their discovery and development are limited. A targeted metabologenomic method is developed to logically and efficiently discover thioamide compounds in bacteria. To this end, two strains were identified to possess genetic capacity biosynthesizing thioamides from the bacterial genomic DNA library (1,192 strains) using the polymerase chain reaction to target TfuA-encoding gene, a genomic hallmark of thioamide biosynthesis. Mass spectrometric isotopic patterns of sulfur-bearing compounds serve as metabolomic hallmarks to detect thioamide production from the extracts of the selected strains without chromatography. Applying this metabologenomic targeting approach, two new thioamides, thiogochangamides A and B belonging to the thioviridamide family whose stereochemistry has remained unresolved for two decades, were discovered in *Streptomyces* sp. Their absolute configurations were fully assigned through chemical derivatizations, including the advanced Marfey method, Mosher method, partial hydrolysis, synthesis of an unusual amino acid, and desulfurization, combined with computational methods. Thiogochangamide B exhibits potent inhibitory activity against gemcitabine-resistant pancreatic cancer cells both *in vitro* and *in vivo*. Mechanistically, thiogochangamide B effectively downregulates Wnt/β-catenin signaling, thereby suppressing the metastatic potential of drug-resistant cancer cells. This study provides a new therapeutic strategy for overcoming recalcitrant drug-resistant pancreatic cancer.

+These authors contributed equally to this work.

*Corresponding authors: sklee61@snu.ac.kr and dongchanoh@snu.ac.kr

Table of Contents

Methods. **S4**
General experimental procedures. S4
Bacterial isolation, cultivation and extraction S4
Purification of oxidized-thiostreptamide S4 .... S4
Purification of thiogochangamides S4
Acid hydrolysis and FDAA derivatization of thiogochangamides A and B S5
Partial hydrolysis of thiogochangamide B S5
Mosher's method of thiogochangamide B S5
Amidation (desulfurization) of thiogochangamide B S5
Chemical synthesis of 4-(2-amino-2-carboxyethyl)-1,3-dimethyl-1H-imidazol-3-ium S6
Conformational search DP4 and CP3 calculation analysis S6
PCR primer designing and rapid PCR screening with the gDNA library S6
Whole genome sequencing and genomic analysis. S6
Cell culture conditions. S6
Drugs and chemical compounds. S6
Growth inhibition assay. S7
Bioinformatics Analysis. S7
Western blot analysis. S7
Transfection and Luciferase Reporter Gene Assay. S7
Cellular Thermal Shift Assay. S7
RNA Interference. S7
Wound healing migration assay. S7
Transwell cell invasion assay. S7
Synchronization and cell cycle analysis. S8
Annexin V-FITC/Propidium Iodide (PI) Double Staining Analysis. S8
In vivo drug efficacy assessment in mice. S8
Statistical Analysis. S8
Isothermal Titration Calorimetry (ITC). S8
In vitro metabolic stability in liver S9 fraction S8

MS spectra **S9**
Figure S1. Mass spectrum of a sulfur-bearing compound detected in the extract of *Streptomyces* sp. GC2 .... S9
Figure S2. HR-MS spectra of thiogochangamides A (**1**) and B (**2**) S10

NMR spectra. **S11**
Figure S3. ^{1}H NMR spectrum of oxidized-thiostreptamide S4 at 900 MHz in DMSO-d_6 .... S11
Figure S4. ^{13}C NMR spectrum of oxidized-thiostreptamide S4 at 225 MHz in DMSO-d_6 .... S11
Figure S5. HSQC NMR spectrum of oxidized-thiostreptamide S4 at 900 MHz in DMSO-d_6 .... S12
Figure S6. COSY NMR spectrum of oxidized-thiostreptamide S4 at 900 MHz in DMSO-d_6 .... S12
Figure S7. HMBC NMR spectrum of oxidized-thiostreptamide S4 at 900 MHz in DMSO-d_6 .... S13
Figure S8. TOCSY NMR spectrum of oxidized-thiostreptamide S4 at 900 MHz in DMSO-d_6 .... S13
Figure S9. ROESY NMR spectrum of oxidized-thiostreptamide S4 at 900 MHz in DMSO-d_6 .... S14
Figure S10. ^{1}H NMR spectrum of thiogochangamide A (**1**) at 800 MHz in acetonitrile-d_3. .... S15
Figure S11. ^{13}C NMR spectrum of thiogochangamide A (**1**) at 200 MHz in acetonitrile-d_3 .... S15
Figure S12. HSQC NMR spectrum of thiogochangamide A (**1**) at 800 MHz in acetonitrile-d_3 .... S16
Figure S13. COSY NMR spectrum of thiogochangamide A (**1**) at 800 MHz in acetonitrile-d_3 .... S16
Figure S14. HMBC NMR spectrum of thiogochangamide A (**1**) at 800 MHz in acetonitrile-d_3 .... S17
Figure S15. ROESY NMR spectrum of thiogochangamide A (**1**) at 800 MHz in acetonitrile-d_3 .... S17
Figure S16. TOCSY NMR spectrum of thiogochangamide A (**1**) at 800 MHz in acetonitrile-d_3 .... S18

Figure S17. ^{1}H-^{15}N HSQC NMR spectrum of thiogochangamide A (**1**) at 850 MHz in acetonitrile-d_3 .... S18
Figure S18. ^{1}H-^{15}N HMBC NMR spectrum of thiogochangamide A (**1**) at 850 MHz in acetonitrile-d_3 .... S19
Figure S19. ^{1}H NMR spectrum of thiogochangamide B (**2**) at 800 MHz in acetonitrile-d_3. .... S19
Figure S20. ^{13}C NMR spectrum of thiogochangamide B (**2**) at 200 MHz in acetonitrile-d_3 .... S20
Figure S21. HSQC NMR spectrum of thiogochangamide B (**2**) at 800 MHz in acetonitrile-d_3 .... S20
Figure S22. COSY NMR spectrum of thiogochangamide B (**2**) at 800 MHz in acetonitrile-d_3 .... S21
Figure S23. HMBC NMR spectrum of thiogochangamide B (**2**) at 800 MHz in acetonitrile-d_3 .... S21
Figure S24. ROESY NMR spectrum of thiogochangamide B (**2**) at 800 MHz in acetonitrile-d_3 .... S22
Figure S25. TOCSY NMR spectrum of thiogochangamide B (**2**) at 800 MHz in acetonitrile-d_3 .... S22
Figure S26. ^{1}H NMR spectrum of *S*-MTPA ester of thiogochangamide B (**6**) at 800 MHz in acetonitrile-d_3 .... S23
Figure S27. HSQC NMR spectrum of *S*-MTPA ester of thiogochangamide B (**6**) at 800 MHz in acetonitrile-d_3 .... S23
Figure S28. COSY NMR spectrum of *S*-MTPA ester of thiogochangamide B (**6**) at 800 MHz in acetonitrile-d_3 .... S24
Figure S29. ^{1}H NMR spectrum of *R*-MTPA ester of thiogochangamide B (**7**) at 800 MHz in acetonitrile-d_3 .... S24
Figure S30. HSQC spectrum of *R*-MTPA ester of thiogochangamide B (**7**) at 800 MHz in acetonitrile-d_3 .... S25
Figure S31. COSY spectrum of *R*-MTPA ester of thiogochangamide B (**7**) at 800 MHz in acetonitrile-d_3 .... S25
Figure S32. ^{1}H NMR spectrum of 4-(2-amino-2-carboxyethyl)-1,3-dimethyl-1H-imidazol-3-ium (**3**) at 400MHz in acetonitrile-d_3 .... S26
Figure S33. ^{1}H NMR spectrum of *N*,*N*-dimethylhistidinium D-FDAA-1 (**4**) at 850 MHz in DMSO-d_6 .... S26
Figure S34. ^{13}C NMR spectrum of *N*,*N*-dimethylhistidinium D-FDAA-1 (**4**) at 215 MHz in DMSO-d_6 .... S27
Figure S35. HSQC NMR spectrum of *N*,*N*-dimethylhistidinium D-FDAA-1 (**4**) at 850 MHz in DMSO-d_6 .... S27
Figure S36. COSY NMR spectrum of *N*,*N*-dimethylhistidinium D-FDAA-1 (**4**) at 850 MHz in DMSO-d_6 .... S28
Figure S37. HMBC NMR spectrum of *N*,*N*-dimethylhistidinium D-FDAA-1 (**4**) at 850 MHz in DMSO-d_6 .... S28
Figure S38. ROESY NMR spectrum of *N*,*N*-dimethylhistidinium D-FDAA-1 (**4**) at 850 MHz in DMSO-d_6 .... S29
Figure S39. ^{1}H NMR spectrum of *N*,*N*-dimethylhistidinium D-FDAA-2 (**5**) at 800 MHz in DMSO-d_6 .... S29
Figure S40. ^{13}C NMR spectrum of *N*,*N*-dimethylhistidinium D-FDAA-2 (**5**) at 200 MHz in DMSO-d_6 .... S30
Figure S41. HSQC NMR spectrum of *N*,*N*-dimethylhistidinium D-FDAA-2 (**5**) at 800 MHz in DMSO-d_6 .... S30
Figure S42. COSY NMR spectrum of *N*,*N*-dimethylhistidinium D-FDAA-2 (**5**) at 800 MHz in DMSO-d_6 .... S31
Figure S43. HMBC NMR spectrum of *N*,*N*-dimethylhistidinium D-FDAA-2 (**5**) at 800 MHz in DMSO-d_6 .... S31
Figure S44. ROESY NMR spectrum of *N*,*N*-dimethylhistidinium D-FDAA-2 (**5**) at 800 MHz in DMSO-d_6 .... S32

Determination of stereochemistry... S33
Figure S45. HR-MS data of thiogochangamide B *S* MTPA ester (**6**) and *R* MTPA ester (**7**) S33
Figure S46. $\Delta\delta_{S\text{-}R}$ values of (*S*)- and (*R*)-MTPA esters of thiogochangamide B (**2**) S34
Table S1. LC/MS analysis of D- and L-FDAA derivatives of the amino acid-derived units in thiogochangamide A (**1**) and B (**2**) S35
Figure S47. Partial hydrolysis of thiogochangamide B (**2**) and HR-MS data. S36
Table S2. LC/MS analysis of L-FDAA derivatives of hydrolyzed thiogochangamide B S37
Figure S48. HR-MS data of amidized in thiogochangamide B and HR-MS/MS data with a possible structure...... S38
Table S3. LC/MS analysis of D-FDAA derivatives of amidized thiogochangamide B... S39
Figure S49. Scheme for 4-(2-amino-2-carboxyethyl)-1,3-dimethyl-1 H-imidazol-3-ium (**3**) synthesis S40
Figure S50. LC/MS chromatogram of D- and L-FDAA derivatives of the synthetic *N*, *N*-dimethylhistidinium-derived units S41
Table S4. The major conformers of (D-*N*,*N*-dimethylhistidinium + D FDAA) and (L-*N*,*N*-dimethylhistidinium + D FDAA) identified by conformational searches in MMFF94 force field using MacroModel. S42
Table S5. Experimental (Exp.) chemical shift values of **4** and **5** with calculated (Cal.) chemical shift values (CS, *δ*) of (D-*N*,*N*-dimethylhistidinium + D FDAA) and (L-*N*,*N*-dimethylhistidinium + D FDAA). S43
Figure S51. CP3 calculation result of *N*, *N*-dimethylhistidinium D-FDAA derivatives. S44
Figure S52. *J*-resolved HMBC spectrum of thiogochangamide B (**1**) at 800 MHz in acetonitrile-d_3 and determination absolute stereochemistry of *β*-hydroxyl-*N*, *N*-dimethylhistidinium... S45
Figure S53. ROESY data analysis of thiogochangamide B (**2**) and determination absolute stereochemistry of *β*-hydroxyl-*N*, *N*-dimethylhistidinium... .... S46
Figure S54. DFT modeling results of D-*N*, *N*-dimethylhistidinium and *β*-(*R*)-hydroxyl- D-*N*, *N*-dimethylhistidinium S47

Figure S55. The simulated models of four possible diastereomers (**a**–**d**) of thiogochangamide A (**1**) and the result of DP4 calculation.......... S48
Table S6. The major conformers of cyclic partial structure identified by conformational searches in MMFF94 force field using MacroModel.......... S49
Table S7. Experimental (Exp.) and calculated (Cal.) chemical shift values (CS, δ) of cyclic diastereomers.......... S52
Figure S56. The simulated models of two possible diastereomers (**e** and **f**) of thiogochangamide B (**2**) and the result of DP4 calculation S54
Table S8. The major conformers of linear partial structure identified by conformational searches in MMFF94 force field using MacroModel.......... S55
Table S9. Experimental (Exp.) and calculated (Cal.) chemical shift values (CS, δ) of linear diastereomers.......... S56
Table S10. ^{1}H NMR and ^{13}C NMR spectral data NMR of oxidized-thiostreptamide S4 in DMSO-d_6.......... S58
Table S11. ^{1}H NMR and ^{13}C NMR spectral data NMR of thiogochangamides A (**1**) and B (**2**) in Acetonitrile-d_3 ....S59

Biosynthetic gene cluster (BGC) analysis.......... S61
Table S12. Primers used in this study. S61
Figure S57. Conserved region for TfuA enzyme PCR primer designing. S62
Table S13. antiSMASH analysis result of *Streptomyces* sp. GC2 genome S63
Table S14. antiSMASH analysis result of *Streptomyces* sp. TD3 genome S64
Table S15. Thiogochangamide biosynthetic gene cluster from *Streptomyces* sp. GC2.......... S65
Figure S58. Proposed biosynthetic pathways of thiogochangamide A (**1**) and B (**2**) S66

Additional bioassays.......... S67
Table S16. Anti-proliferative activity of thiogochangamide A and B in a panel of human cancer cell lines.......... S67
Figure S59. Sulforhodamine B (SRB) staining-based cytotoxicity assay in pancreatic cancer cells. S68
Figure S60. Correlation between CTNNB1 expression and overall survival in pancreatic adenocarcinoma and the effects of periplocin on gemcitabine-resistant pancreatic cancer.......... S69
Figure S61. CETSA-based determination of thiogochangamide B-β-catenin protein binding. S70
Figure S62. Experimental validation of the inhibitory effect of β-catenin knockdown on cell proliferation. S71
Figure S63. Effects of thiogochangamide B on cell cycle distribution in PANC-GR cells.......... S72
Figure S64. Induction of cell death by thiogochangamide B treatment S73
Figure S65. Effects of β-catenin knockdown on cell migration, invasion and EMT marker in pancreatic cancer cells S74
Figure S66. The changes of mouse body weight... .... S75
Figure S67. Calculation of the synergistic anti-tumor effect of thiogochangamide B in combination with gemcitabine in a gemcitabine-resistant pancreatic cancer xenograft model.. .... S76

Isothermal titration calorimetry (ITC) assay S77
Figure S68. Isothermal titration calorimetry (ITC) assay using recombinant β-catenin and thiogochangamide B. S77

***In vitro* metabolic stability.......... S78**
Figure S69. *In vitro* metabolic and proteolytic stability of thiogochangamide B S78

References. S79

Methods

General experimental procedures

Optical rotations were determined using a JASCO P-2000 polarimeter (JASCO, Easton, PA, USA) with a 1 cm path length cell. Ultraviolet (UV) and circular dichroism (CD) spectra were recorded using an Applied Photophysics Chirascan-Plus CD spectrometer (Applied Photophysics, Leatherhead, Surrey, UK) with a 1 mm CD cell. Infrared (IR) spectra were obtained using a JASCO Fourier-transform infrared (FT/IR) spectrometer (JASCO, Easton, PA, USA). An Agilent Technologies 6130 Quadrupole mass spectrometer (Agilent Technologies, Santa Clara, CA, USA) with an electrospray ionization (ESI) source, combined with an Agilent Technologies 1200 series high-performance liquid chromatography (HPLC) system (Agilent Technologies, Santa Clara, CA, USA) and a reversed-phase $C_{18}(2)$ column (Luna, 4.6 mm × 100 mm, 5 µm, Phenomenex, Torrance, CA, USA) were utilized for chemical analysis. High-resolution electrospray ionization mass spectrometry (HR-ESI-MS) data for the bacterial extracts were acquired using a Q Exactive nano-High resolution LC/MSMS spectrometer (Thermo Fisher Scientific, Waltham, MA, USA) at the National Instrumentation Center for Environmental Management (NICEM), College of Agriculture and Life Sciences, Seoul National University. Nuclear magnetic resonance (NMR) spectra, including ^{1}H, ^{13}C, and two-dimensional (2D) NMR, were recorded on Bruker Avance III HD 800. The polarimeter, CD spectrometer, UV spectrometer, FT/IR spectrometer, 800 MHz NMR spectrometer were all located at the College of Pharmacy, Seoul National University, Seoul, Republic of Korea. High-resolution electrospray ionization mass spectrometry (HR-ESI-MS) data of the purified compounds were collected using an AB SCIEX 5600 QTOF HR-MS instrument (AB SCIEX, Framingham, MA, USA) at the National Instrumentation Center for Environmental Management (NICEM), College of Agriculture and Life Sciences, Seoul National University. Bacterial strains were cultured in a Compact Shaking Incubator (JS Research, Gongju, Chungcheongnam-do, Republic of Korea) until they reached mid-log phase, as measured by Ultrospec 10 (Biochrom, Cambridge, UK). Frozen stocks were stored in TDE Series Ultra-Low Temperature Freezers (Thermo Fisher Scientific, Waltham, MA, USA). *In vitro* mycobacterial infections were performed using a benchtop centrifuge (Eppendorf, Hamburg, Germany), a CLASS II Type A2 Biological safety cabinet (CHC Lab, Daejeon, South Korea), a BB 150 CO_2 incubator (Thermo Fisher Scientific, Waltham, MA, USA), and a General Incubator (JS Research, Gongju, Chungcheongnam-do, Republic of Korea).

Bacterial isolation, cultivation and extraction

Streptomyces sp. TD3 was isolated from Alaskan permafrost soil. *Streptomyces* sp. TD3 (GenBank accession number PP264157) was cultured in 50 mL TSBY liquid medium (17.0 g tryptone, 3.0 g soytone, 2.5 g glucose, 5.0 g NaCl, 2.5 g K_2HPO_4, 3.0 g yeast, 1 L deionized water) in a 100 mL Erlenmeyer flask. After cultivation for 3 days on a rotary shaker at 200 rpm and 30 °C, 5 mL of the culture medium was inoculated in 200 mL of R4 medium (5.0 g glucose, 1.0 g yeast extract, 5.0 g $MgCl_2 \cdot 6H_2O$, 2.0 g $CaCl_2 \cdot 2H_2O$, 1.5 g proline, 1.2 g valine, 3.0 g TES, 50 mg casamino acid, 100 mg K_2SO_4, 1.0 mL trace element solution (40 mg/L $ZnCl_2$, 200 mg/L $FeCl_3 \cdot 6H_2O$, 10 mg/L $CuCl_2 \cdot 2H_2O$, 10 mg/L $MnCl_2 \cdot 4H_2O$, 10 mg/L $Na_2B_4O_7 \cdot 10H_2O$, 10 mg/L $(NH_4)_6Mo_7O_{24} \cdot 4H_2O$)) in a 500 mL Erlenmeyer flask. Sixteen litres of the TD3 culture was prepared in the same manner and extracted with 30 L of ethyl acetate after 8 days of incubation. The ethyl acetate was removed *in vacuo* to afford 1.2 g of dry extract.

Soil sample was collected from Gochang wetland, Republic of Korea, in May 2020. The sample was diluted in sterile water and spread onto various agar media for strain isolation (actinomycete isolation agar medium, YPM agar medium, A4 agar medium, K agar medium, Czapek-Dox agar medium, ISP1 agar medium, ISP4 agar medium, and all media were supplemented with 100 mg/L of cycloheximide), and the plates were incubated at 30°C for two weeks. Single strains, GC2 was isolated on Czapek-Dox agar medium. Analysis of 16S rRNA gene sequences showed that strain GC2 was most closely related to *Streptomyces* sp. gene (100% identity, GenBank accession No. OQ359407). The GC2 strain was cultured in 50 mL modified K medium (4 g yeast extract, 5 g malt extract, 5 g soytone, 5 g soluble starch, 5 g mannitol, 2 g glucose, and 6 g glycerol in 1 L deionized water) in a 125 mL Erlenmeyer flask. After cultivation for 2 days on a rotary shaker at 200 rpm and 30 °C, 5 mL of the culture medium was inoculated in 200 mL of modified K medium in a 500 mL Erlenmeyer flask. After cultivation for 2 days under the same incubation conditions, 15 mL of the culture medium was inoculated in 1 L of modified K medium in 2.5 L ultra-yield flasks at 170 rpm and 30 °C for 6 days. Two hundred liters of the GC2 culture was extracted with 300 L of ethyl acetate. The ethyl acetate and water were separated, and the remaining water in the ethyl acetate layer was removed by adding anhydrous sodium sulfate. The ethyl acetate was removed using a rotary evaporator, yielding 50 g of dry extract.

Purification of oxidized-thiostreptamide S4

The crude extract was chromatographed over a C_{18} reversed–phase open column, eluting with 20, 40, 60, 80, and 100% MeOH–H_2O to afford five fractions. The 80% and 100% MeOH-H_2O fractions were combined and further purified using the reversed-phase HPLC (YMC-Pack ODS-A column, 250 × 10 mm, flow rate: 2 mL/min, detection: UV = 210, 270 nm) using a gradient solvent system from 43% to 61% CH_3CN-H_2O with 0.1 formic acid over 45 min, to yield the oxidized-thiostreptamide S4 (t_R 30.5 min, 2.1 mg).

Purification of thiogochangamides

The crude extract was chromatographed over a C_{18} reversed–phase open column, eluting with 40, 60, 80, and 100% MeOH–H_2O to afford four fractions. The 80% and 100% MeOH-H_2O fractions were injected directly into a semipreparative reversed-phase HPLC (Kromasil C_{18}, 5 µm, 250 × 10 mm, flow rate: 2 mL/min, detection: UV = 270 nm) using a gradient solvent system from 30% to 60% CH_3CN-H_2O with 0.1 formic acid over 40 min. Thiogochangamides A and B (**1** and **2**) were isolated at 29 and 31 min, respectively. They were further purified on an IB chiral HPLC column (CHIRALPAK, 5 µm, 250 × 4.6 mm) using isocratic conditions (40% CH_3CN-H_2O, UV detection at 270 nm, flow rate: 0.7 mL/min) to yield, **1** (t_R 30 min, 6.8 mg) and **2** (t_R 33 min, 45.0 mg).

Thiogochangamide A (1): colorless powder; $[\alpha]^{20}_{D}$ -74.2 (*c* 0.1, MeOH); UV (MeOH) λ_{max} (log ε) 270 (2.06) nm; IR ν_{max} 3308, 2929, 1669, 1600, 1523 cm^{-1}; For ^{1}H, ^{13}C NMR and 2D NMR data, see Table S (800MHz, Acetonitrile-d_3) ; HRESIMS *m/z* 1291.5126 $[M+H]^+$ (calcd. for $C_{56}H_{87}N_{14}O_9S_6^+$, 1291.5099).

Thiogochangamide B (2): colorless powder; $[\alpha]^{20}_{D}$ -101.7 (*c* 0.1, MeOH); UV (MeOH) λ_{max} (log ε) 271 (1.87) nm; IR ν_{max} 3276, 2967, 1668, 1598, 1522 cm^{-1}; For ^{1}H, ^{13}C NMR and 2D NMR data, see Table S (800MHz, Acetonitrile-d_3) ; HRESIMS *m/z* 1307.5058 $[M+H]^+$ (calcd. for $C_{56}H_{87}N_{14}O_{10}S_6^+$, 1307.5048).

Acid hydrolysis and FDAA derivatization of thiogochangamides A and B

1.5 mg of thiogochangamide A (**1**) and B (**2**) was dissolved in 1 mL of 6N HCl and heated at 115 °C for 1 hour. The heated suspension was cooled in ice bath for 3 minutes, and the HCl was vaporized *in vacuo*. To eliminate residual HCl, 1 mL of water was added to the vial and then evaporated under low pressure two times. Subsequently, the hydrolysate mixture was lyophilized for 24 hours and divided into two vials. Each hydrolysate sample was dissolved in 480 µL of 1 N $NaHCO_3$. Then, 2 mg of L-FDAA and D-FDAA were added to each vial. The two reaction vials were heated at 80 °C for 3 min to accelerate the chemical derivatization. Then, 80 µL of 6 N HCl was added to neutralize both reaction mixtures, and the they were diluted with 500 µL of methanol. Ten microliters of each reaction mixture was injected into the LC/MS under a gradient solvent system (flow rate: 0.7 mL/min; UV detection: 360 nm; 10% to 60% CH_3CN/H_2O with 0.1% formic acid over 40 min) with a reversed-phased column (Phenomenex Luna, 100 × 4.6 mm, $C_{18}(2)$, 5 µm). The retention times of the FDAA derivatives of the free amino acids were observed via LC/MS analysis.

Partial hydrolysis of thiogochangamide B

To determine the absolute configurations of the multiple alanine residues in thiogochangamide B (**2**), we employed a partial hydrolysis approach. After extensive optimization involving various acid concentrations and reaction times, many of which resulted in complete degradation of the compound, we successfully identified suitable conditions for selective hydrolysis. The optimized conditions are detailed as follows.

Thiogochangamide B (**2,** 2.0mg) was hydrolyzed in 3N HCl at 60 °C for 17 hours. The desired fragment, which contains the cyclic structure on the right side of **2**, was detected by LC/MS. Then, the reaction mixture was dried *in vacuo* and subjected to the semi-preparative reversed-phase HPLC (Kromasil C_{18}, 5 µm, 250 × 10 mm). The cyclic partial structure was eluted at 28 min using a gradient solvent system (10%-100% CH_3CN-H_2O over 80 min, flow rate: 2 mL/min, detection: UV 210, 254 nm, HR-ESI-MS $[M+H]^+$ *m/z* at 657.3177 $C_{31}H_{45}N_8O_6S^+$, calcd $[M+H]^+$ *m/z* at 657.3177). To determine the absolute configurations of the alanine residues in the linear part, it was necessary to modify the hydrolysis conditions. By following the reference determining the absolute configuration of kahalalide F,[1] Thiogochangamide B (**2,** 2.0mg) was hydrolyzed with 1N HCl and EtOH 1:1 mixture at 60 °C for 2.5 hours. The fragment including the partial linear structure on the left side of **2** was detected by LC/MS. The desired fragment was purified at 23 min by the semi-preparative reversed-phase HPLC (Kromasil C18, 5 µm, 250 × 10 mm) using a gradient solvent system (20%-50% CH_3CN-H_2O over 80 min, flow rate: 2 mL/min, detection: UV 210nm, HR-ESI-MS $[M+H]^+$ *m/z* at 902.3826 $C_{40}H_{60}N_{11}O_7S_3^+$, calcd $[M+H]^+$ *m/z* at 902.3834) (Figure S40). The absolute configurations of the multiple alanine were successfully determined by subjecting each purified partial structure to complete acid hydrolysis followed by FDAA derivatizations and LC/MS analysis as shown above in the acid hydrolysis and FDAA derivatization section.

Mosher's method of thiogochangamide B

Thiogochangamide B (**2**) was transferred into two 40 mL vials (1.5 mg of **2** for each) and dried completely under high vacuum overnight. Under argon gas, the compound was dissolved in 1 mL of distilled anhydrous pyridine, and then 25 µL of *S*-α-methoxy-α-(trifluoromethyl) phenylacetyl chloride (*S*-MTPA-Cl) was added. The reaction mixture was stirred at room temperature for 60 min and the reaction was quenched by adding 200 µL of MeOH. The reaction mixture was dried *in vacuo* and purified by the semi-preparative reversed-phase HPLC (Kromasil C_{18}, 5 µm, 250 × 10 mm). *R*-MTPA-ester of **2** (**7**) was eluted at 39 min using a gradient solvent system (30%-80% CH_3CN-H_2O over 50 min, flow rate: 2 mL/min, detection: UV 254 nm) and further purified under the isocratic HPLC conditions (40% CH_3CN-H_2O, flow rate: 2.0 mL/min, detection: UV 254 nm, eluted at 35min). Derivatization with *R*-α-methoxy-α-(trifluoromethyl)phenylacetyl chloride (*R*-MTPA-Cl) was proceeded in the same manner as described for *S*-MTPA-Cl, but *S*-MTPA-ester of **2** easily degraded. After several trials and errors, the reaction was performed for only one minute after adding *R*-MTPA-Cl and quenched right away. In this way, *S*-MTPA-ester (**6**) was successfully obtained and isolated. The delta values ($\Delta\delta_{S-R}$) of the ^{1}H signals around the stereogenic center was assigned by analyzing ^{1}H NMR and ^{1}H-^{1}H COSY NMR spectra.

S-MTPA ester of thiogochangamide (**6**): ^{1}H NMR (800 MHz, Acetonitrile-*d*$_3$) δ_H 8.936 (1H, br s), 8.299 (1H, s), 7.000 – 8.000 (10H, aromatic protons overlap), 7.678 (1H, d, *J* = 8.0 Hz), 7.564 (1H, s), 7.320 (1H, m), 7.198 (1H, br s), 5.749 (1H, d, *J* = 9.0 Hz), 5.597 (1H, d, *J* = 6.0 Hz), 5.587 (1H, m), 5.473 (1H, m), 5.336 (1H, m), 4.739 (1H, m), 4.694 (1H, q, *J* = 7.0), 4.556 (1H, m), 4.547 (1H, m), 4.377 (1H, m), 4.205 (1H, m), 4.070 (1H, m), 3.912 (3H, s), 3.816 (3H, s), 3.815 (1H, m), 3.796 (1H, m), 3.584 (3H, s), 3.170 (1H. m), 3.150 (1H, m), 2.843 (1H, m), 2.683 (1H, m), 2.564 (1H, m), 2.456 (1H, m), 2.316 (1H, m), 2.280 (1H, m), 2.063 (3H, s), 2.061 (1H, m), 1.735 (3H, d, *J* = 7.0 Hz), 1.706 (3H, d, *J* = 7.0 Hz), 1.540 (3H, d, *J* = 7.0 Hz), 1.510 (3H, d, *J* = 7.5 Hz), 1.458 (3H, d, *J* = 7.0 Hz), 1.384 (3H, d, *J* = 7.5 Hz), 1.088 (3H, d, *J* = 7.0 Hz), 1.066 (3H, d, *J* = 7.0 Hz), 0.984 (3H, d, *J* = 7.0 Hz), 0.961 (3H, d, *J* = 6.5 Hz), 0.914 (3H, d, *J* = 6.5 Hz), 1 OH and 9 NH are undistinguishable. HR-ESI-MS $[M+H]^+$ *m/z* at 1523.5447 ($C_{66}H_{94}F_3N_{14}O_{12}S_6$), calcd $[M+H]^+$ *m/z* at 1523.5455.

R-MTPA ester of thiogochangamide (**7**): ^{1}H NMR (800 MHz, Acetonitrile-*d*$_3$) δ_H 10.543 (1H, br s), 10.400 (1H, br s), 9.891 (1H, br s), 8.868 (1H, d, *J* = 6.0 Hz), 8.813 (1H, br s), 8.305 (1H, br s), 7.000 – 8.000 (10H, aromatic protons overlap), 7.892 (1H, br s), 7.744 (1H, br s), 7.740 (1H, br s), 7.622 (1H, br s), 7.592 (1H, overlap), 7.319 (1H, m), 7.257 (1H, overlap), 7.173 (1H, br s), 6.676 (1H, d, *J* = 6.0 Hz), 5.762 (1H, d, *J* = 9.0 Hz), 5.618 (1H, d, *J* = 7.0 Hz), 5.571 (1H, m), 5.519 (1H, m), 5.315 (1H, m), 4.716 (1H, m), 4.676 (1H, m), 4.551 (1H, m), 4.474 (1H, m), 4.374 (1H, m), 4.168 (1H, m), 4.072 (1H, m), 3.906 (3H, s), 3.824 (3H, s), 3.818 (1H, m), 3.802 (1H, m), 3.568 (3H, s), 3.158 (1H, m), 3.121 (1H, m), 2.818 (1H, m), 2.690 (1H, m), 2.554 (1H, m), 2.296 (2H, m), 2.265 (1H, m, overlap), 2.070 (3H, s), 2.050 (1H, m, overlap), 1.747 (3H, d, *J* = 7.0 Hz), 1.726 (3H, d, *J* = 7.0), 1.536 (6H, d, *J*= 7.0, CH_3*2), 1.510 (3H, d, *J*= 7.0), 1.399 (3H, d, *J*= 7.0), 1.089 (3H, d, *J*= 7.5), 1.077 (3H, d, *J*= 7.0), 0.982 (3H, d, *J*= 7.0), 0.923 (3H, d, *J*= 7.0 Hz), 0.858 (3H, d, *J* = 7.0 Hz), 1 OH are undistinguishable. HR-ESI-MS $[M+H]^+$ *m/z* at 1523.5447 ($C_{66}H_{94}F_3N_{14}O_{12}S_6$), calcd $[M+H]^+$ *m/z* at 1523.5431.

Amidation (desulfurization) of thiogochangamide B

Thiogochangamide B (**2**) (2.0 mg, 1.54 µmol, 1.0 equiv.) and $ZrCl_4$ (1.76 mg, 7.70 µmol, 5.0 equiv.) were added to a 4 mL vial and then, the solid was dissolved in EtOH (0.5 mL). H_2O_2 (35%, 0.66 µL, 7.70 µmol, 5.0 equiv.) was added dropwise to the mixture with stirring at rt for 30 min.[2] The solution was directly purified on a reversed-phase HPLC column (Kromasil C_{18}, 5 µm, 250 × 10 mm) using the isocratic conditions (70% MeOH-H_2O, UV detection at 280 nm, flow rate: 2 mL/min, t_R 40 min) to afford amidized thiogochangamide B (0.7 mg, 35.0% yield).

Chemical synthesis of 4-(2-amino-2-carboxyethyl)-1,3-dimethyl-1H-imidazol-3-ium

Synthesis of 4-(2-benzamido-3-methoxy-3-oxopropyl)-1,3-dimethyl-1H-imidazol-3-ium (3a)

Bz-his-Ome (5.0 g, 18.3 mmol, 1.0 equiv.) and anhydrous K_2CO_3 (4.2 g, 30.0 mmol, 1.64 equiv.) were added to a dry round bottomed flask (250 mL). They were dissolved in dry acetone (100 mL) and then, dimethyl sulfate (5.0 mL, 52.5 mmol, 2.87 equiv.) was added to the solution. The flask was equipped with a reflux condenser and the reaction mixture was heated to 60 °C for 24 h in a heat block. After complete reaction, mixture was filtered and the filtrate was concentrated *in vacuo*. The yellow oil (**3a**, 6.8 g) was used in the next step without purification.

Synthesis of 4-(2-amino-2-carboxyethyl)-1,3-dimethyl-1H-imidazol-3-ium (3b)

3a (8.3 mg, 0.02 mmol, 1.0 equiv.) was added to a dry 4.0 mL vial. 6 N HCl (0.4 mL) was added to a vial and then, the solution was heated to 100 °C for 8 h under the heat block. After the solution was concentrated *in vacuo*, the residue was purified on a reversed-phase HPLC column (Kromasil C18, 5 μm, 250 × 10 mm) using the isocratic conditions (5% CH_3CN-H_2O, UV detection at 210m, flow rate: 2 mL/min, , t_R 7.0 min) to afford **3b** (4.1 mg, 93.3%).

Conformational search, DP4 and CP3 calculation analysis

Computational analysis was conducted following the established protocols. Conformational searches for each plausible diastereomer were carried out using MacroModel (Maestro suite, version 9.9; Schrödinger LLC, New York, NY, USA) employing the "Mixed Torsional/Low-Mode Sampling" method with the Merck Molecular Force Field (MMFF). Each search was performed in the gas phase with an energy window of 50 kJ/mol and a maximum of 10,000 steps to ensure thorough sampling of low-energy conformers. Energy minimization of the resulting conformers was conducted using the Polak–Ribiere Conjugate Gradient (PRCG) method with a maximum of 10,000 iterations and a convergence threshold of 0.001.Subsequently, the gauge-including atomic orbital (GIAO) NMR shielding tensors were calculated for all conformers within 10 kJ/mol of the global minimum using TmoleX (version 4.3.2; COSMOlogic GmbH & Co., Leverkusen, Germany) at the B3LYP/def-SV(P) level of theory in the gas phase. These values were averaged by the Boltzmann population based upon their MMFF force field energies and the corresponding chemical shift values were calculated according to the equations described in original study and used for DP4 and CP3 probability analysis elaborated in the main text.[3, 4]

PCR primer designing and rapid PCR screening with the gDNA library

PCR primers were designed based on the previously-reported *Streptomyces* sp. TfuA protein-encoding genes. TfuA-like proteins from *Streptomyces* sp. [>BBC15205.1], *Streptomyces* NRRL S-87 [>WP_030193564.1], *Streptomyces malaysiense* [>WP_046417234.1], *Streptomyces* sp. MUSC 14 [>WP_071375572.1] and *Streptomyces roseoverticillatus* [>WP_235488553.1] were used as references. Each amino acid sequence was reverse translated into nucleotide sequence using Sequence Manipulation Suite website. The nucleotide sequences of the genes encoding TfuA were collected and degenerate primers for amplifying conserved sequences were designed based on the aligned nucleotide sequences. The primer optimization process was conducted as follows. To ensure specificity and binding efficiency, primer lengths were designed to range from 17 to 20 bp, and melting temperatures (Tm) were varied between 50 and 65 °C by adjusting the GC content to 45–65%. Primers were designed to avoid the repetition of the same nucleotide more than four consecutive times. Based on conserved regions, a total of six forward primers and four reverse primers were designed (Table S12). Primer sets generating amplicons smaller than 220 bp were excluded, and the remaining primer combinations were experimentally tested. Among these, the indicated primer set produced the strongest and most distinct amplification band. (TfuA forward : 5′-ATCGAYGCMCTCATGGC-3′, TfuA reverse : 5′-GATGTTGACCATGGGTTCG-3′). The predicted amplicon sequence size was 306 bp. The designed primers were validated by PCR using genomic DNA extracted from *Streptomyces* sp. TD3, which was previously analyzed to produce thioamide compound by whole genome sequencing (thioviridamide like compounds). PCR screening with the TfuA primer set was performed using the following methods : denaturation 95 °C for 30 s, annealing at 60 °C for 30 s, extension at 72 °C for 1 min, and final extension at 72 °C for 5 min. This cycle was repeated 30 times. The PCR products were separated through electrophoresis on 1% agarose gels, and 21 strains amplicon bands about 300 bp were purified and sequenced. Finally, screening the in-house gDNA library (1,192 strains) selected 2 strains (GC2 and DS4). These strains were cultured to produce thioamide compounds.

Whole genome sequencing and genomic analysis

Genome sequencing of *Streptomyces* sp. TD3 and GC2 was performed by ChunLab Inc. (Seoul, Republic of Korea) using the PacBio RS II system. Genome assembly was conducted using PacBio SMRT Analysis 2.3.0 with the HGAP protocol (Pacific Biosciences, USA). Functional annotations of the genome were carried out with EggNOG 4.5, Swissprot, KEGG, and SEED as references based on ChunLab's in-house pipeline. Secondary metabolite biosynthetic gene clusters were identified and analyzed using antiSMASH 7.0.0. The software Geneious prime® 2022.1.1 was utilized to perform sequence alignments.

Cell culture conditions

Human pancreatic cancer cell line (PANC-1) was provided from Korean Cell Line Bank (Seoul, Korea). The gemcitabine-resistant cell line PANC-GR was established in *in vitro* system by exposing gemcitabine to PANC-1 cell line with 0.1–2 μM. PANC-1, PANC-1-GR and HEK293 were maintained in Dulbecco's modified Eagle's medium (DMEM), 10% fetal bovine serum (FBS) and supplemented with penicillin – streptomycin (sodium penicillin G: 100 units/mL; streptomycin: 100 μg/mL) at 37 °C in a humidified incubator with 5% CO_2.

Drugs and chemical compounds

All reagents used for cell culture, including culture media, fetal bovine serum, trypsin-EDTA solution (1 ×), and penicillin–streptomycin solution (100 ×), were purchased from Gibco (Grand Island, NY, USA). Dimethyl sulfoxide (DMSO), sulforhodamine B (SRB), trichloroacetic acid (TCA), bicinchoninic acid, copper (II) sulfate solution, bovine serum albumin (BSA), gemcitabine, and propidium iodide (PI) were purchased from Sigma-Aldrich (St. Louis, MO, USA). Laemmli sample buffer (2 ×) and 2-mercaptoethanol were purchased from Bio-Rad Laboratories, Inc. (Hercules, CA, USA). The anti-β-Actin (sc-47778) was purchased from Santa Cruz Biotechnology, Inc. (Dallas, TX, USA). Anti-β-catenin (8480S), Anti-p-β-catenin (9564S), Anti-AXIN1 (2087S), Anti-c-Myc (13987S), Anti-Cyclin D1 (55506S), Anti-Survivin (2808S), Anti-α-tubulin (2144S), anti-PARP (9542S), anti-N-cadherin (13116S), anti-MMP7 (3801S), anti-Snail (3879S), anti-E-cadherin (3195S), anti-CDK4 (12790S), anti-CDK6 (13331S), anti-Cyclin E1 (20808S), anti-p21 (2947S), anti-cleaved caspase 8 (8592S), anti-cleaved caspase 9 (9509S) were purchased from Cell Signaling Technology (Danvers, MA, USA).

Growth inhibition assay

To evaluate the growth-inhibitory effects of test compounds on various cancer cell lines, a sulforhodamine B (SRB) assay was performed. Cells were seeded in 96-well plates at a density of 6 × 10^4 cells/mL and incubated either for 30 minutes (to establish day 0 controls) or treated with test compounds at concentrations ranging from 0 to 50 µM for 72 h. Following treatment, cells were fixed with trichloroacetic acid (TCA) for 30 minutes, air-dried, and stained with 0.4% (w/v) SRB in 1% acetic acid for 2 h. After staining, excess dye was removed, and the bound dye was solubilized in 10 mM Tris base (pH 10.0). Absorbance was measured at 515 nm using a microplate reader. Cell proliferation was calculated using the following formula:

Cell proliferation (%) = [(A_sample − A_day0) / (A_control − A_day0)] × 100, where *A_sample*, *A_day0*, and *A_control* represent the average absorbance of the treated sample, day 0 control, and untreated control, respectively. The half-maximal inhibitory concentration (IC_{50}) values were determined by nonlinear regression analysis using TableCurve 2D v5.01 software.[5]

Bioinformatics Analysis

The Overall Survival (OS) of Disease-free survival of patients with pancreatic cancer with *CTNNB1* (β-catenin) expression was analyzed by online-based Kaplan-Meier plotter (https://kmplot.com/ analysis/, accessed 02 November 2023).

Western blot analysis

PANC-GR cells were seeded and treated with thiogochangamide B (**2**) for 24 h. After incubation, total cell lysates were prepared using lysis buffer and boiled at 100 °C for 12 min. Protein concentrations were determined using the Pierce™ BCA Protein Assay Kit, and equal amounts of protein were separated by SDS-polyacrylamide gel electrophoresis (SDS-PAGE). The proteins were then transferred onto polyvinylidene fluoride (PVDF) membranes (Millipore, Bedford, MA) that had been activated with 100% methanol. Membranes were blocked with 5% bovine serum albumin (BSA) in Tris-buffered saline containing 0.1% Tween-20 (TBST) for 30 min at room temperature. Primary antibodies, diluted 1:200 to 1:2000 in 5% BSA, were applied and incubated overnight at 4 °C. After three washes with TBST, membranes were incubated with appropriate HRP-conjugated secondary antibodies diluted in TBST for 2 h at room temperature. After three additional washes, signals were detected using an enhanced chemiluminescence (ECL) detection kit (iNtRON Biotechnology, Seongnam, Korea) and visualized with the ImageQuant LAS 4000 system (GE Healthcare, Chicago, IL).

Transfection and Luciferase Reporter Gene Assay

Transient transfections were performed using Lipofectamine 2000 (Invitrogen) according to the manufacturer's protocol. HEK293 and PANC-GR cells were seeded in 48-well plates and transfected with 0.1 µg of a luciferase reporter plasmid (TOPflash or FOPflash) and 0.005 µg of a Renilla luciferase plasmid for normalization. To activate the Wnt/β-catenin pathway, HEK293 cells were additionally co-transfected with 0.02 µg of pcDNA–β-catenin expression vector and 0.004 µg of a TCF4 expression vector. After 24 h, cells were treated with the test compound and incubated for an additional 24 h. Cells were then lysed, and luciferase activity was measured using the Dual-Luciferase Reporter Assay System (Promega), following the manufacturer's instructions. pTOP/FOPflash and Renilla plasmids were obtained from Upstate Biotechnology, and TCF4 and pcDNA–β-catenin expression vectors were generously provided by Dr. Meeldijk (University Medical Center). Relative luciferase activity was calculated by normalizing firefly luciferase activity to Renilla luciferase, and results were expressed relative to the vehicle-treated control.[6]

Cellular Thermal Shift Assay

To assess the binding affinity of thiogochangamide B (**2**) within the cellular context, a cellular thermal shift assay (CETSA) was performed. PANC-GR cells were seeded in 100-mm culture dishes and treated with 10 µM thiogochangamide B for 1 h in a humidified incubator at 37 °C. Following treatment, cells were harvested and aliquoted into 12 individual 0.2 mL PCR tubes (100 µL each). The samples were then subjected to a thermal gradient using a thermal cycler according to the instrument's settings. After heating, samples underwent three freeze–thaw cycles using liquid nitrogen to lyse the cells. Lysates were transferred to 1.5 mL microcentrifuge tubes and centrifuged at 17,000 × g for 40 minutes at 4 °C. Supernatants (60 µL) were carefully collected from each sample and mixed with 20 µL of 4× LDS sample buffer and 4 µL of β-mercaptoethanol (β-ME). The soluble protein fractions were analyzed by Western blotting to assess protein stability.[7]

RNA Interference

RNA interference targeting β-catenin was performed using a pool of three siRNA duplexes (Bioneer, Daejeon, Korea). PANC-GR cells were transfected with 10 nM siRNA duplexes using Lipofectamine RNAiMAX (Invitrogen, Grand Island, NY, USA) according to the manufacturer's protocol. Transfection was carried out for 24 h, after which cells were treated with the test compound. A scrambled siRNA duplex was used as a negative control.

Wound healing migration assay

PANC-1 and PANC-GR cells (parental and gemcitabine-resistant, respectively) were seeded in six-well plates and grown to approximately 95% confluence. A scratch was introduced into the cell monolayer using an SPL Scar Scratcher (SPL Life Sciences, Pocheon, Republic of Korea), and detached cells were removed by washing with PBS. Cells were then incubated in medium supplemented with 1% FBS and various concentrations of thiogochangamide B (**2**) for 24 h. Images of the wounds were captured at 0 and 24 h using an inverted microscope (Olympus, Tokyo, Japan). Wound areas were quantified using ImageJ software (version 1.52a) and migration was expressed as percent wound closure relative to the wound area at 0 h.

Transwell cell invasion assay

A 24-well Transwell membrane inserts (diameter, 6.5 mm; pore size, 8 µm; Corning, Tewksbury, MA, USA) was used to assess cell invasion. Inserts were coated with 15 µL of matrigel (BD Biosciences, San Diego, CA, USA) and 20 µL of a 1:20 dilution of Matrigel (BD Biosciences) in PBS. After treatment with thiogochangamide B for 24 h, PANC-1 or PANC-GR cells (parent or gemcitabine-resistant, respectively) were harvested, resuspended in serum-free medium, and seeded in to the upper chamber of the coated inserts at a density of 3 × 10^5 cells per chamber. Medium containing 10% FBS was added to the lower chamber as a chemoattractant. After 24 h of incubation, cells that had invaded to the underside of the membrane were fixed with 4% paraformaldehyde and stained with 0.1 % crystal violet solution. Invasive cells were imaged using the Vectra 3.0 Automated Quantitative Pathology Imaging System (PerkinElmer, Waltham, MA, USA). Representative images from three independent experiments

were analyzed, and the number of invading cells was semiquantified using ImageJ 1.52a software (National Institutes of Health, Bethesda, MD, USA).

Synchronization and cell cycle analysis
For cell cycle analysis, PANC-GR cells were seeded and treated with thiogochangamide B (**2**) for 24 h and washed with PBS, and then the cell pellets were fixed with 70% ethanol overnight at −20°C. Fixed cells were trypsinized and washed with PBS. Cell cycle pattern by DNA contents of PANC-GR cells, GR cells were harvested at 24 h after thiogochangamide B treatment and subjected to PI staining with 50 µg/mL Rnase A followed by flow cytometry (FACScalibur, BD Bioscience, Franklin Lakes, NJ) analysis. The distribution of cell contents was measured with 10,000 cells in each group and the results were represented as histograms of the DNA content.

Annexin V-FITC/Propidium Iodide (PI) Double Staining Analysis
PANC-GR cells were treated with thiogochangamide B (**2**) for 48 h and stained with Annexin V-FITC and propidium iodide (PI) using an Annexin V-FITC Apoptosis Detection Kit (BD Biosciences, San Diego, CA) according to the manufacturer’s instructions. Briefly, treated cells were harvested and resuspended in 1× binding buffer. Annexin V-FITC and PI (5 µL each) were added to the cell suspension, followed by incubation in the dark for 15 min at room temperature. After staining, cells were immediately analyzed by flow cytometry in 1× binding buffer.

In vivo drug efficacy assessment in mice
All animal handling and animal-related procedures were performed according to the guidelines approved by the Seoul National University Institutional Animal Care and Use Committee (IACUC permission number: SNU-220622-3-1). Detailed protocol was followed as previously described.[4] The 6 weeks-old male nude mice (Balb/c-nu, weighing ~23 g) were purchased from Orient Bio, Inc (Seongnam, Korea) and maintained under pathogen-free conditions in the animal facilities at Seoul National University, College of Pharmacy. PANC-1 and PANC-GR cells were subcutaneously injected (1×10^7 cells per mouse mix with Matrigel 1:1) into the right flank of mice and tumors were maintained until tumor volume reached 180 mm^3. The mice were randomly divided into four groups (n=5) and intraperitoneally administered vehicle solution (5 % of DMSO, 5 % Kolliphor and 90 % saline), gemcitabine (20 mg/kg), thiogochangamide B (1 mg/kg), or comination group (thiogochangamide B 2 mg/kg and gemcitabine 20 mg/kg) three times a week for 23 days. The tumor volume was monitored using a caliper and calculated following formula: Tumor volume (mm^3) = (width) × (length) × (height) × π/6. The body weight of each mouse was also monitored.
Analysis of the synergistic effect between thiogochangamide B and gemcitabine on tumor xenograft growth was performed using the combination ratio method.[8] Individual relative tumor volume (RTV) was calculated as the ratio of tumor volume on the measurement day after treatment to the tumor volume at the initiation of treatment. The tumor growth inhibition rate was calculated as:
Inhibition rate (%) = (1 − final RTV of treated group / final RTV of vehicle group) × 100. To evaluate the combined effect, the fractional tumor volume (FTV) was defined as the ratio of the mean final tumor volume in the drug-treated group to that of the vehicle-treated group. The expected FTV under the assumption of no interaction (i.e., additive effect) was calculated as:
Expected FTV = (FTV of thiogochangamide B) × (FTV of gemcitabine).
The observed FTV was calculated as:
Observed FTV = final tumor volume of combination group / final tumor volume of vehicle group.
The combination ratio was calculated as:
Combination ratio = expected FTV / observed FTV.
A combination ratio greater than 1 indicates a synergistic effect, whereas a ratio less than 1 suggests an antagonistic or less-than-additive effect.

Statistical Analysis
All quantitative data are presented as mean ± standard deviation (SD) from the indicated number of independently performed experiments. No data transformation or normalization was applied prior to statistical analysis, and no outliers were excluded. Each experiment was independently repeated at least three times unless otherwise stated. The sample size (n) represents the number of independent biological replicates used for each analysis. Statistical significance was determined using a two-tailed Student’s *t*-test for comparisons between two groups or one-way analysis of variance (ANOVA) followed by Dunnett’s post hoc test for multiple comparisons against a control group. A P value < 0.05 was considered statistically significant ($P < 0.05$, *$P < 0.01$, **$P < 0.001$). All statistical analyses were performed using GraphPad Prism software.

Isothermal Titration Calorimetry (ITC)
ITC measurements were carried out on a MicroCal PEAQ-ITC instrument (Malvern Panalytical, Worcestershire, UK). Recombinant β-catenin was dialyzed overnight at 4°C against ITC buffer consisting of 10 mM HEPES (pH 7.0), 150 mM NaCl, and 0.5 mM TCEP. Thiogochangamide B was directly solubilized in ITC buffer. Thiogochangamide B (330 µM) loaded in the syringe was titrated into β-catenin (10 µM) in the ITC cell with a total volume of 200 µL. Titrations were carried out at 25°C with stirring at 750 rpm and consisted of 12 injections of 2 µL each (2 s per injection), with ~200 s intervals between injections. Data were analyzed using the program MicroCal PEAQ-ITC Analysis Software (Malvern Panalytical, Worcestershire, UK).

In vitro metabolic stability in liver S9 fraction
Metabolic stability was evaluated using Balb/c mouse liver S9 fractions: The reaction mixtures consisted of liver S9 fraction (final protein concentration: 1 mg/mL) in 100 mM potassium phosphate buffer (pH 7.4). Two parallel conditions were prepared: one containing an NADPH-generating system (NGS) and the other without NGS. Both mixtures were pre-incubated at 37°C for 5 min. The metabolic reaction was initiated by spiking the test compound into the mixtures to achieve a final concentration of 1 µM. The incubation was performed at 37°C in a shaking water bath. At predetermined time points (0, 5, 10, 25, 40, and 60 min), aliquots were collected and immediately quenched with ice-cold acetonitrile. The samples were centrifuged, and the supernatants were analyzed by LC-MS. The intrinsic clearance ($CL_{int,\textit{in vitro}}$) and half-life ($t_{1/2}$) were calculated using the slope of the natural log of the percentage remaining versus time.
In vitro stability in plasma: Balb/c mouse plasma was pre-incubated at 37°C for 5 min. The reaction was initiated by spiking thiogochangamide B (final concentration: 1 µM) into the plasma. The mixture was incubated at 37°C in a shaking water bath. Samples were collected at 0, 10, 25, 40, 60, 90, and 120 min and immediately quenched with ice-cold acetonitrile. After centrifugation, the supernatant was analyzed by LC-MS to determine the percentage of the compound remaining over time.

MS spectra

Figure S1. Mass spectrum of a sulfur-bearing compound detected in the extract of *Streptomyces* sp. GC2. (a) experimental spectrum, (b) overlay with predicted isotope pattern (red) for a molecular formula candidate ($C_{56}H_{87}N_{14}O_{10}S_6$) and measured data (black).

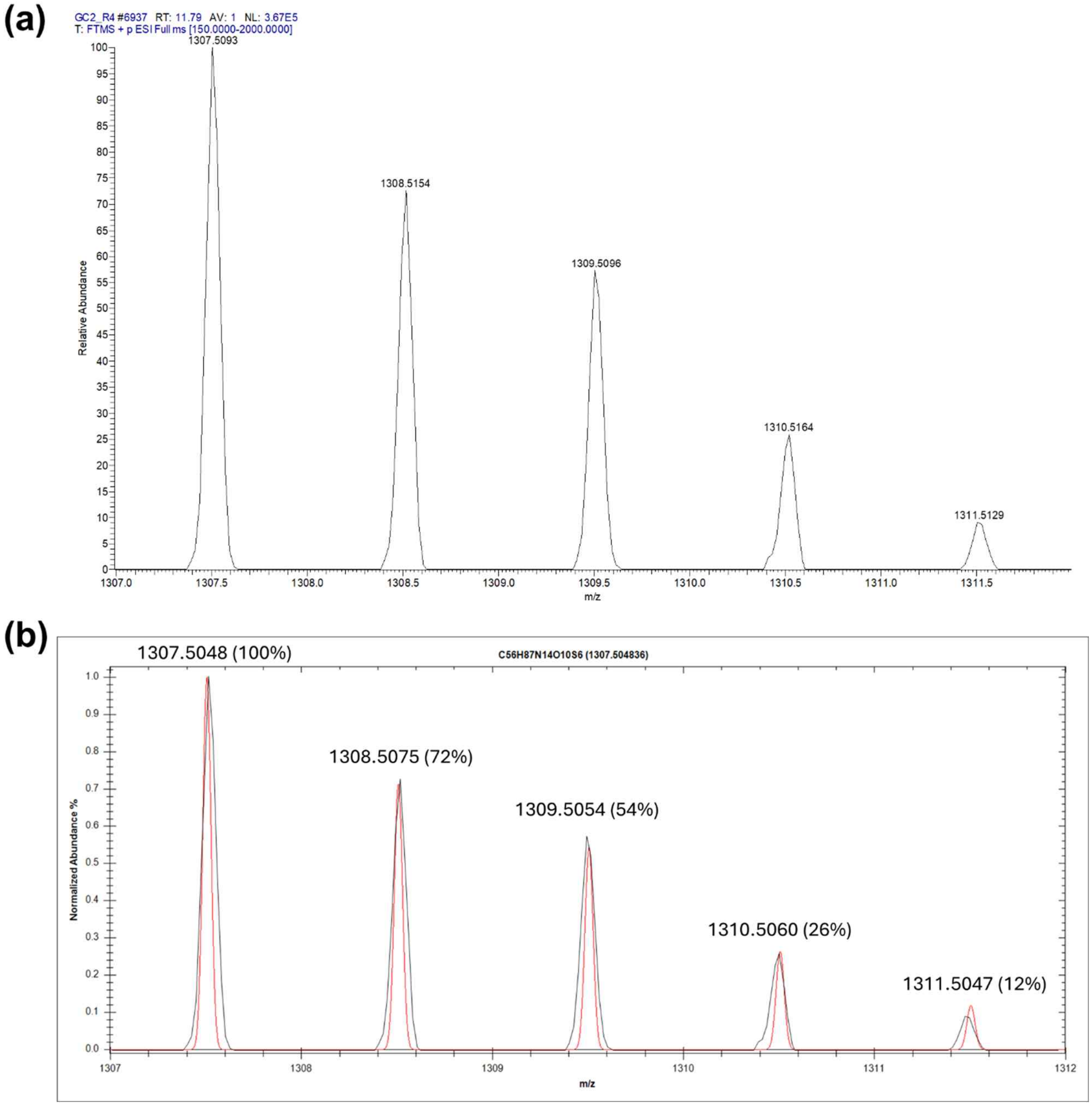

Figure S2. HR-MS spectra of thiogochangamides A (**1**) and B (**2**).

Thiogochangamide A (**1**)

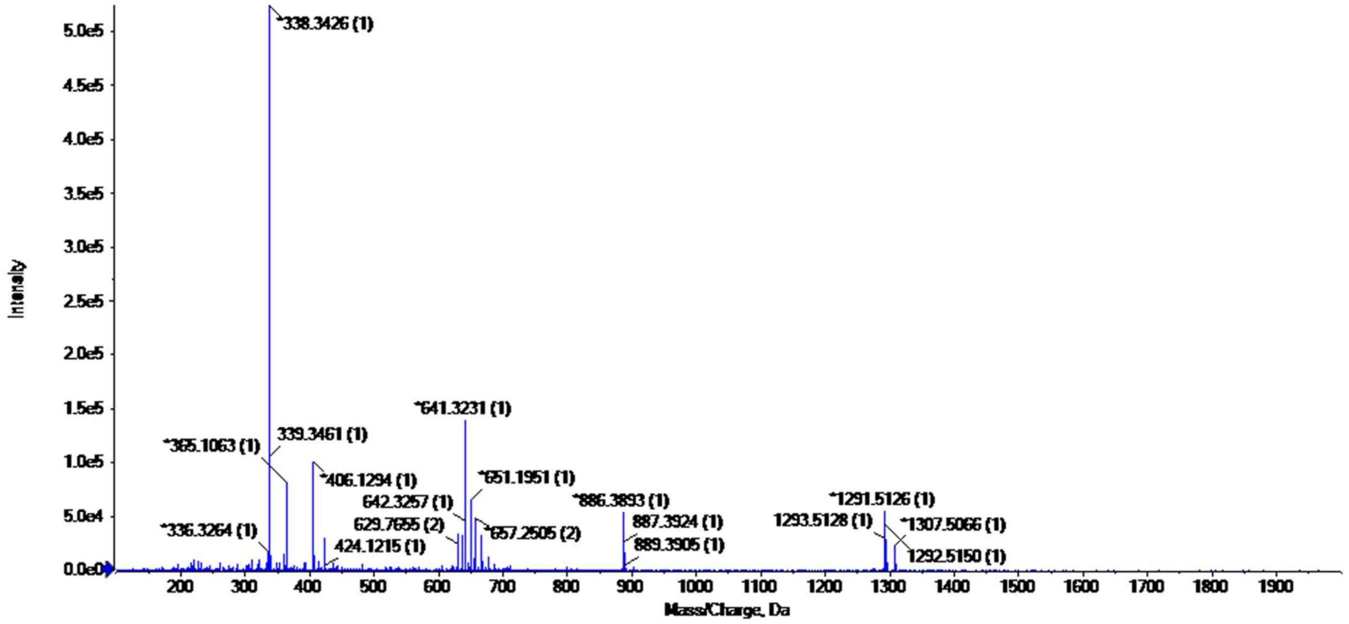

Thiogochangamide B (**2**)

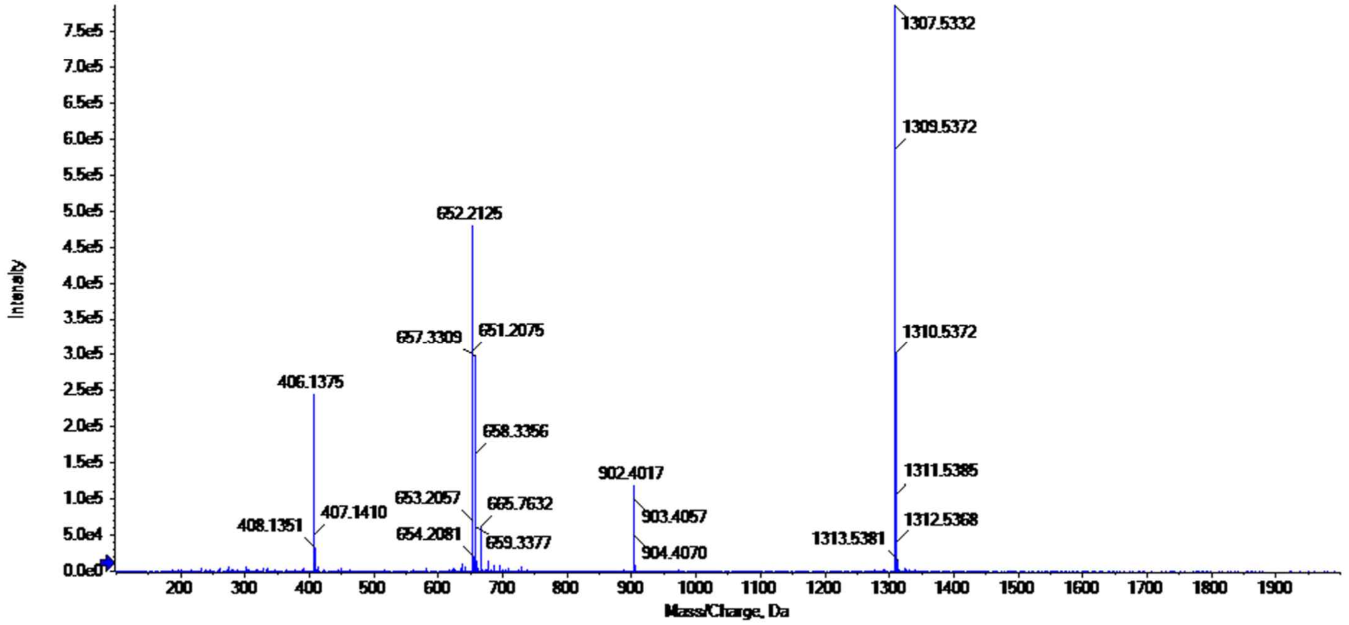

NMR spectra

Figure S3. ^1H NMR spectrum of oxidized-thiostreptamide S4 at 900 MHz in DMSO-d_6.

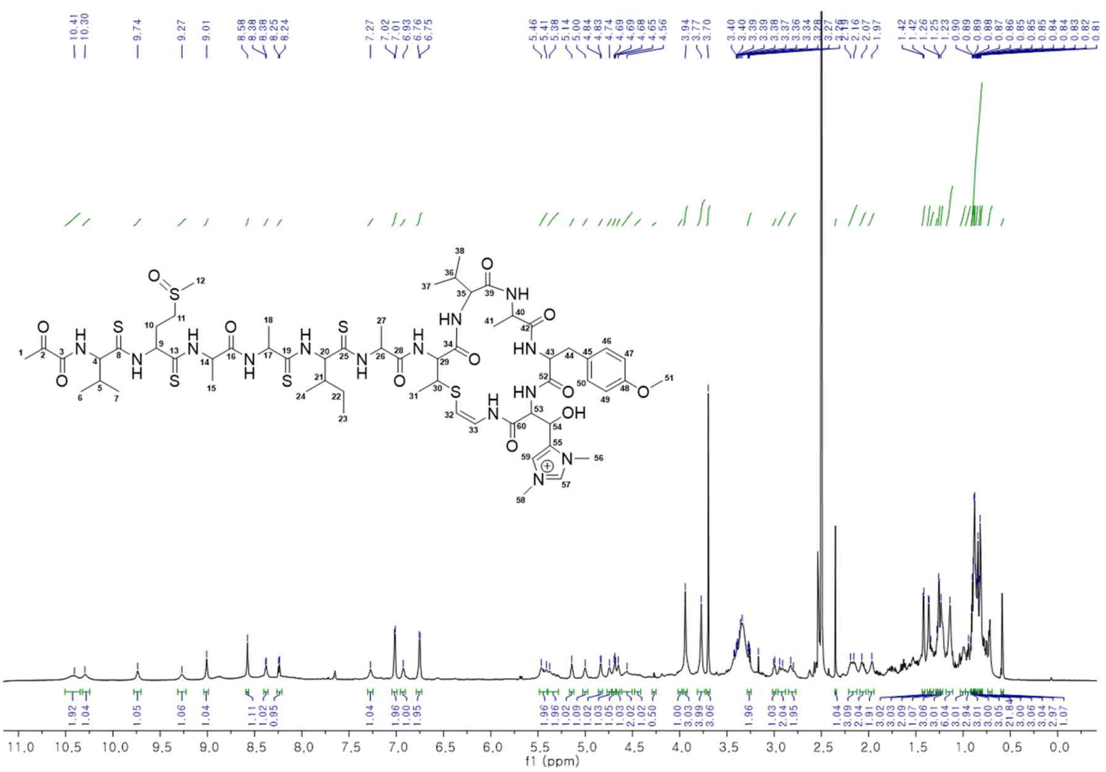

Figure S4. ^{13}C NMR spectrum of oxidized-thiostreptamide S4 at 225 MHz in DMSO-d_6.

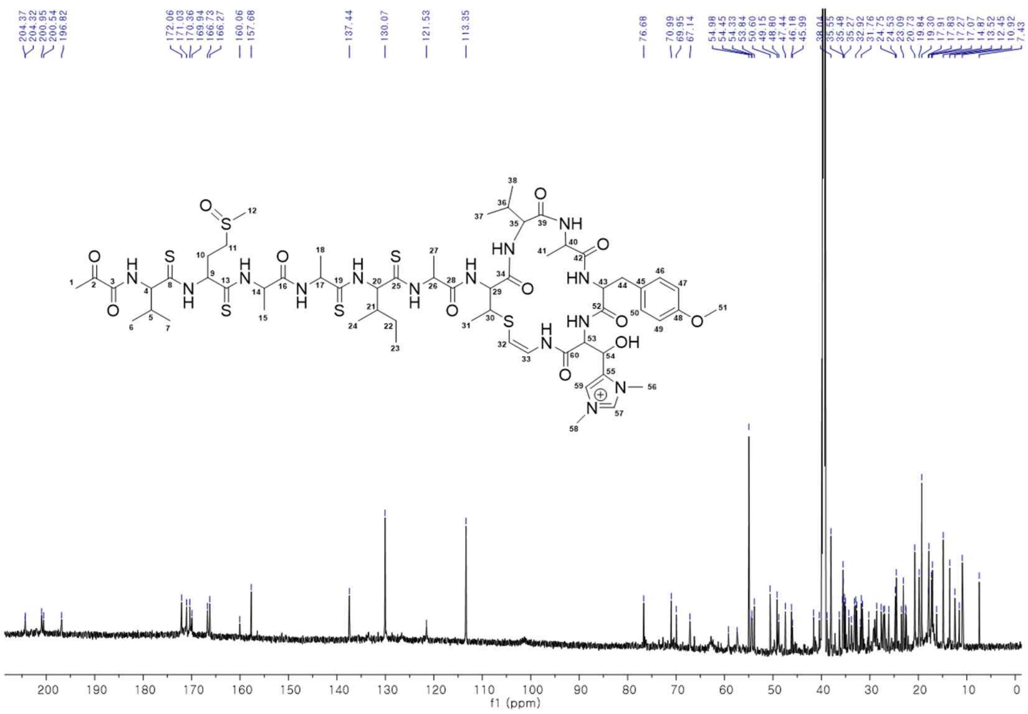

Figure S5. HSQC spectrum of oxidized-thiostreptamide S4 at 900 MHz in DMSO-d_6.

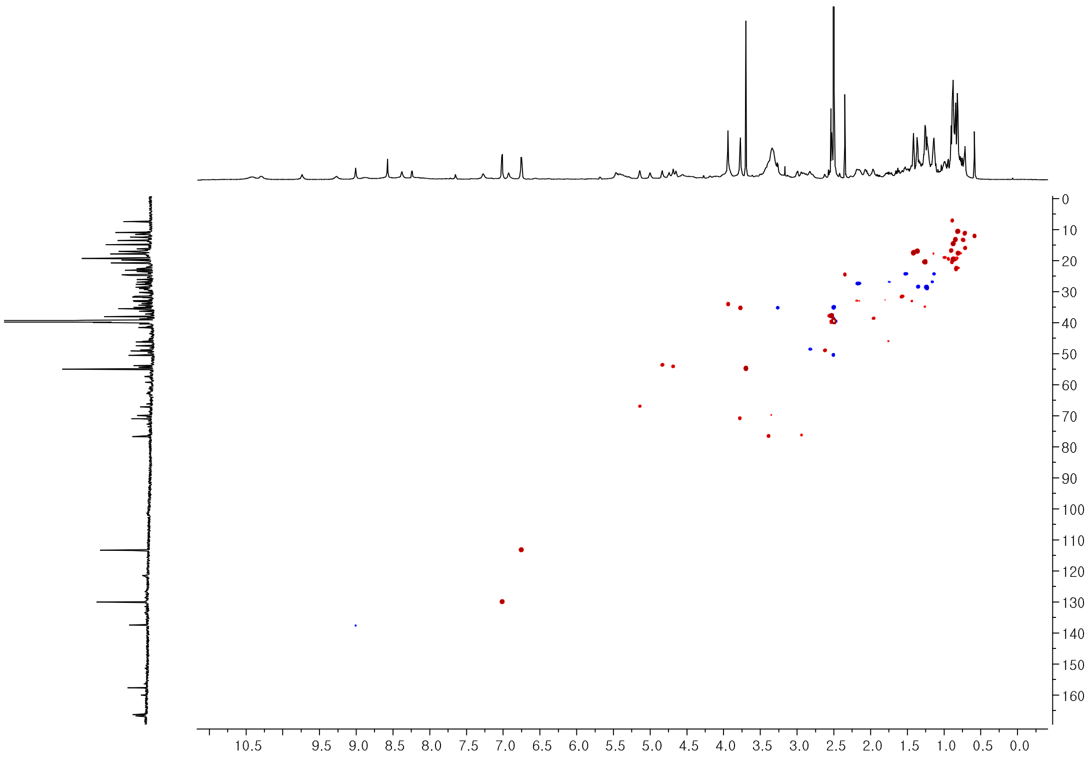

Figure S6. COSY spectrum of oxidized-thiostreptamide S4 at 900 MHz in DMSO-d_6.

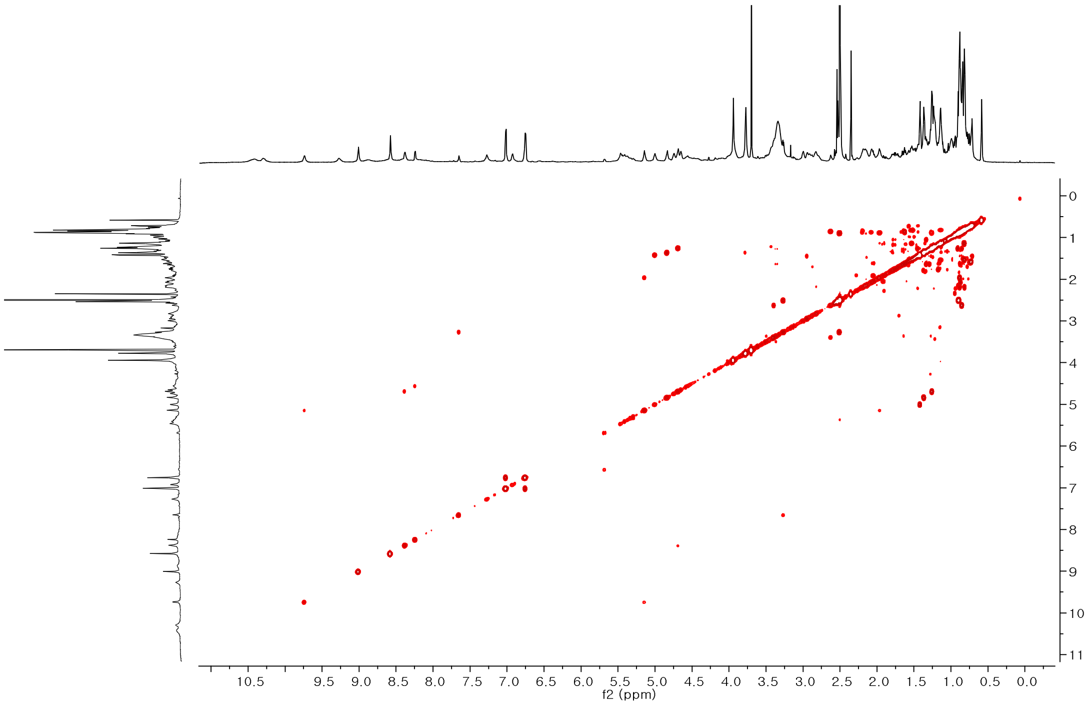

Figure S7. HMBC spectrum of oxidized-thiostreptamide S4 at 900 MHz in DMSO-d_6.

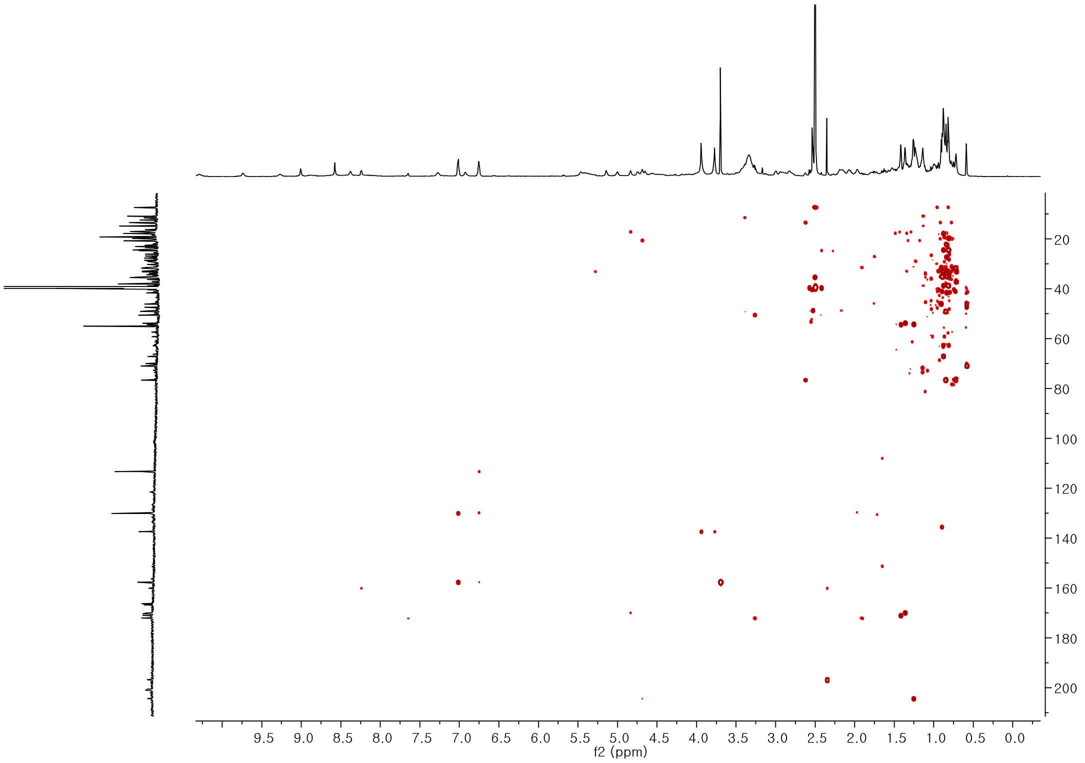

Figure S8. TOCSY spectrum of oxidized-thiostreptamide S4 at 900 MHz in DMSO-d_6.

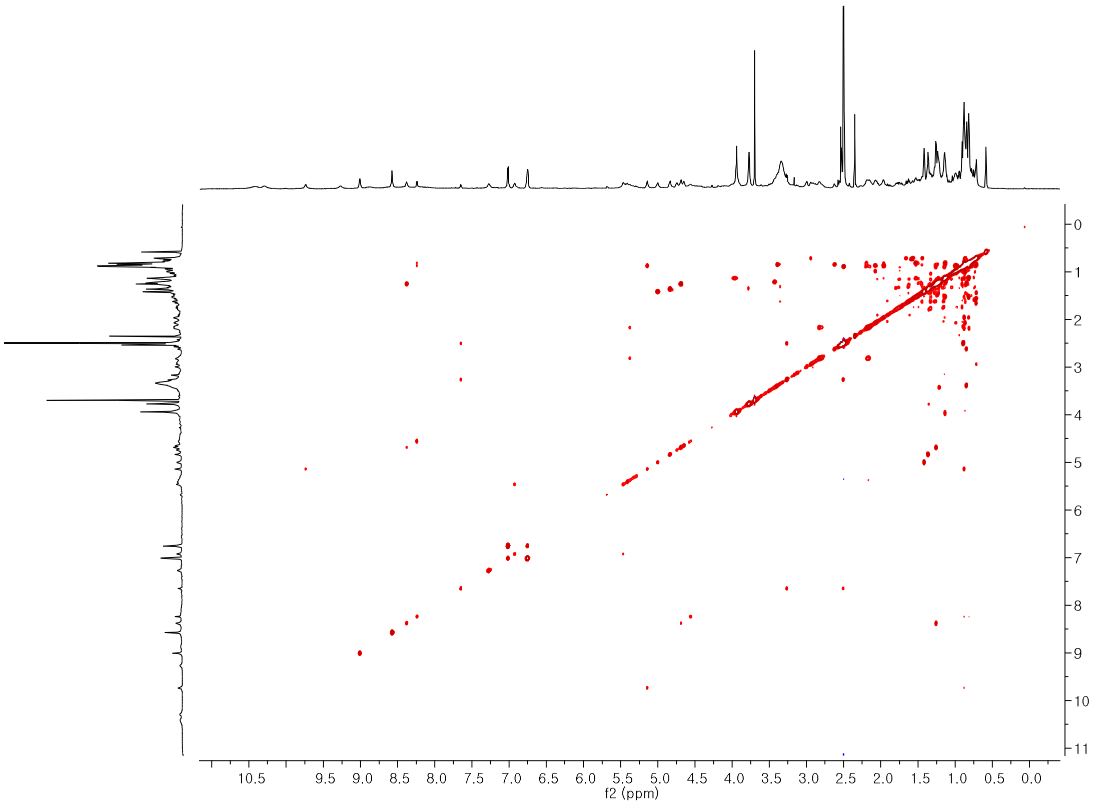

Figure S9. ROESY spectrum of oxidized-thiostreptamide S4 at 900 MHz in DMSO-d_6.

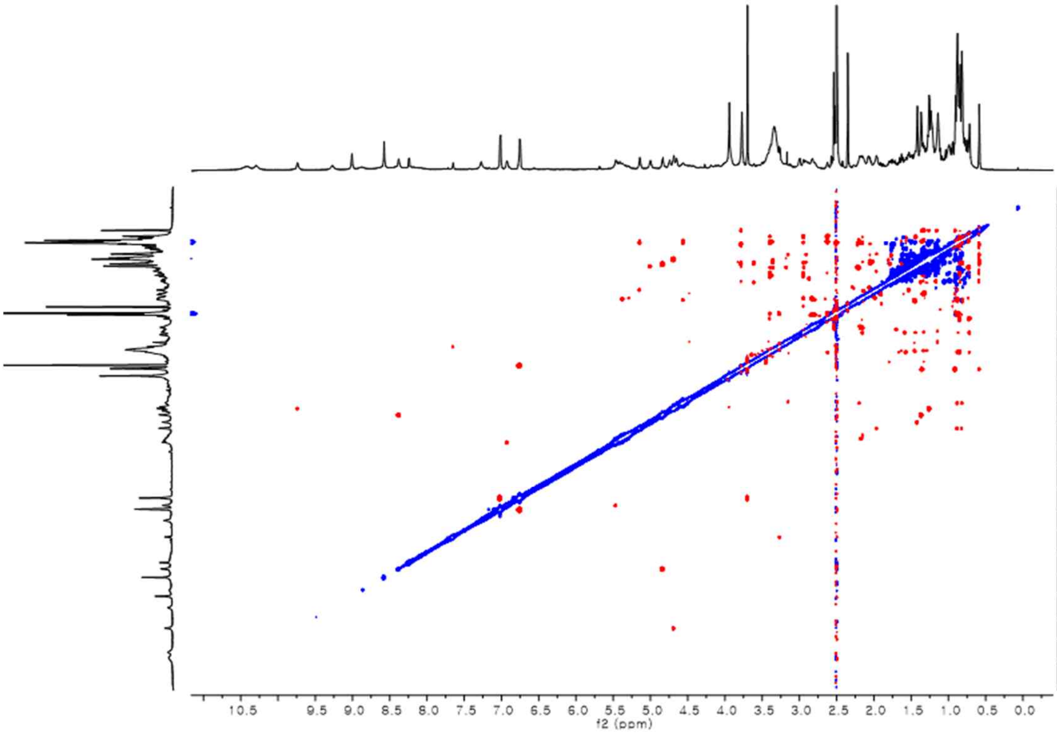

Figure S10. ^{1}H NMR spectrum of thiogochangamide A (**1**) at 800 MHz in acetonitrile-d_3.

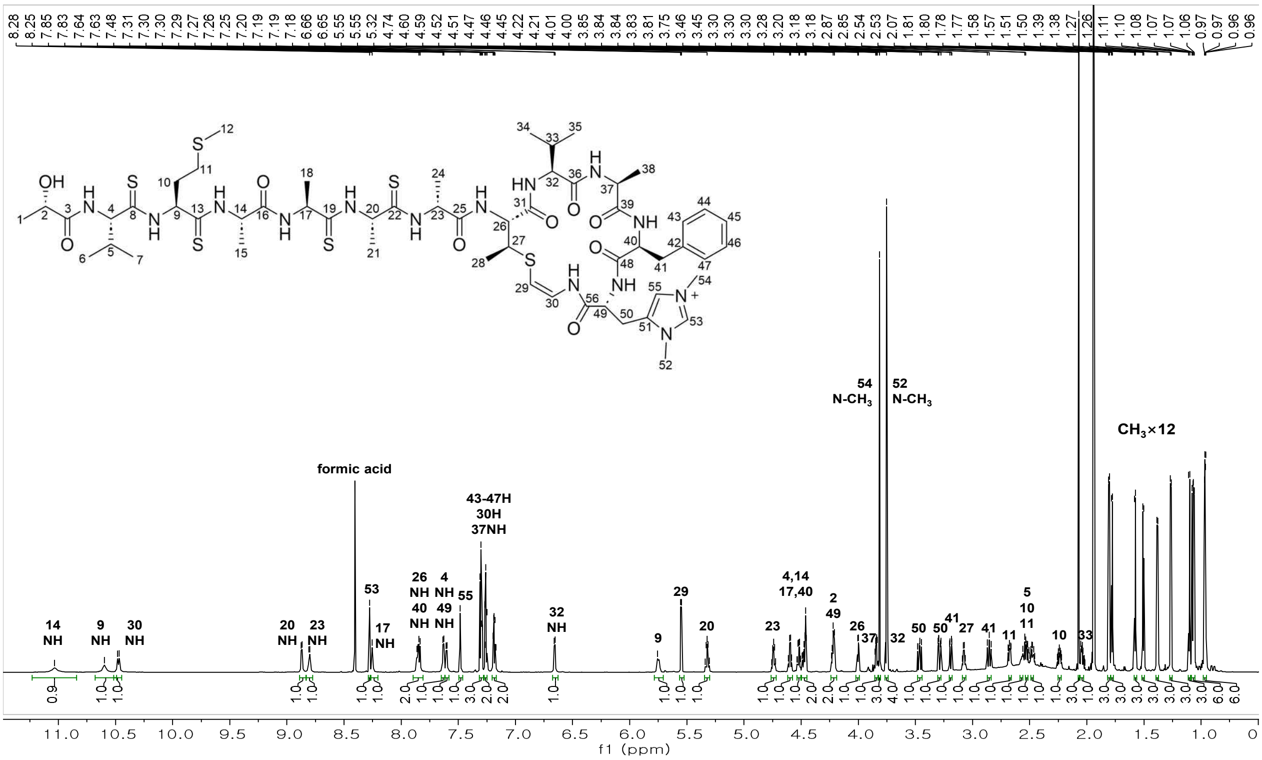

Figure S11. ^{13}C NMR spectrum of thiogochangamide A (**1**) at 200 MHz in acetonitrile-d_3

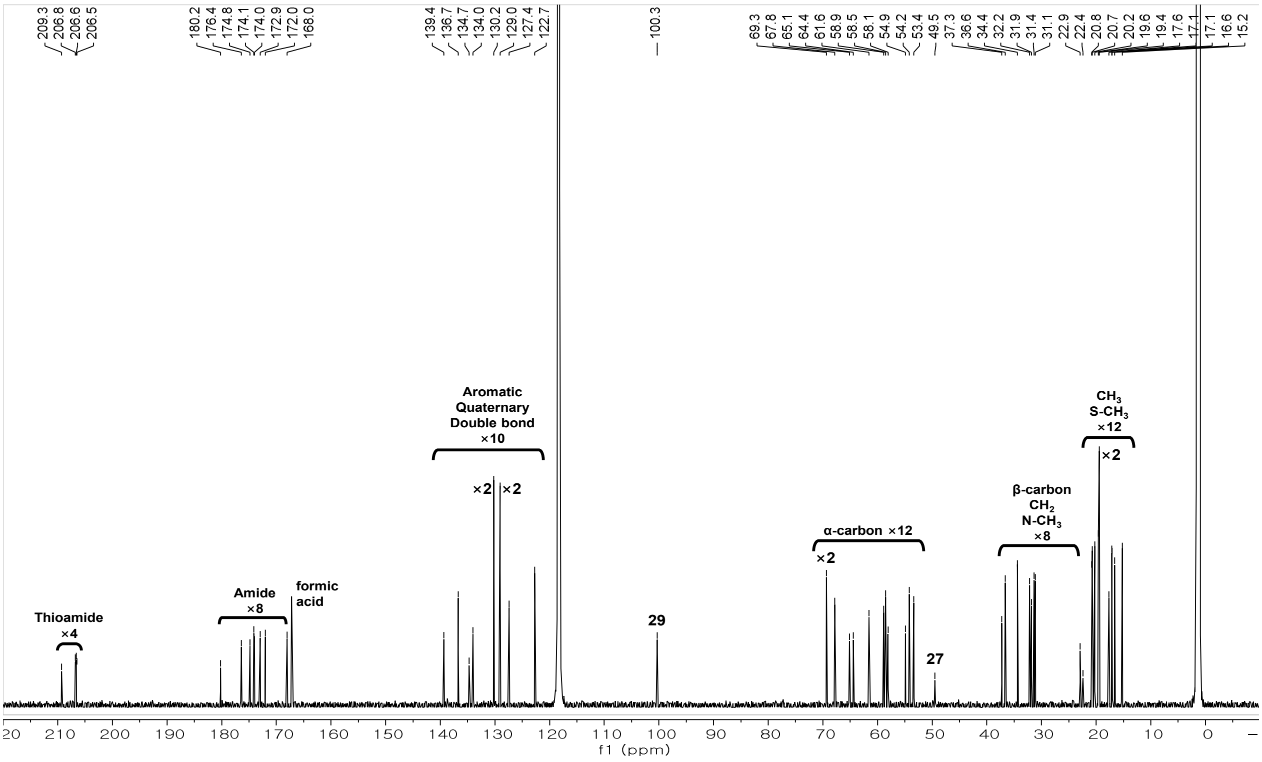

Figure S12. HSQC NMR spectrum of thiogochangamide A (**1**) at 800 MHz in acetonitrile-d_3

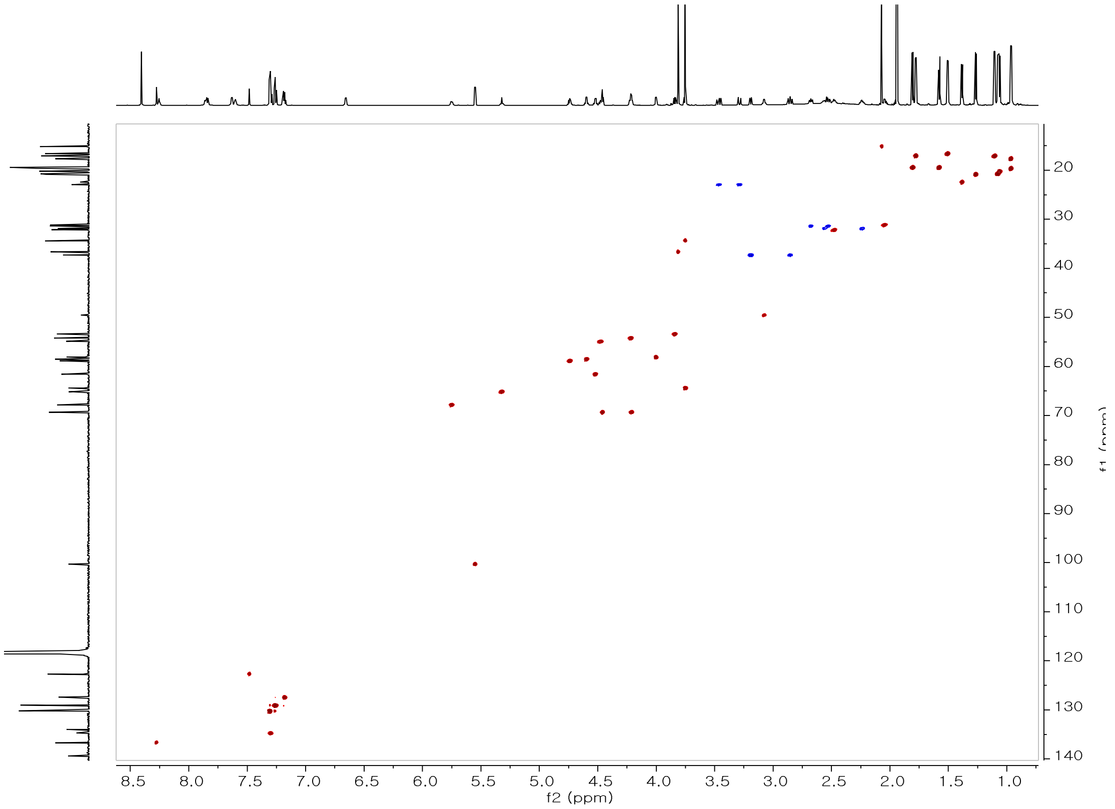

Figure S13. COSY NMR spectrum of thiogochangamide A (**1**) at 800 MHz in acetonitrile-d_3.

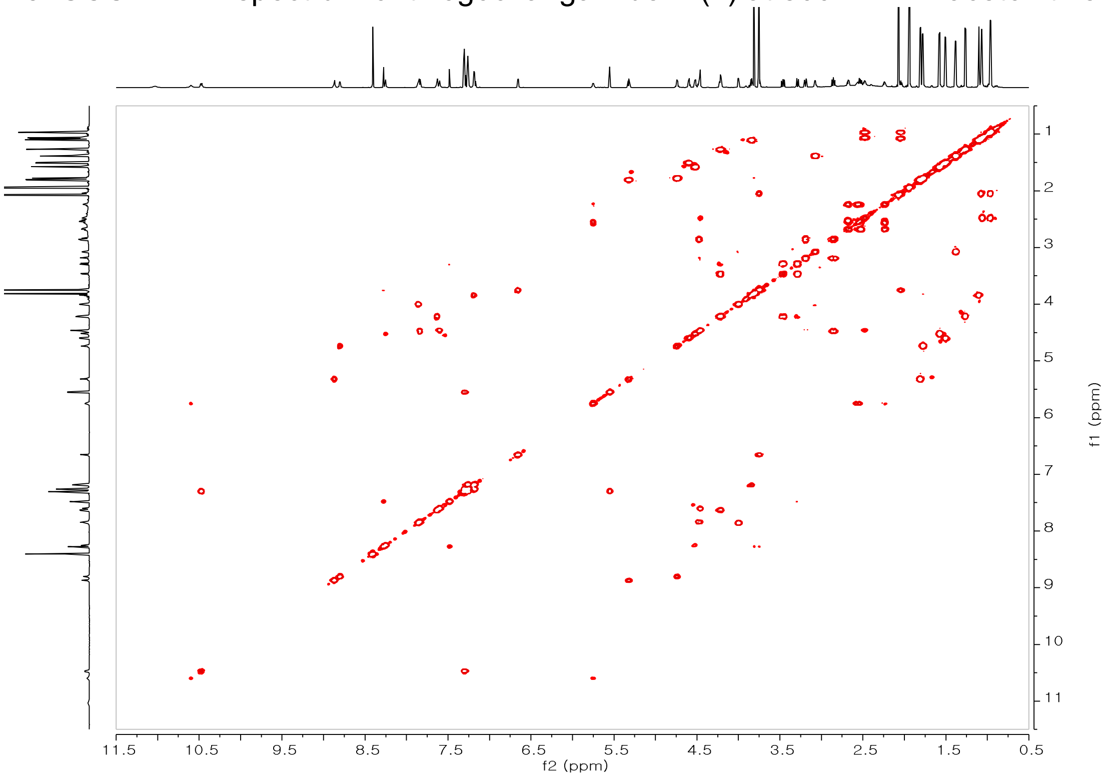

Figure S14. HMBC NMR spectrum of thiogochangamide A (**1**) at 800 MHz in acetonitrile-d_3.

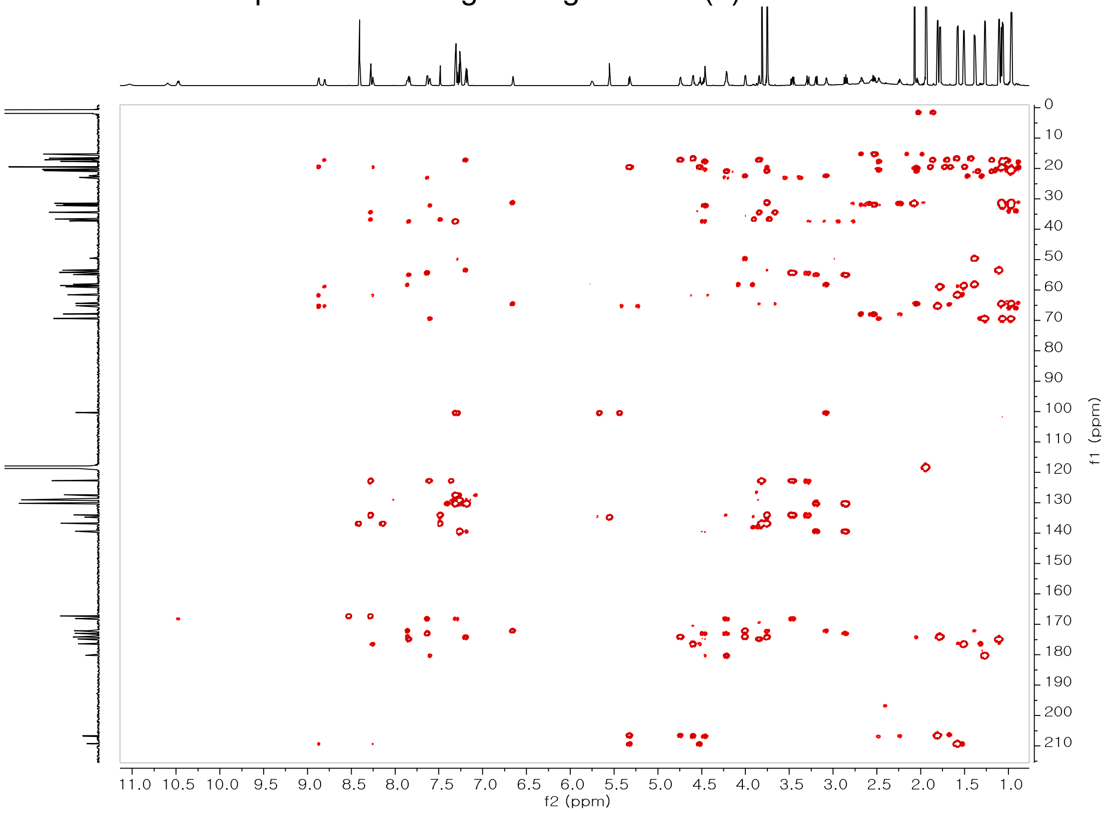

Figure S15. ROESY NMR spectrum of thiogochangamide A (**1**) at 800 MHz in acetonitrile-d_3.

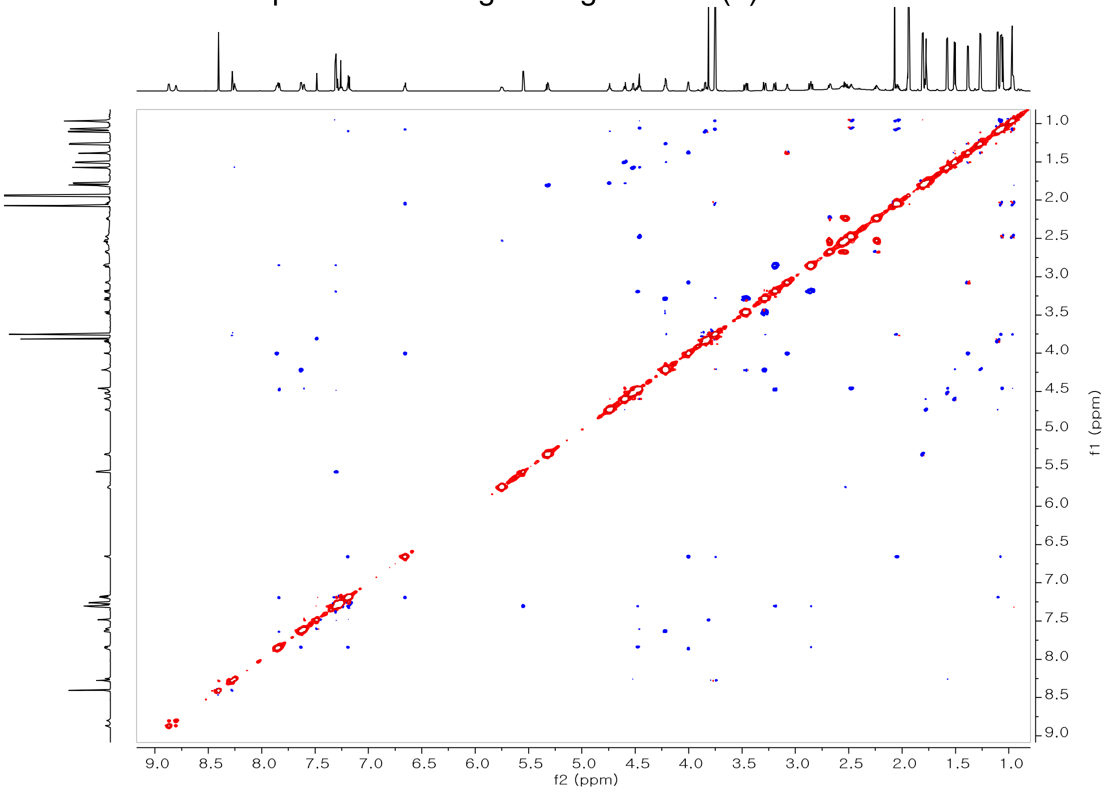

Figure S16. TOCSY NMR spectrum of thiogochangamide A (**1**) at 800 MHz in acetonitrile-d_3.

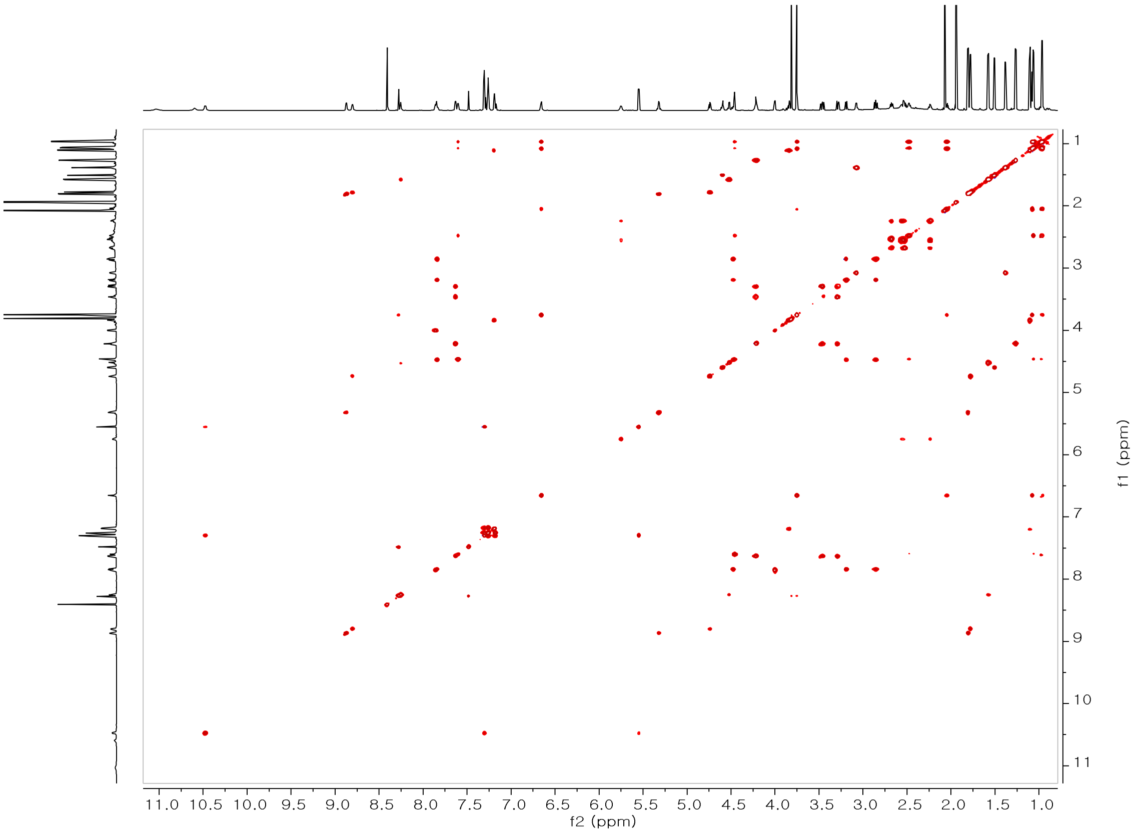

Figure S17.^{1}H-^{15}N HSQC NMR spectrum of thiogochangamide A (**1**) at 850 MHz in acetonitrile-d_3.

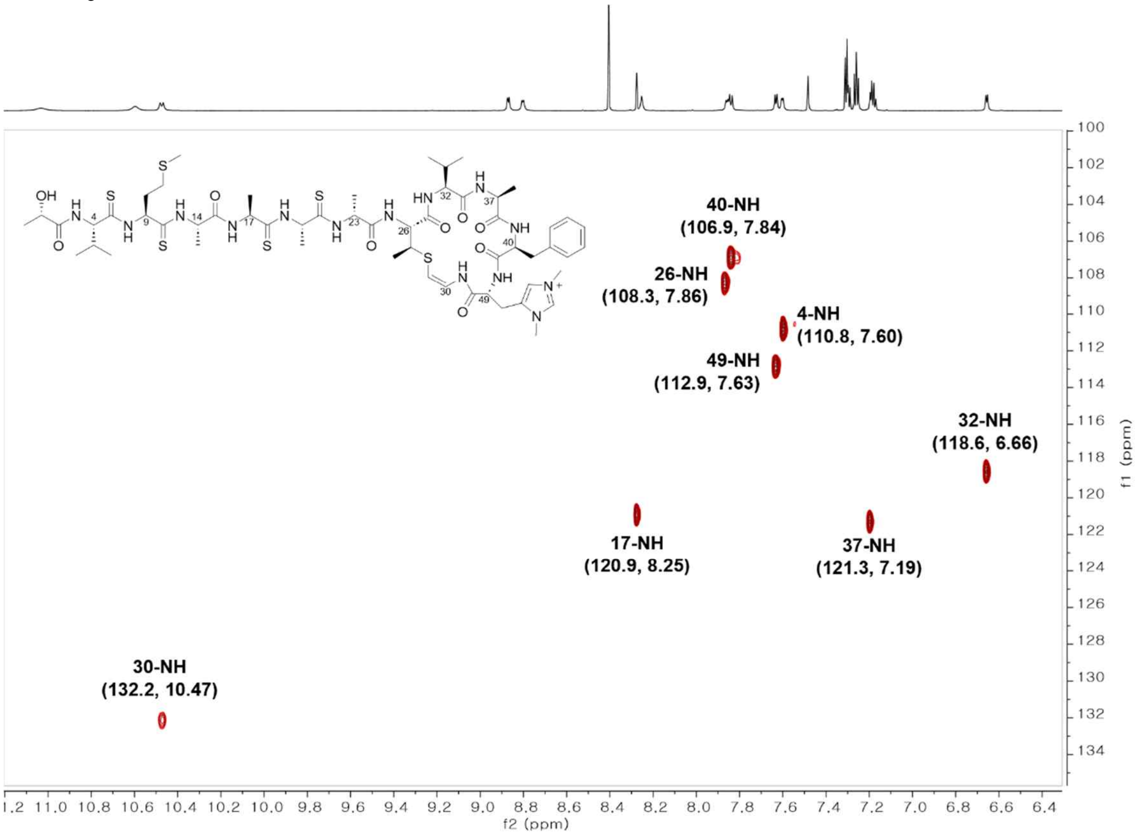

Figure S18. ^1H-^{15}N HMBC NMR spectrum of thiogochangamide A (**1**) at 850 MHz in acetonitrile-d_3.

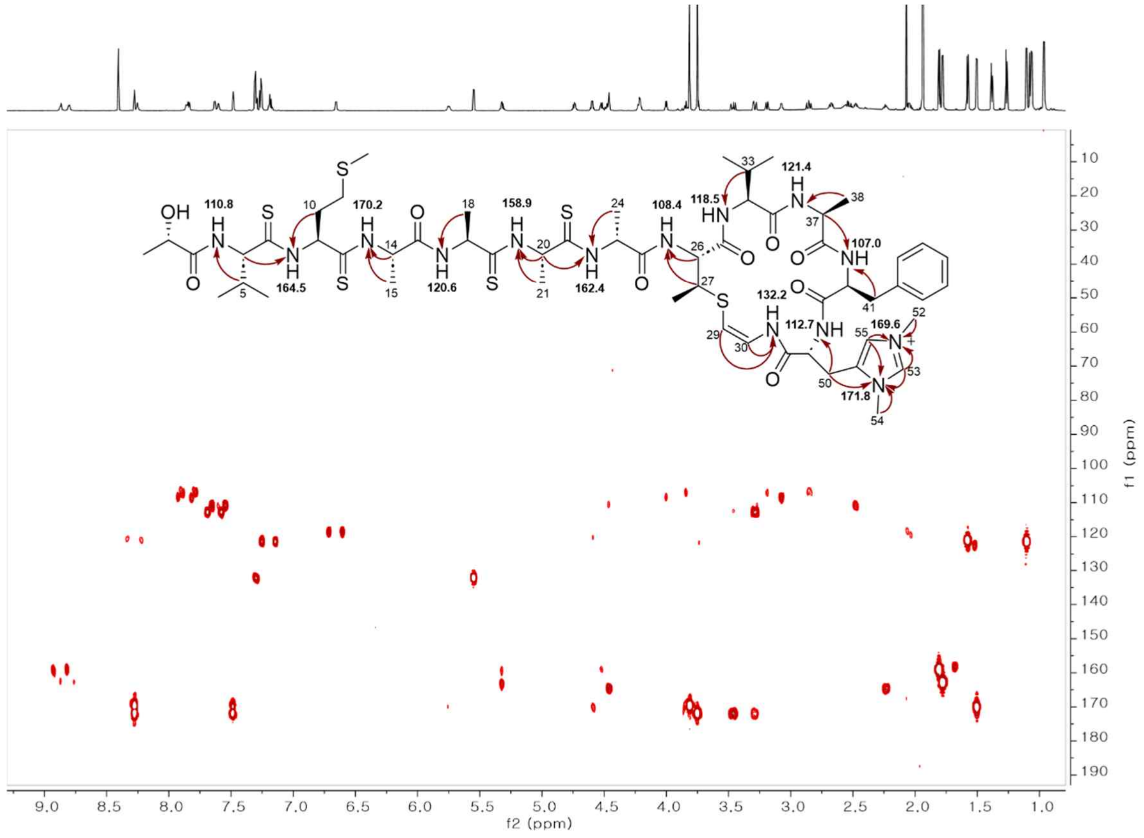

Figure S19. ^1H NMR spectrum of thiogochangamide B (**2**) at 800 MHz in acetonitrile-d_3.

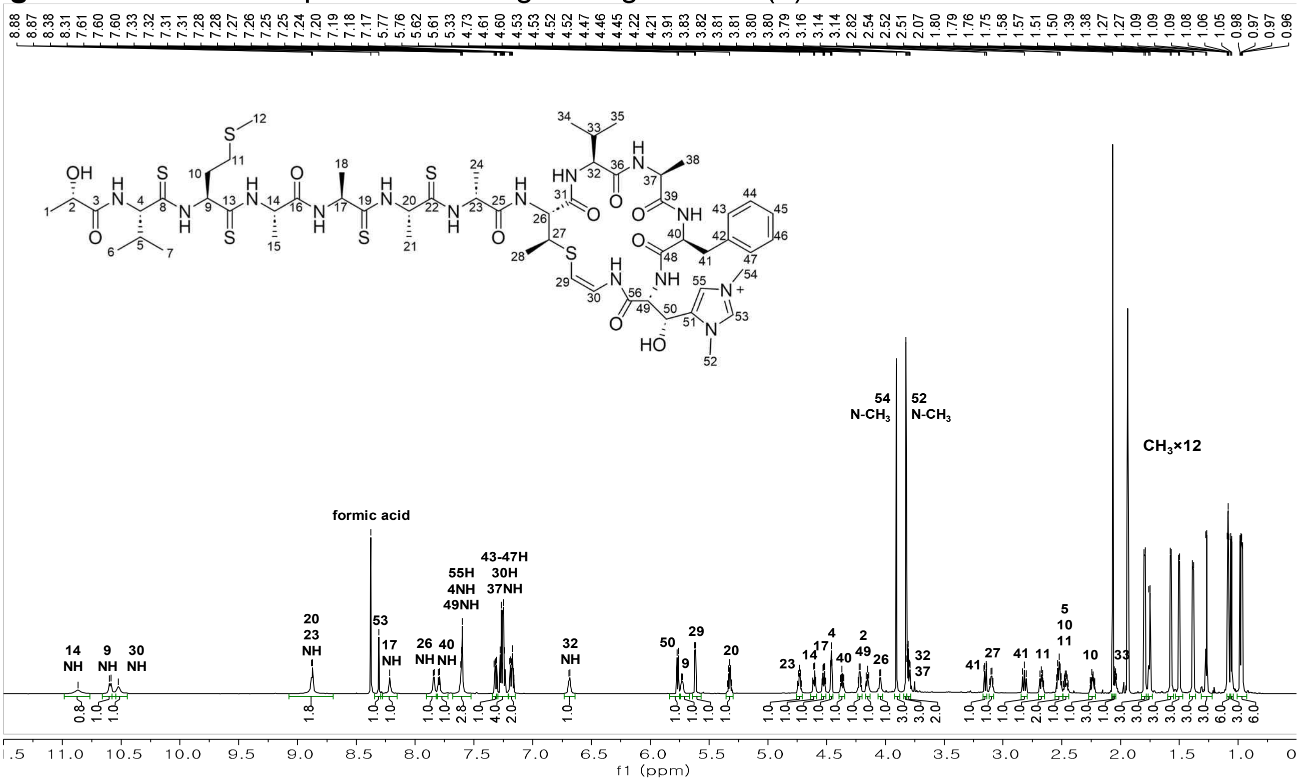

Figure S20. ^{13}C NMR spectrum of thiogochangamide B (**2**) at 800 MHz in acetonitrile-d_3.

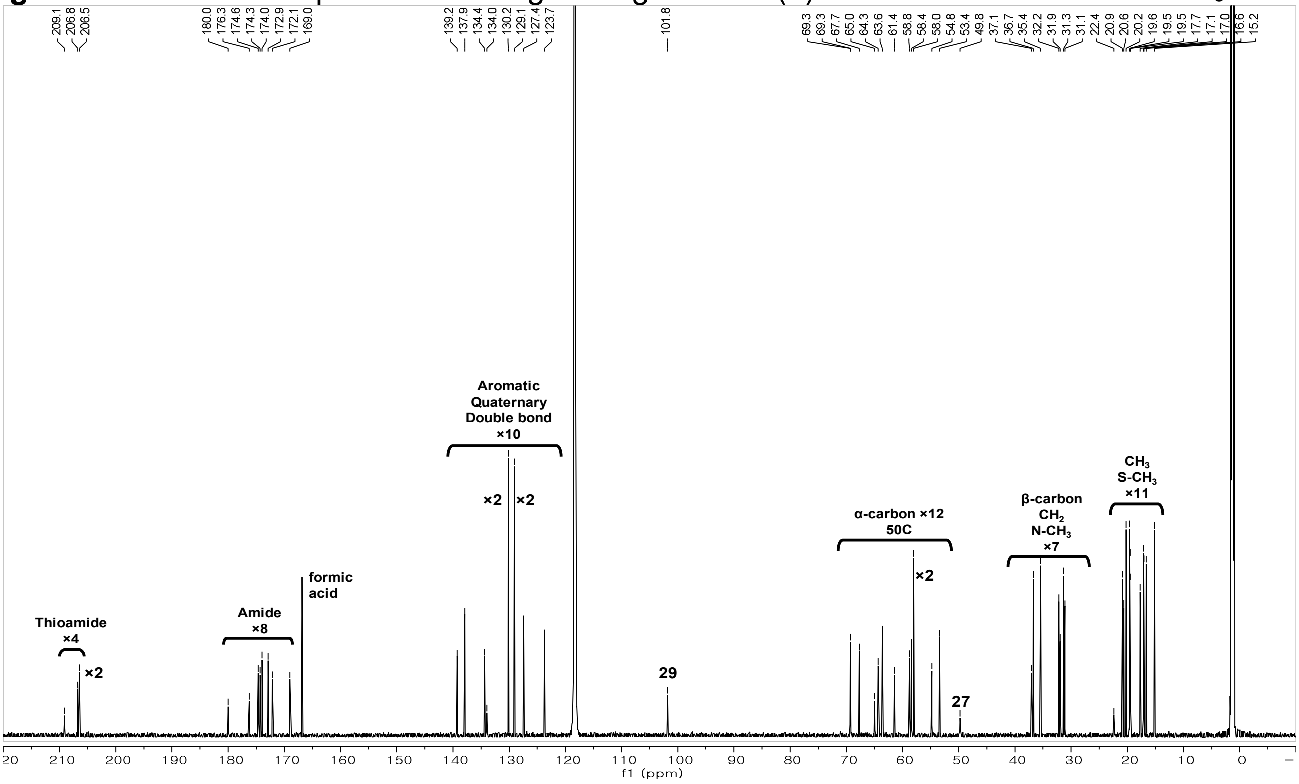

Figure S21. HSQC NMR spectrum of thiogochangamide B (**2**) at 800 MHz in acetonitrile-d_3.

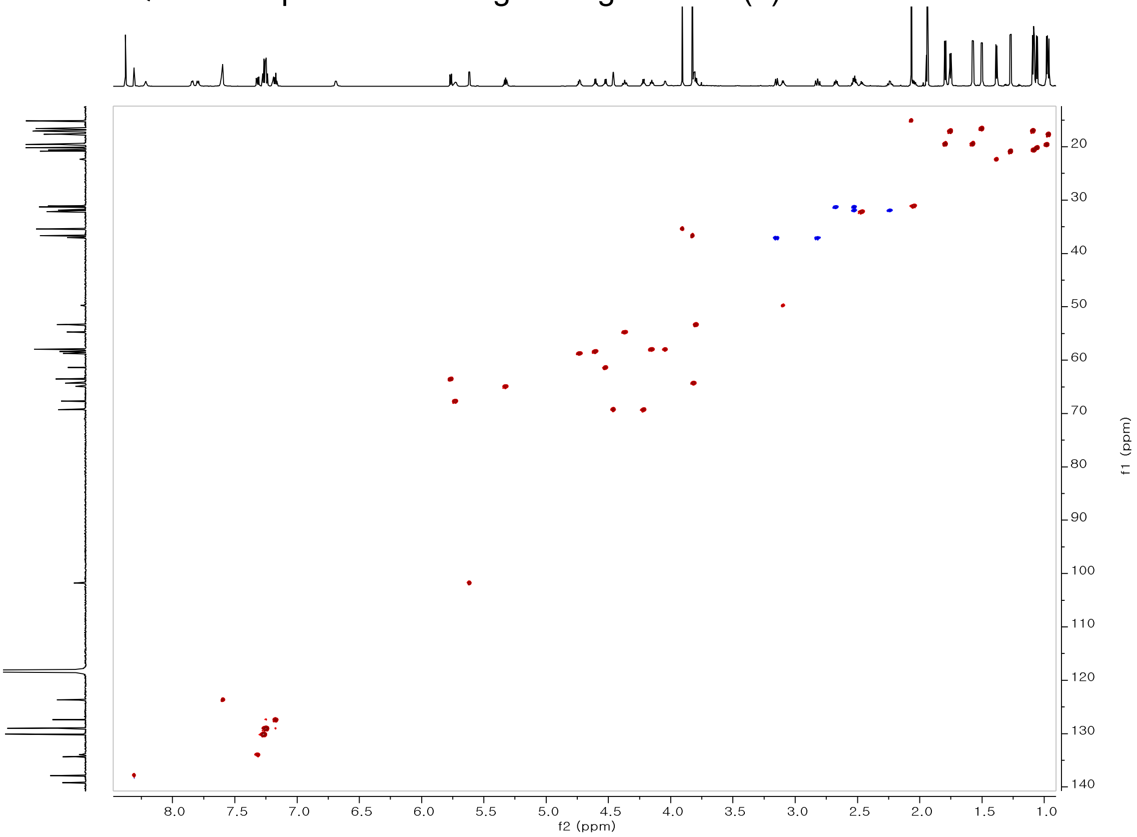

Figure S22. COSY NMR spectrum of thiogochangamide B (**2**) at 800 MHz in acetonitrile-d_3.

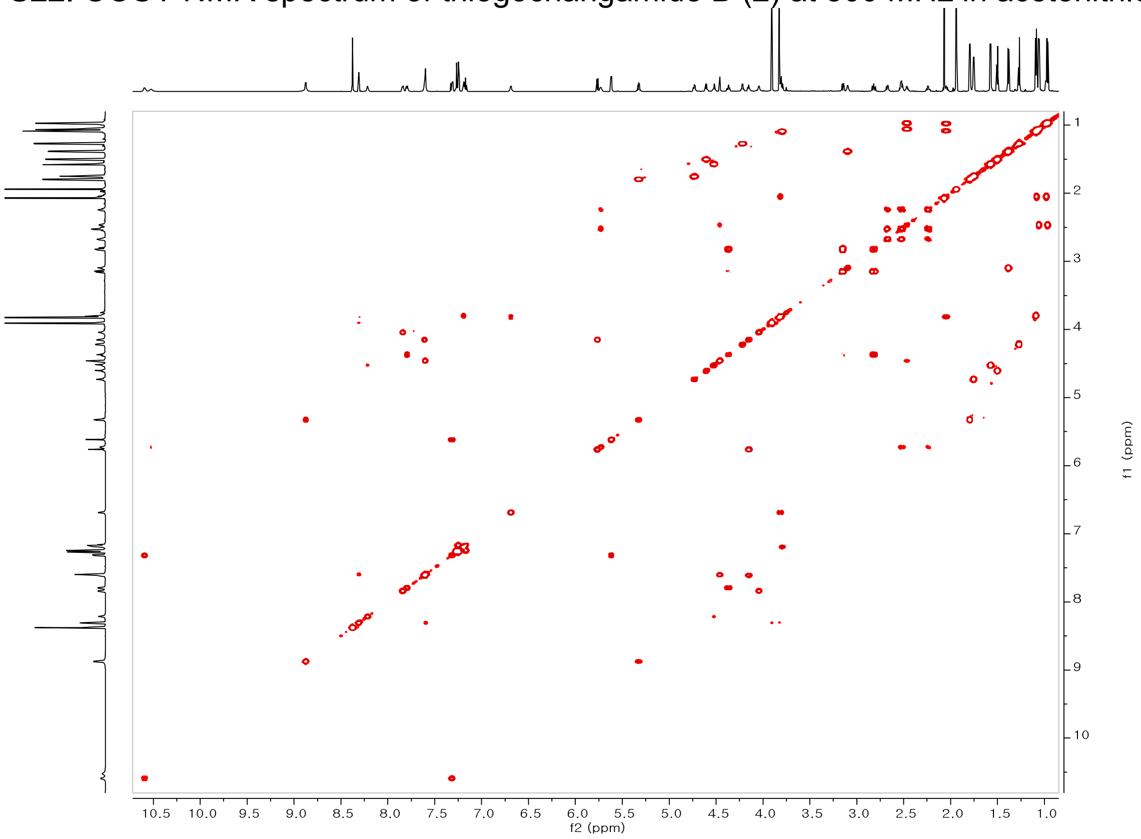

Figure S23. HMBC NMR spectrum of thiogochangamide B (**2**) at 800 MHz in acetonitrile-d_3.

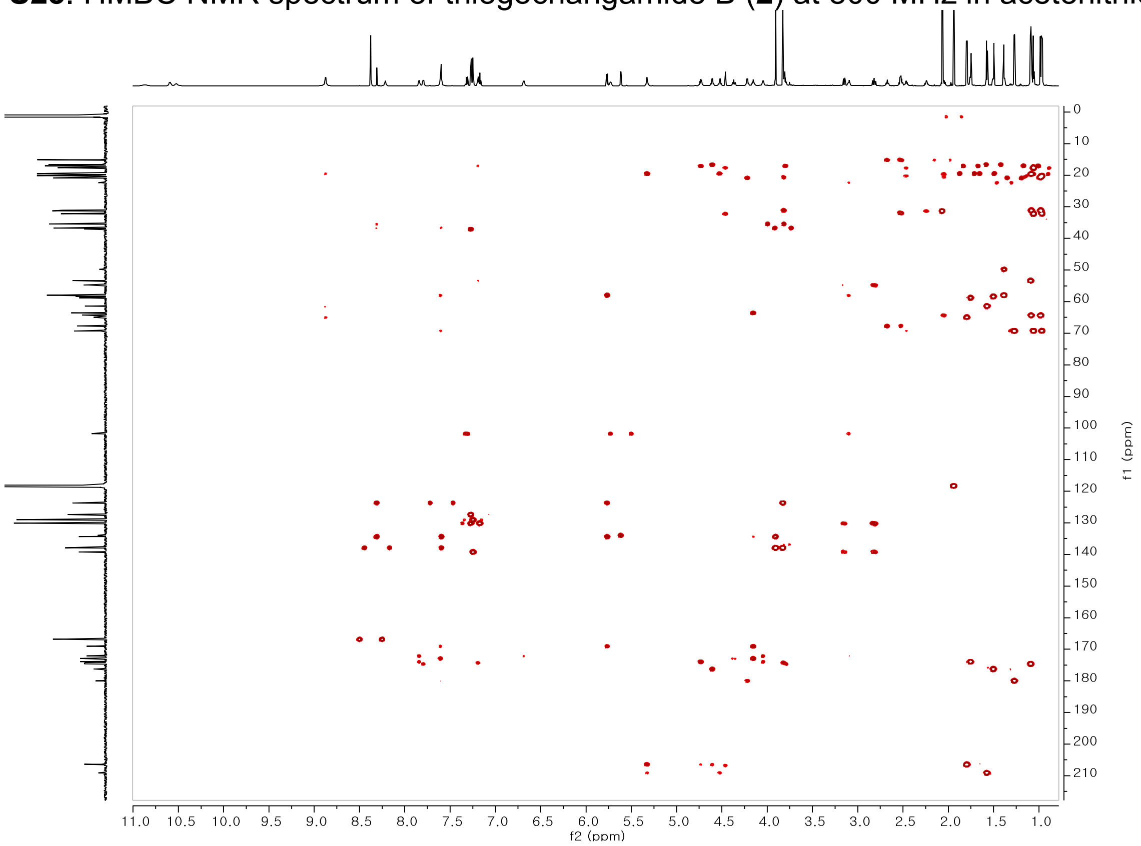

Figure S24. ROESY NMR spectrum of thiogochangamide B (**2**) at 800 MHz in acetonitrile-d_3.

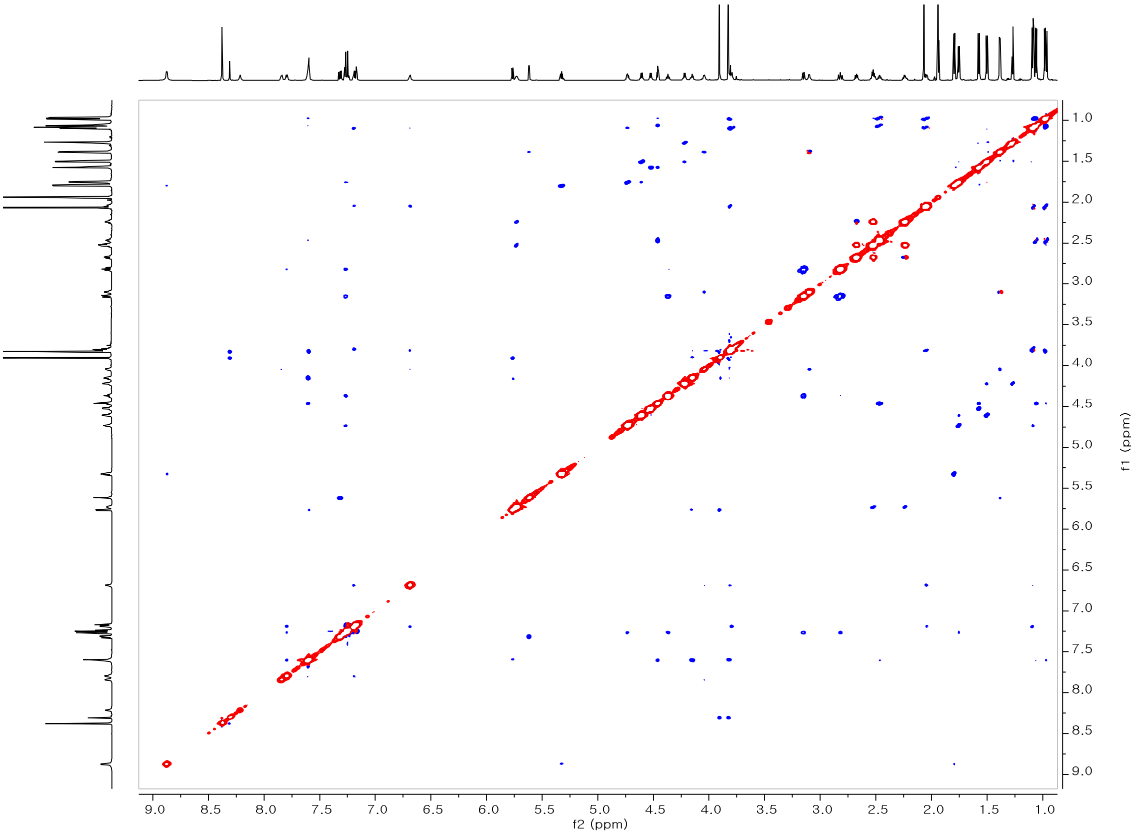

Figure S25. TOCSY NMR spectrum of thiogochangamide B (**2**) at 800 MHz in acetonitrile-d_3

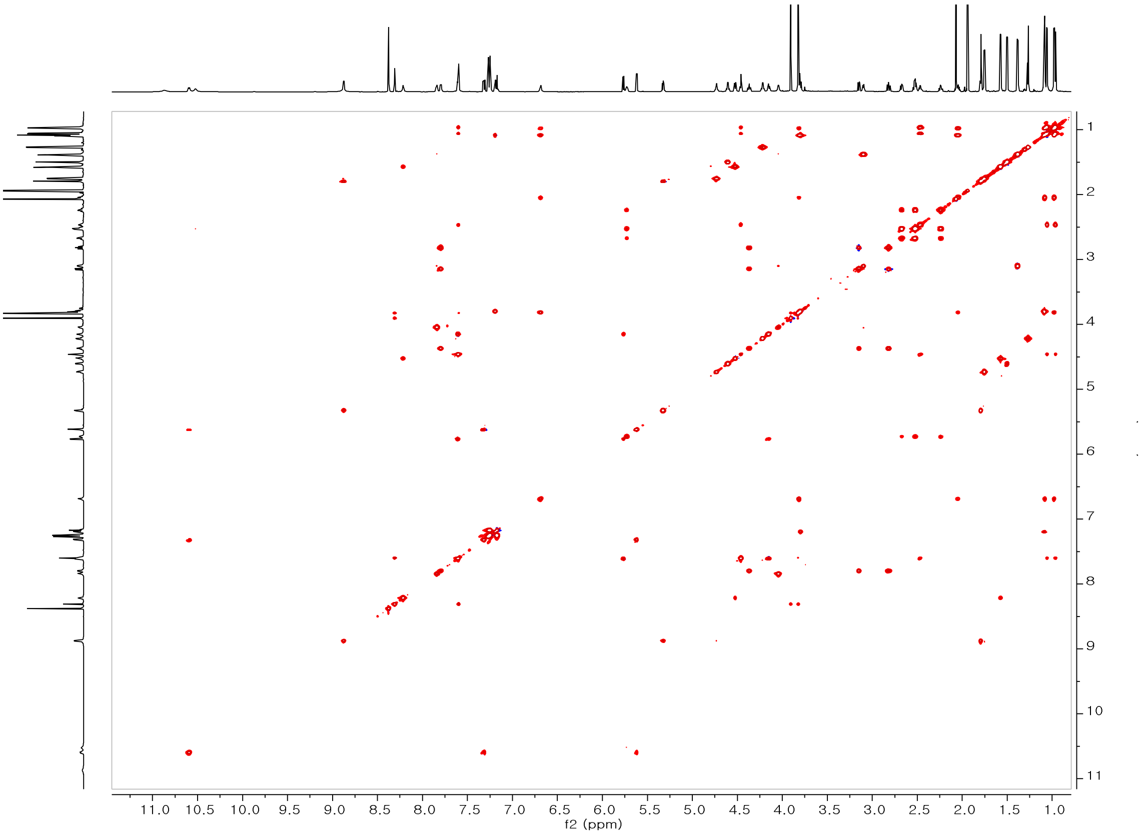

Figure S26. ^1H NMR spectrum of *S*-MTPA ester of thiogochangamide B at 800 MHz in acetonitrile-d_3.

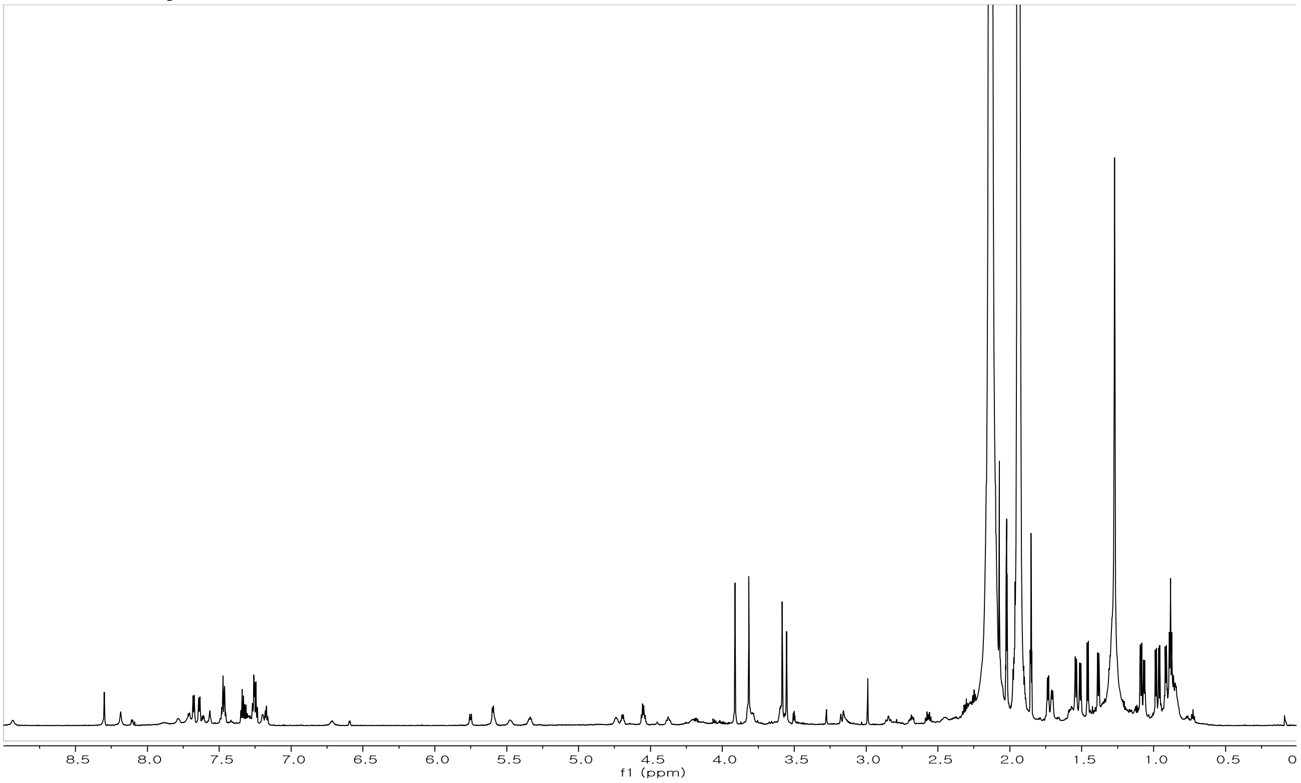

Figure S27. HSQC NMR spectrum of *S*-MTPA ester of thiogochangamide B at 800 MHz in acetonitrile-d_3.

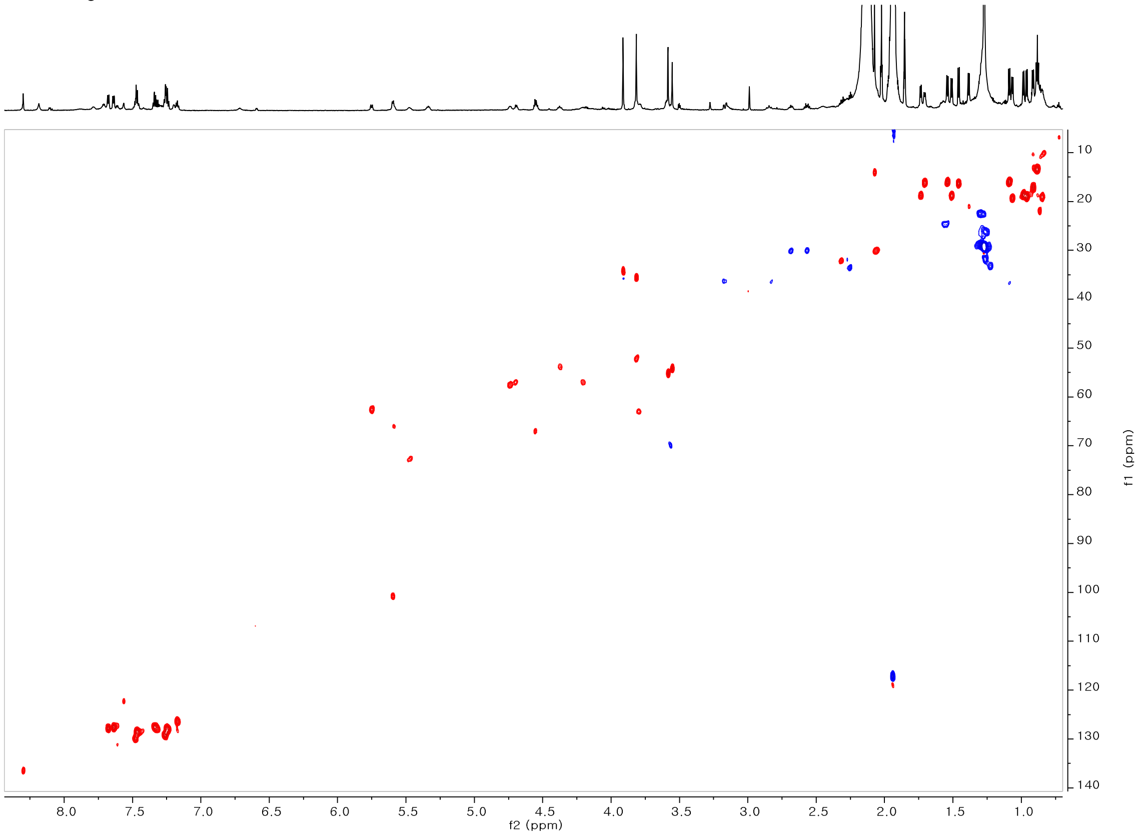

Figure S28. COSY NMR spectrum of *S*-MTPA ester of thiogochangamide B at 800 MHz in acetonitrile-d_3.

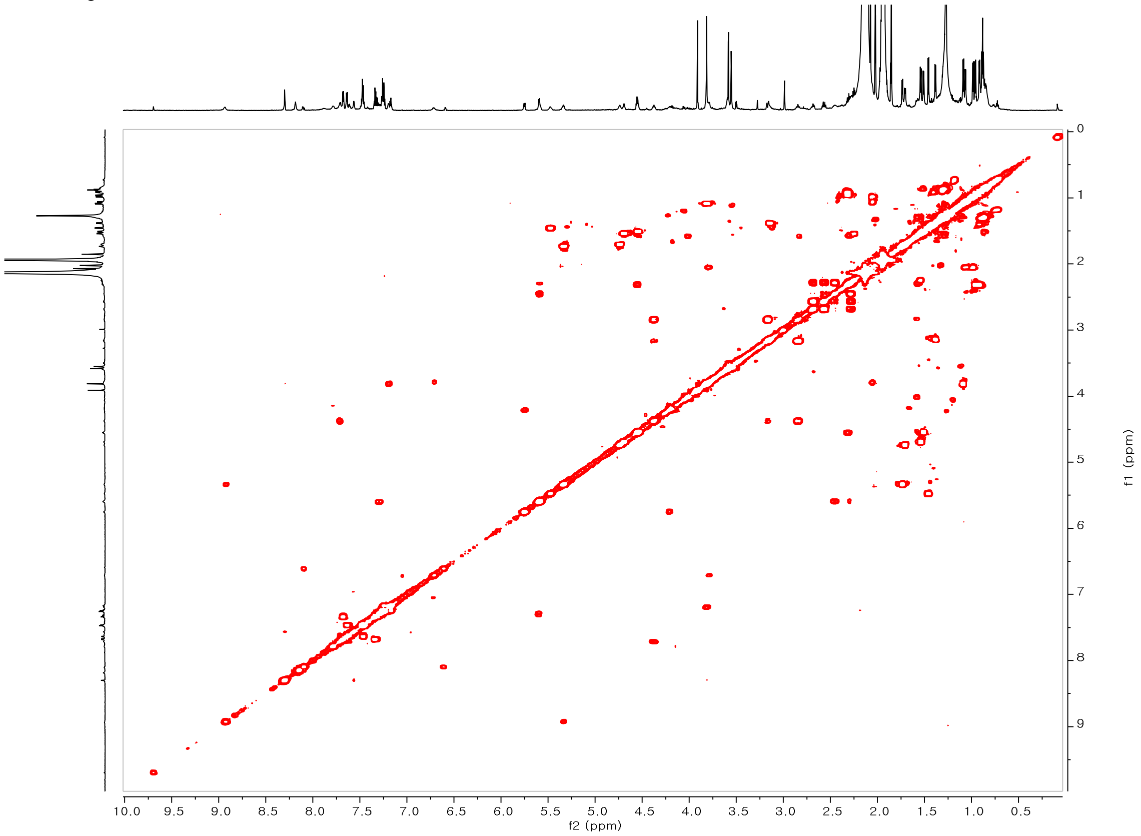

Figure S29. ^{1}H NMR spectrum of *R*-MTPA ester of thiogochangamide B at 800 MHz in acetonitrile-d_3.

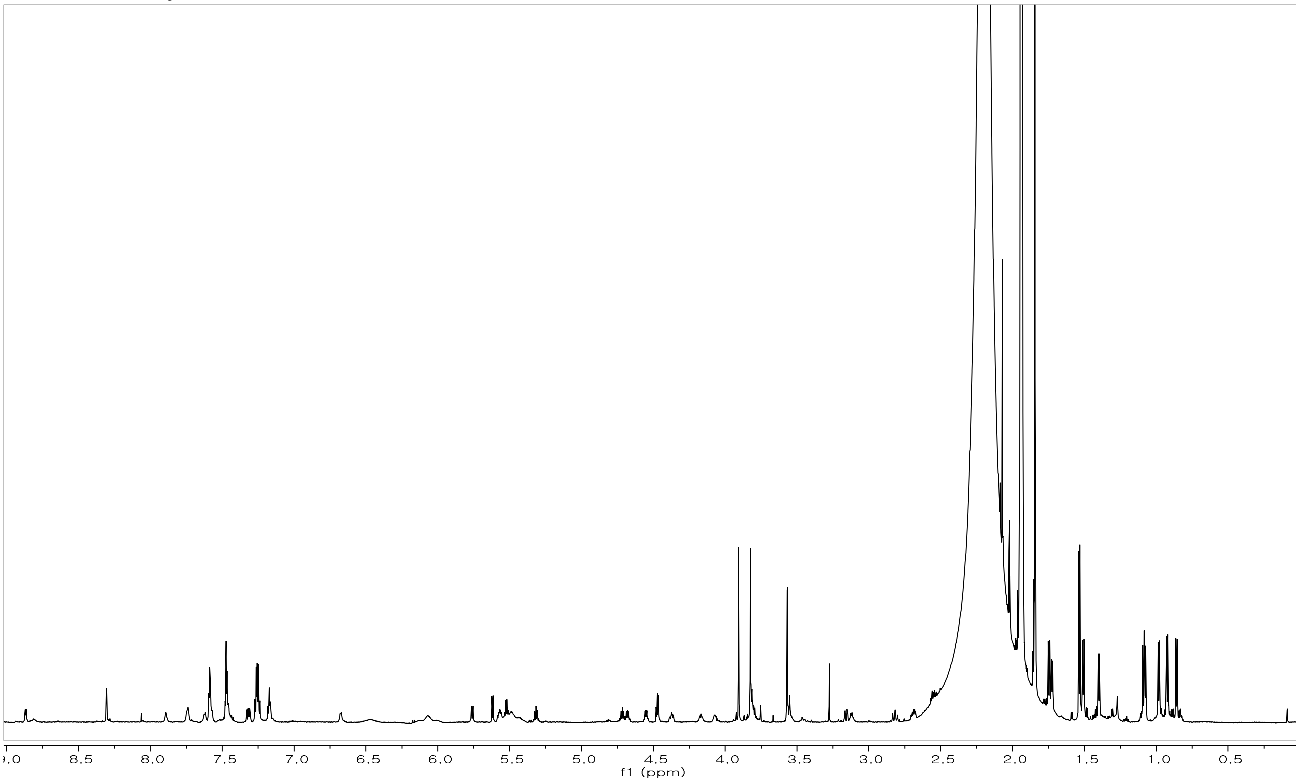

Figure S30. HSQC spectrum of *R*-MTPA ester of thiogochangamide B at 800 MHz in acetonitrile-d_3.

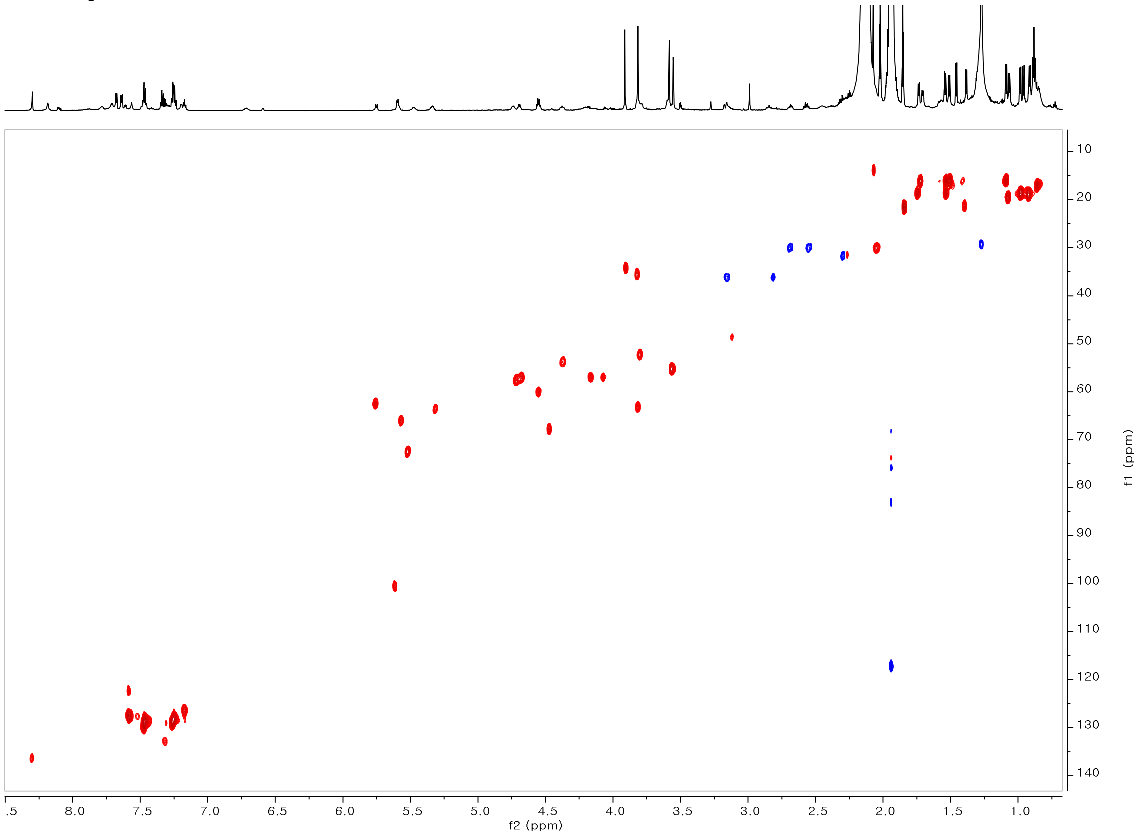

Figure S31. COSY spectrum of *R*-MTPA ester of thiogochangamide B at 800 MHz in acetonitrile-d_3.

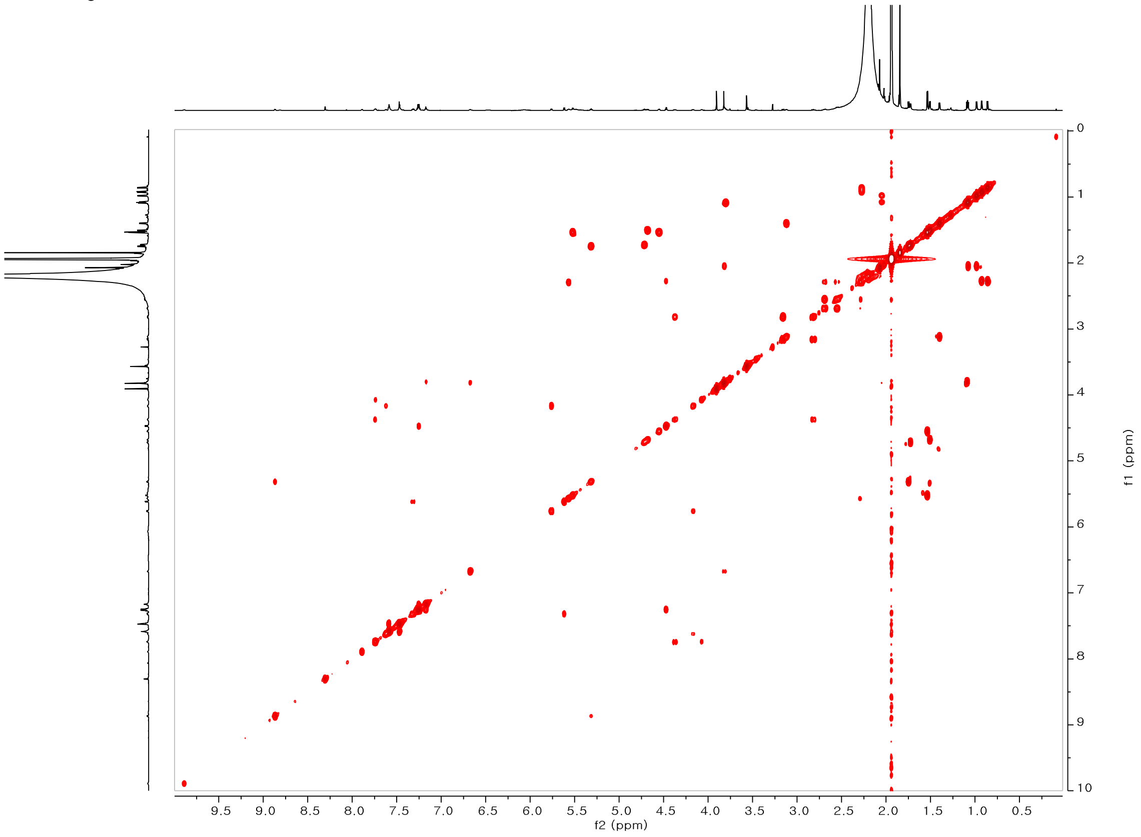

Figure S32. ^{1}H NMR spectrum of 4-(2-amino-2-carboxyethyl)-1,3-dimethyl-1H-imidazol-3-ium (**3**) at 400MHz in acetonitrile-*d*$_3$

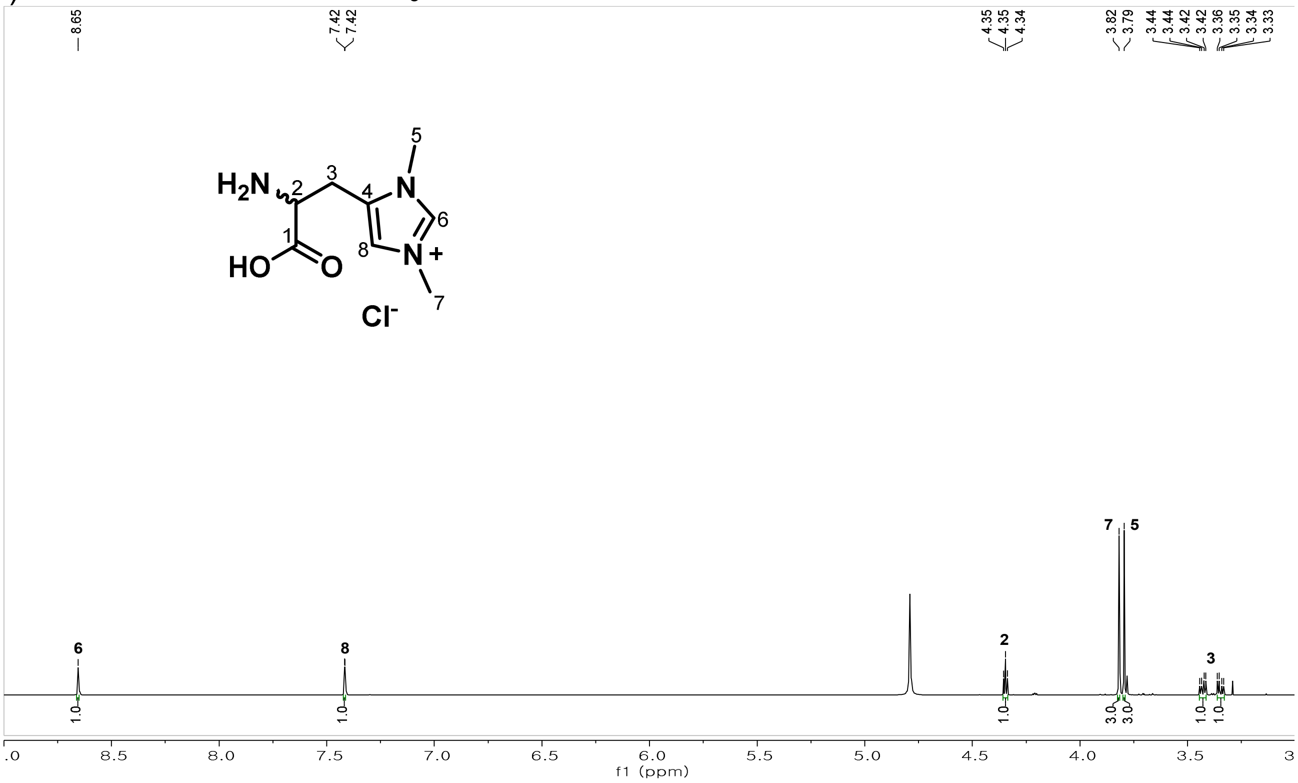

Figure S33. ^{1}H NMR spectrum of *N*,*N*-dimethylhistidinium D-FDAA-1 (**4**) at 850 MHz in DMSO-*d*$_6$

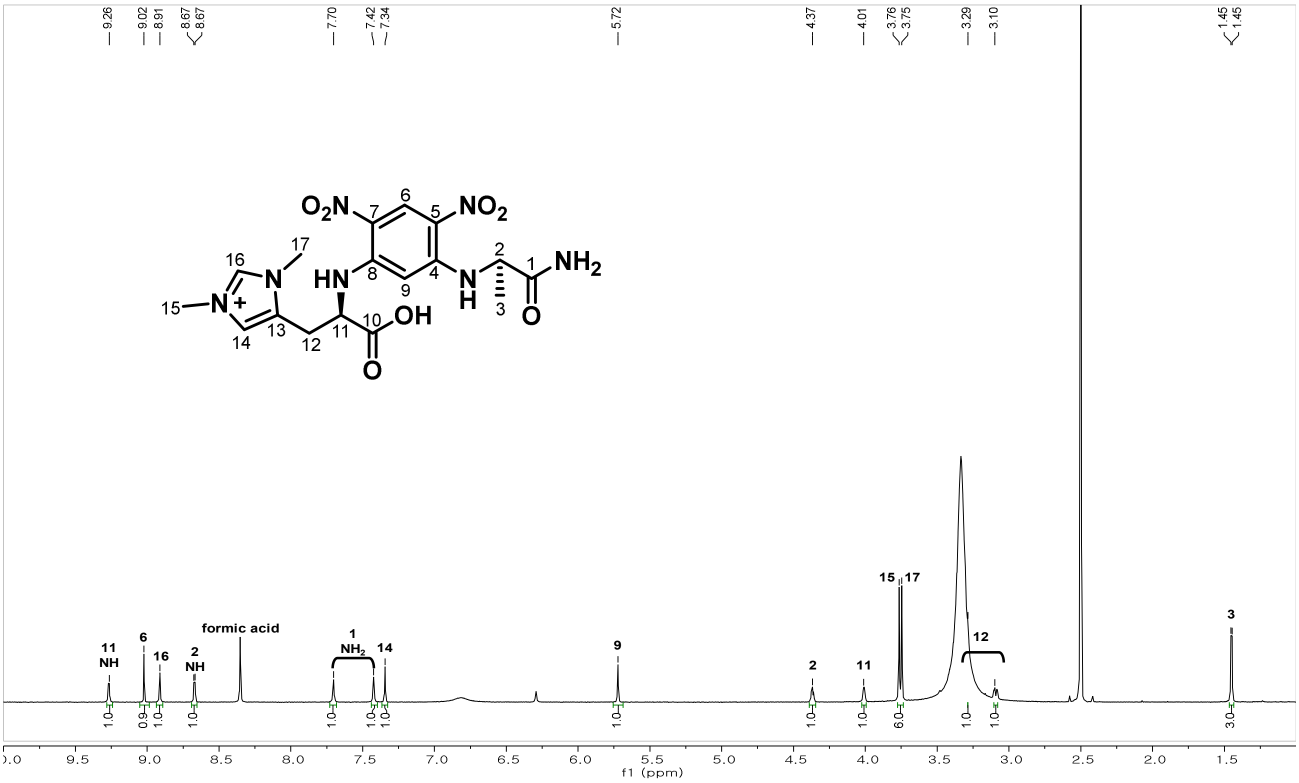

Figure S34. ^{13}C NMR spectrum of *N*,*N*-dimethylhistidinium D-FDAA-1 (**4**) at 215 MHz in DMSO-d_6.

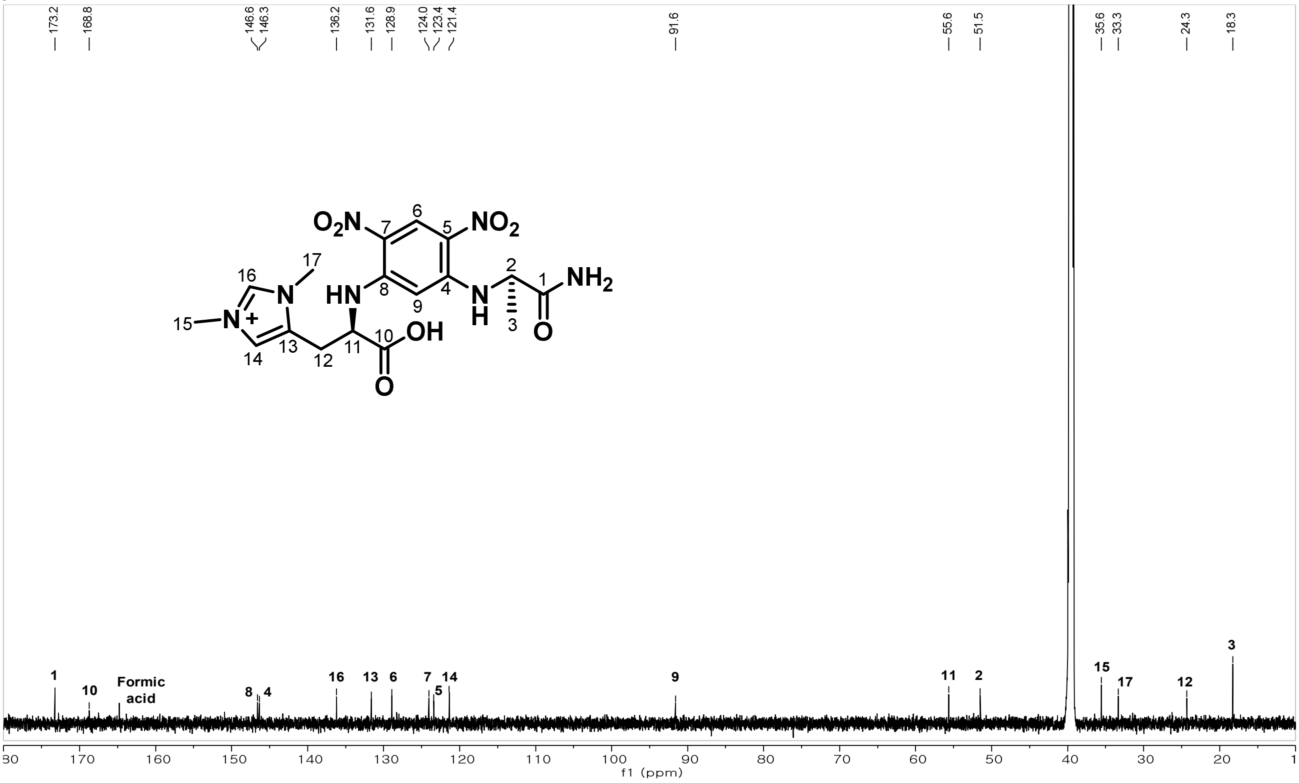

Figure S35. HSQC NMR spectrum of *N*,*N*-dimethylhistidinium D-FDAA-1 (**4**) at 850 MHz in DMSO-d_6.

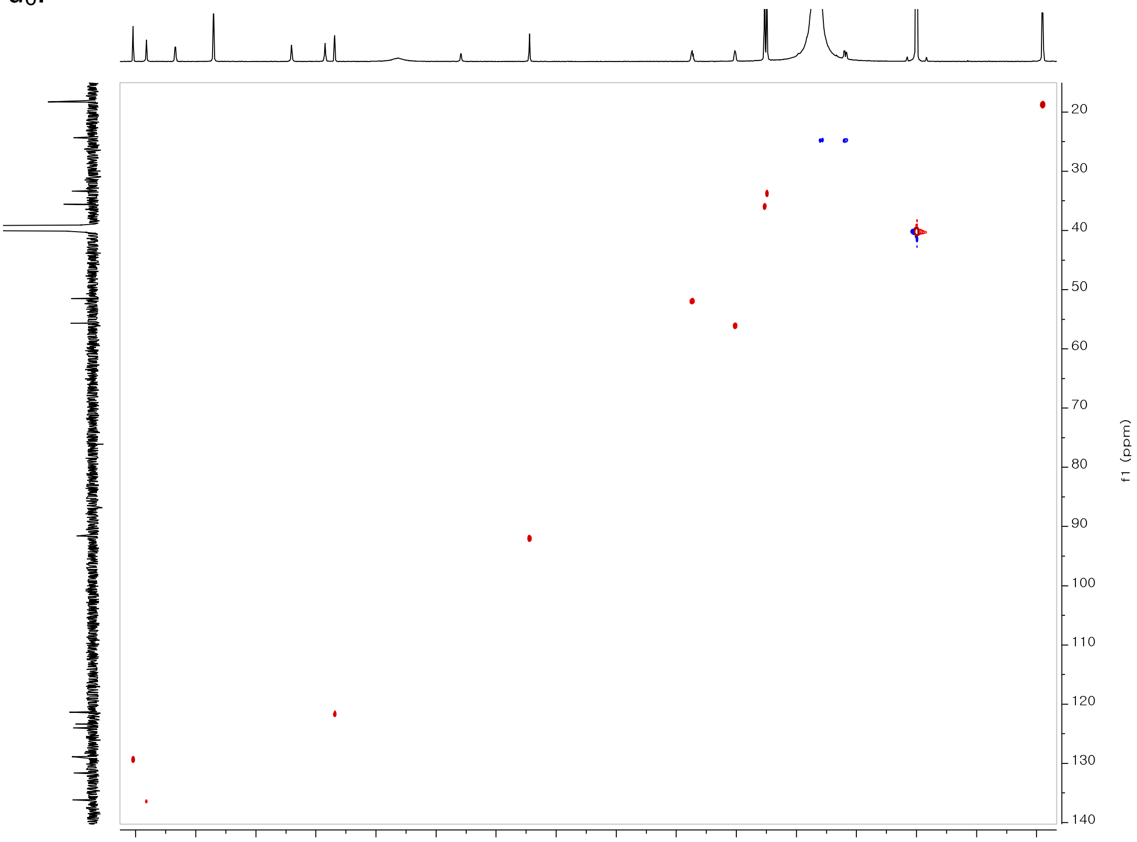

Figure S36. COSY NMR spectrum of *N*,*N*-dimethylhistidinium D-FDAA-1 (**4**) at 850 MHz in DMSO-d_6.

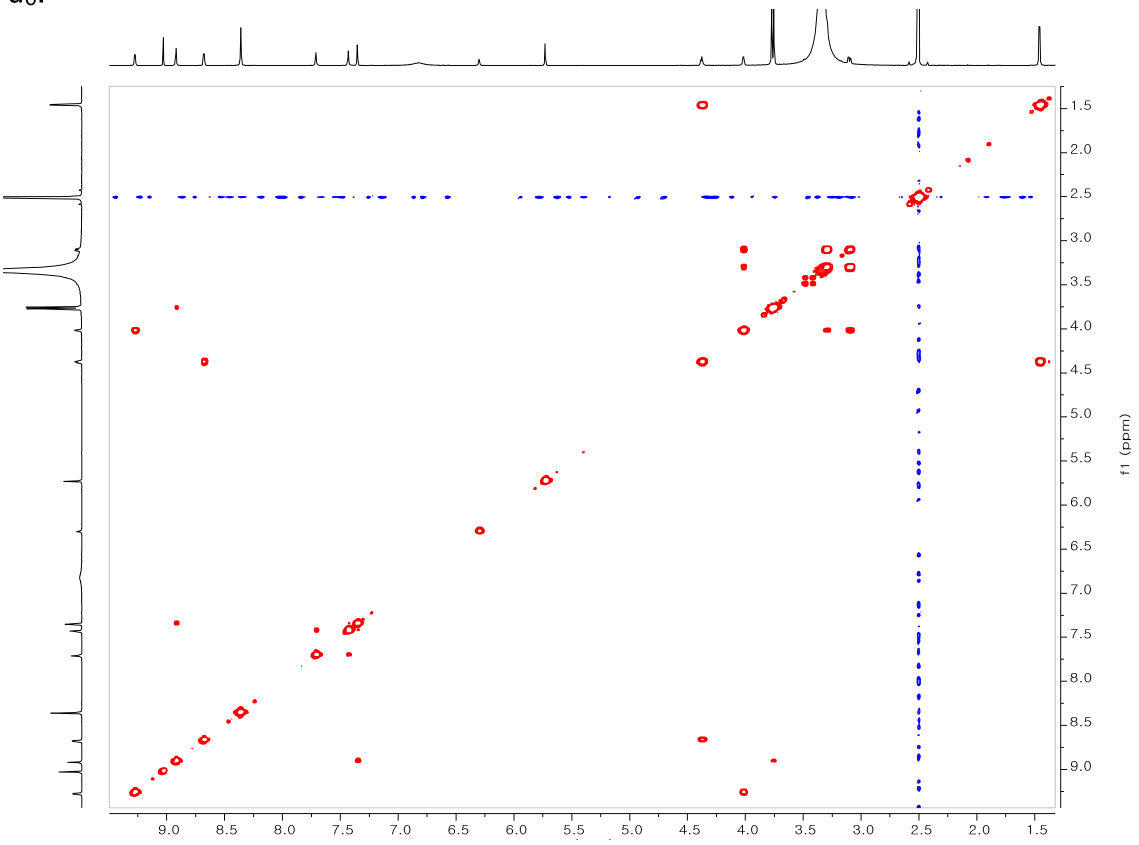

Figure S37. HMBC NMR spectrum of *N*,*N*-dimethylhistidinium D-FDAA-1 (**4**) at 850 MHz in DMSO-d_6.

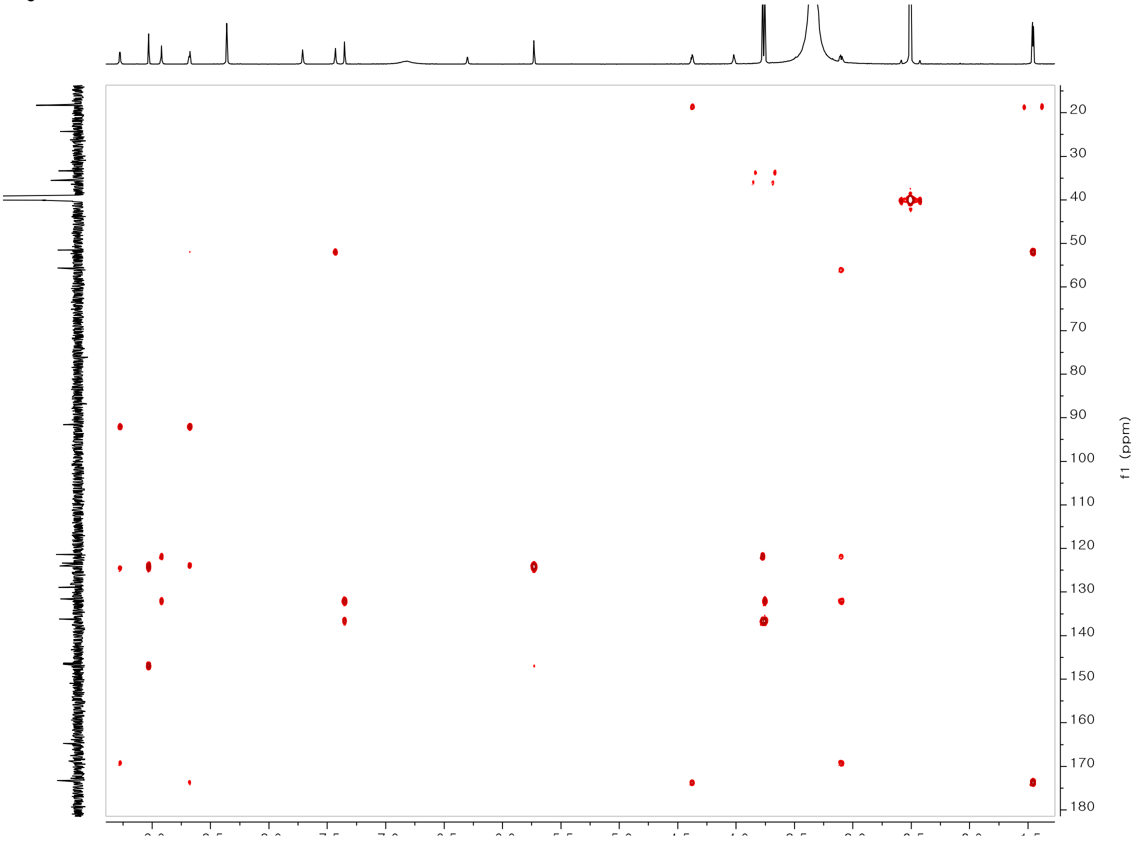

Figure S38. ROESY NMR spectrum of *N*,*N*-dimethylhistidinium D-FDAA-1 (**4**) at 850 MHz in DMSO-d_6.

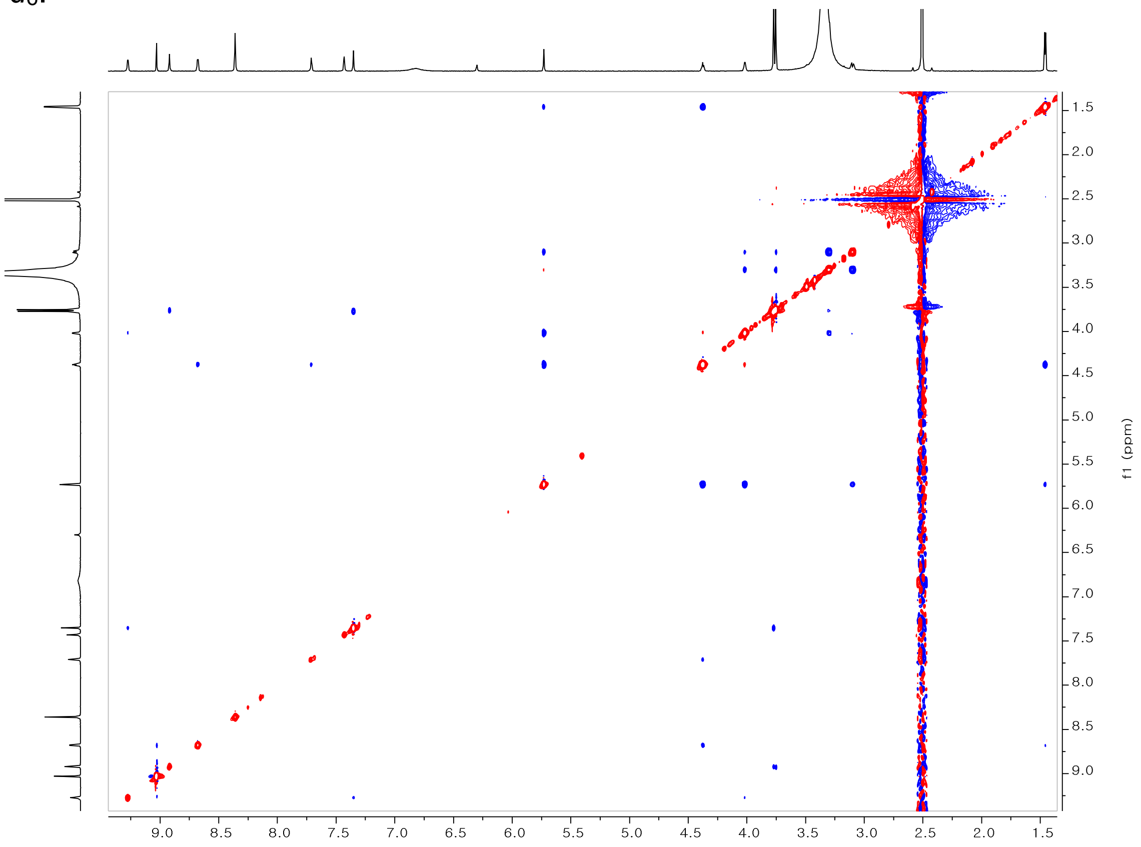

Figure S39. ^{1}H NMR spectrum of *N*,*N*-dimethylhistidinium D-FDAA-2 (**5**) at 800 MHz in DMSO-d_6.

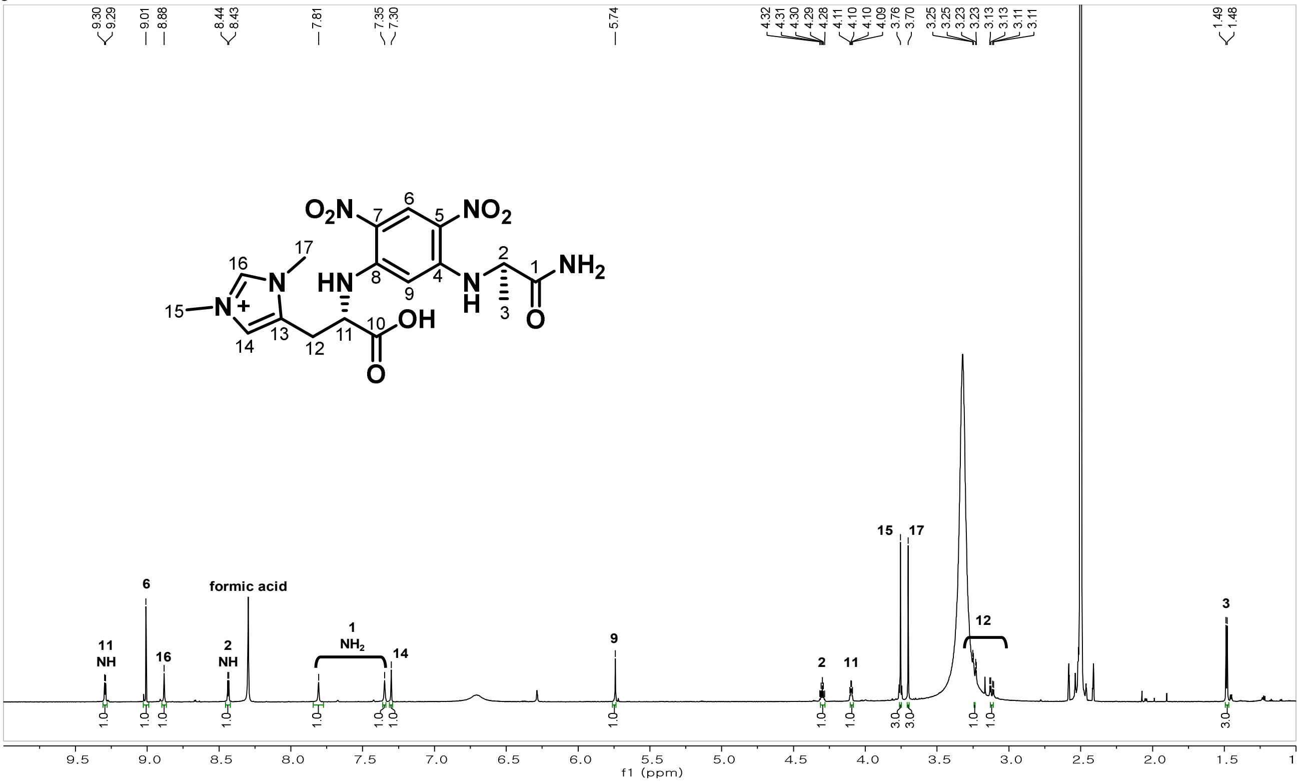

Figure S40. ^{13}C NMR spectrum of *N*,*N*-dimethylhistidinium D-FDAA-2 (**5**) at 200 MHz in DMSO-*d*$_6$.

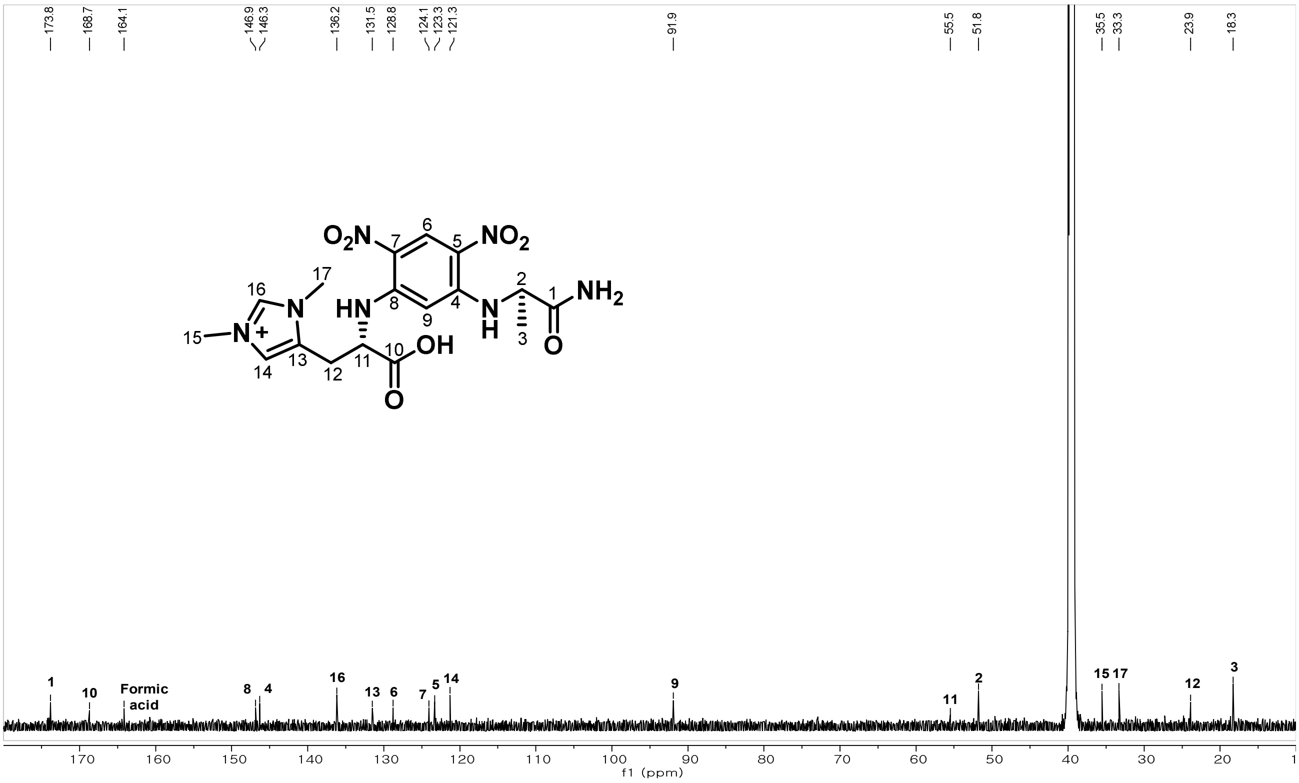

Figure S41. HSQC NMR spectrum of *N*,*N*-dimethylhistidinium D-FDAA-2 (**5**) at 800 MHz in DMSO-*d*$_6$.

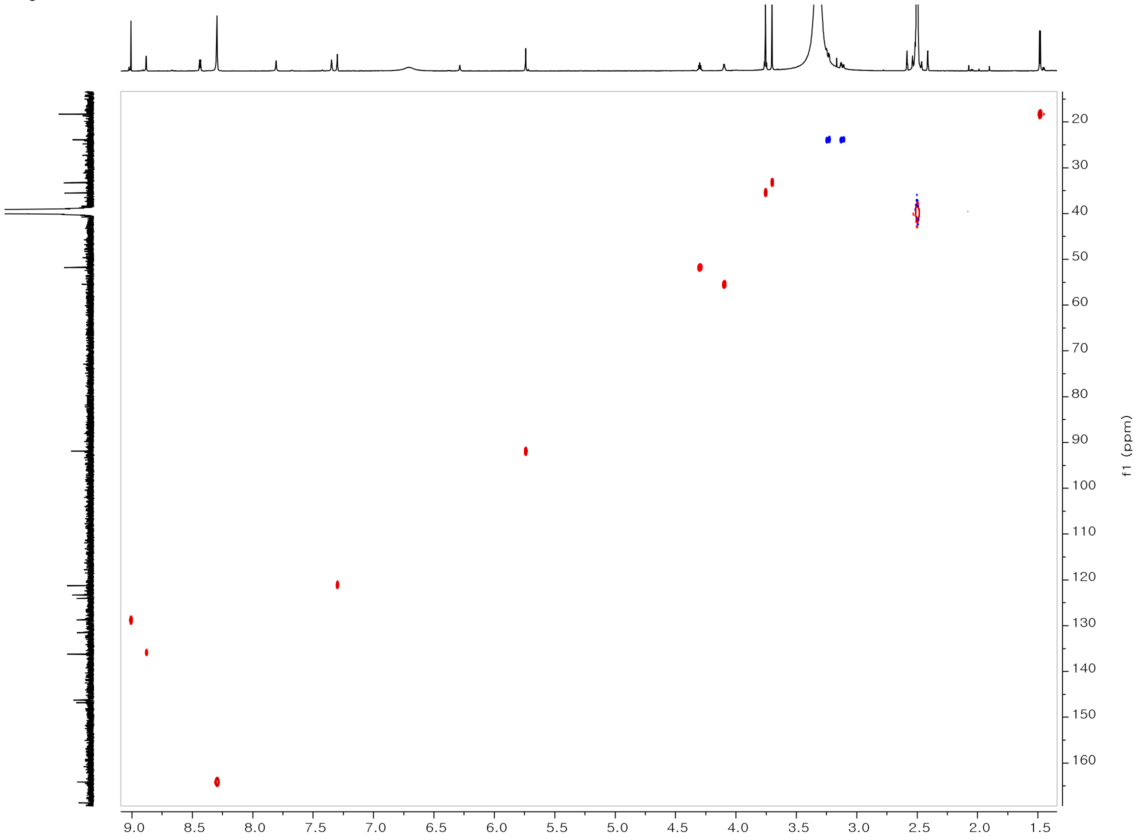

Figure S42. COSY NMR spectrum of *N*,*N*-dimethylhistidinium D-FDAA-2 (**5**) at 800 MHz in DMSO-d_6.

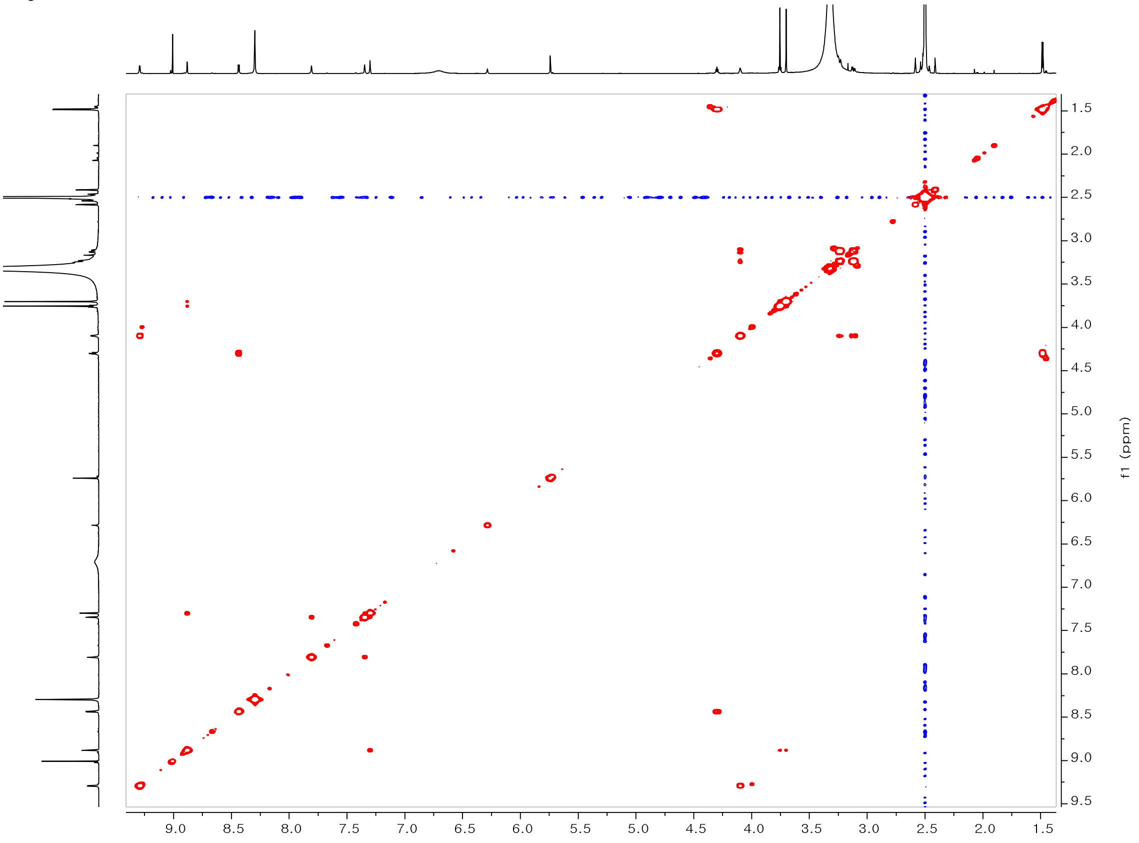

Figure S43. HMBC NMR spectrum of *N*,*N*-dimethylhistidinium D-FDAA-2 (**5**) at 800 MHz in DMSO-d_6.

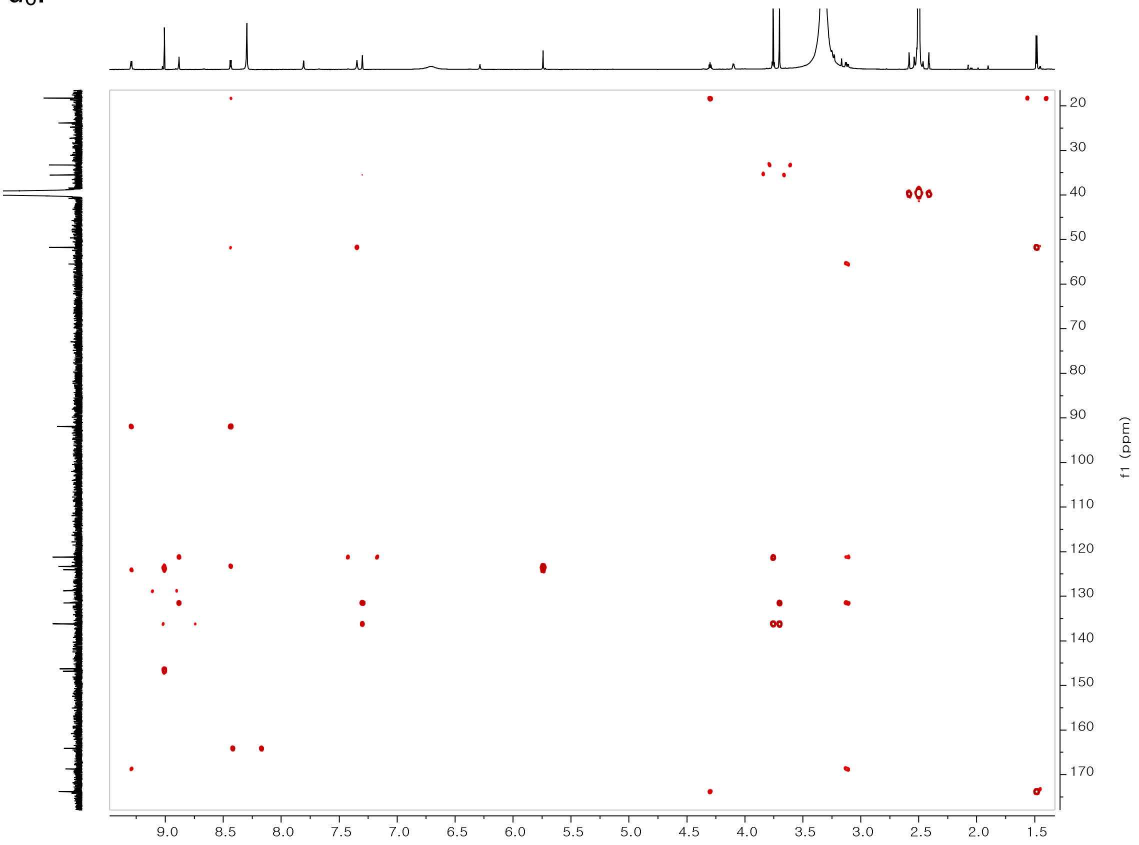

Figure S44. ROESY NMR spectrum of *N*,*N*-dimethylhistidinium D-FDAA-2 (**5**) at 800 MHz in DMSO-d_6.

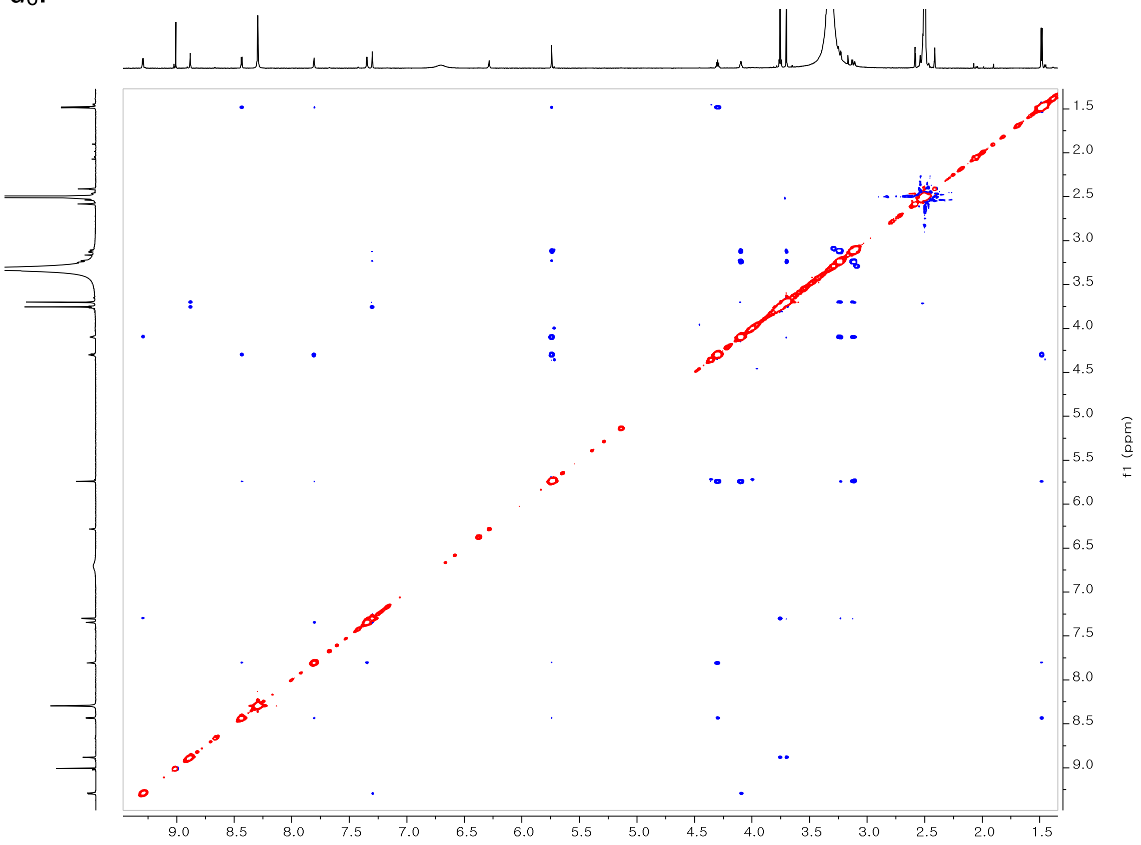

Determination of stereochemistry

Figure S45. HR-MS data of thiogochangamide B *S* MTPA ester (**2a**) and *R* MTPA ester (**2b**).

Thiogochangamide B *S* MTPA ester (2a)

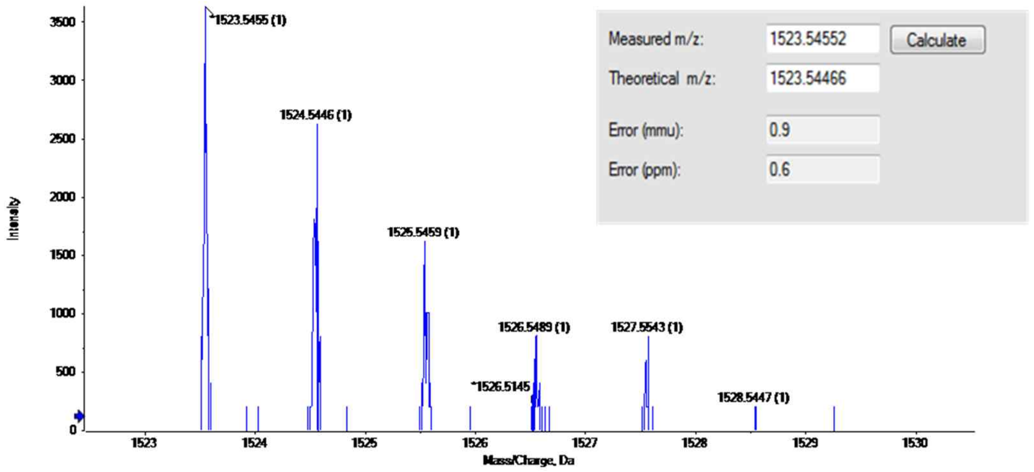

Thiogochangamide B *R* MTPA ester (2b)

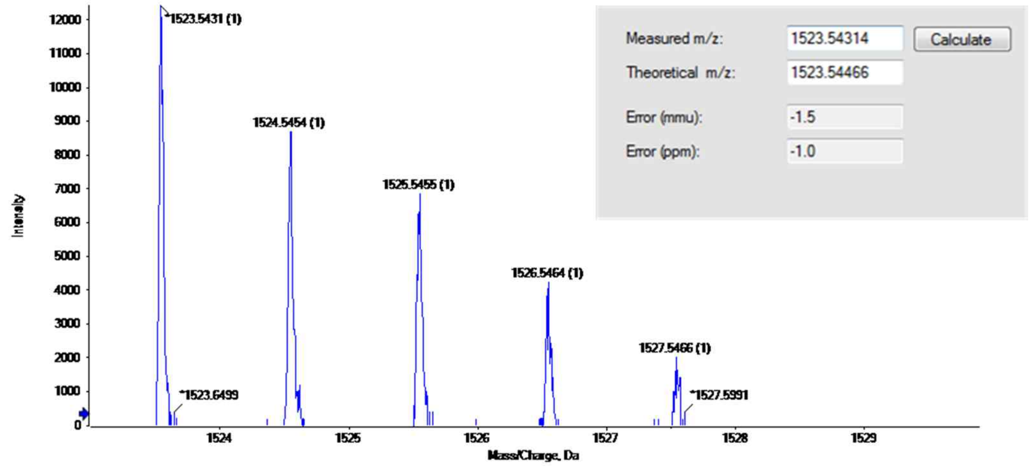

Figure S46. $\Delta\delta_{S-R}$ values of (*S*)- and (*R*)-MTPA esters of thiogochangamide B (**2**).

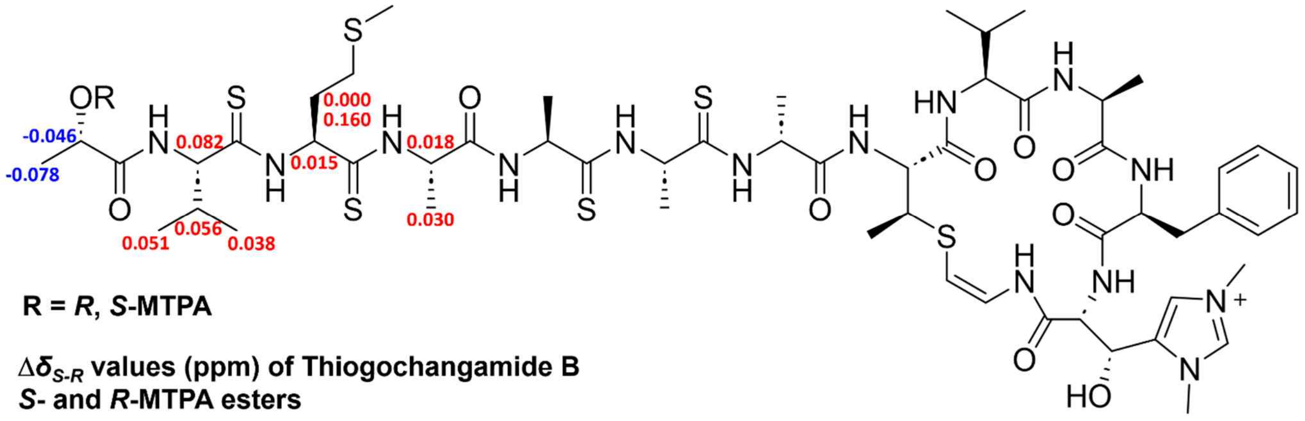

Table S1. LC/MS analysis of D- and L-FDAA derivatives of the amino acid-derived units in thiogochangamide A (**1**) and B (**2**)

thiogochangamide A (**1**)	valine	phenylalanine	*N*, *N*-dimethyl histidinium	alanine	
+ D-FDAA	32.41	35.93	**11.41**	**21.84**	24.87
+ L-FDAA	**28.15**	**32.57**	11.65	24.84	**21.85**
Elution order	**L** → D	**L** → D	D → L	**D** → L	**L** → D
Area ratio				**1**	**2**

thiogochangamide B (**2**)	valine	phenylalanine	*β*-hydroxy-*N*, *N*-dimethyl histidinium	alanine	
+ D-FDAA	32.28	35.81	10.38	**21.51**	24.65
+ L-FDAA	**27.93**	**32.36**	**9.91**	24.64	**21.52**
Elution order	**L** → D	**L** → D	L → D	**D** → L	**L** → D
Area ratio				**1**	**2**

Figure S47. Partial hydrolysis of thiogochangamide B (**2**) and HR-MS data.

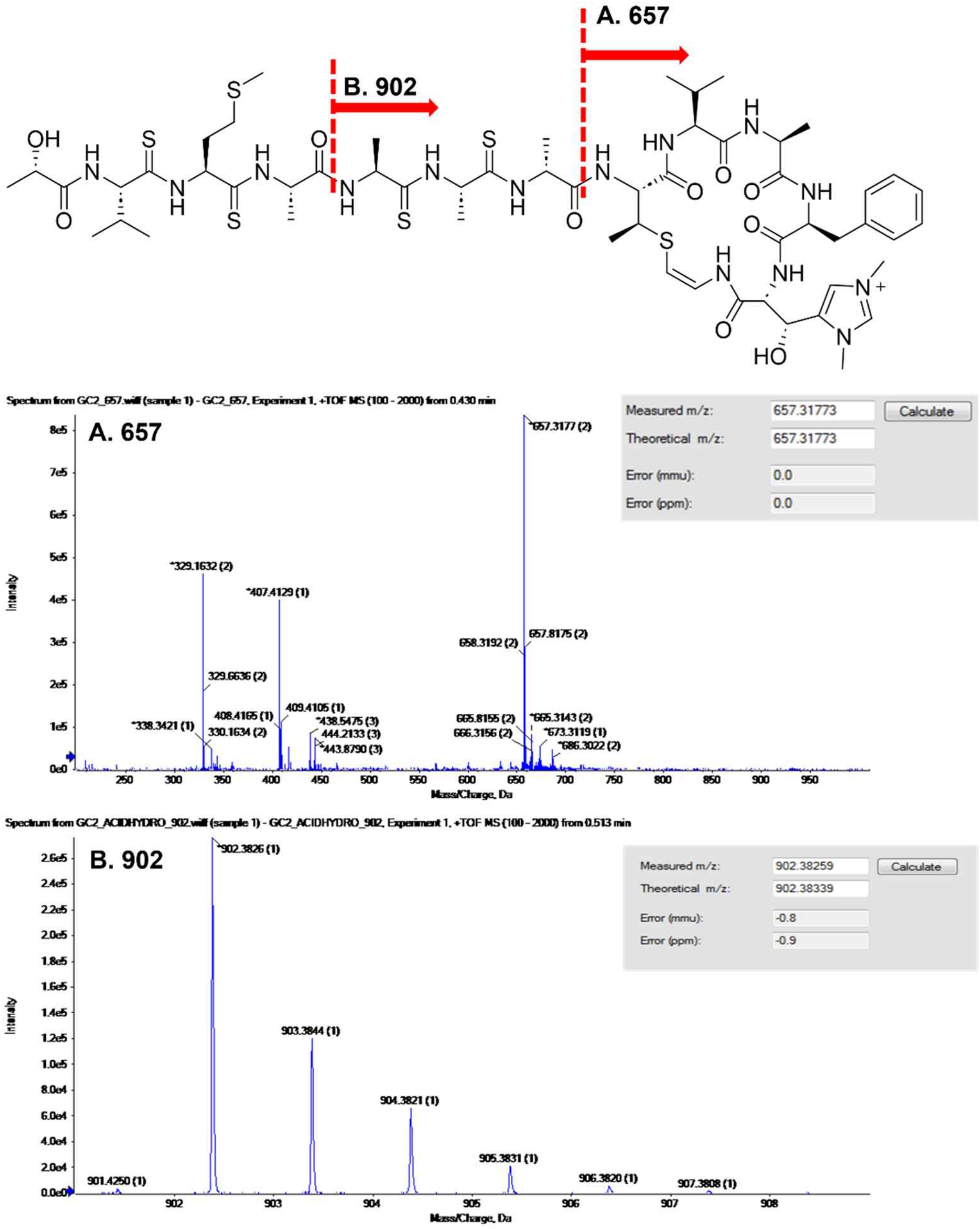

Table S2. LC/MS analysis of L-FDAA derivatives of hydrolyzed thiogochangamide B.

+ L-FDAA	alanine
thiogochangamide B (**2**)	23.6:26.4 = 2:1
657 (partial)<br>Absolute stereochemistry	23.6<br>L-Ala
902 (partial)<br>Absolute stereochemistry	23.5:26.3 = 1:1<br>L-Ala, D-Ala

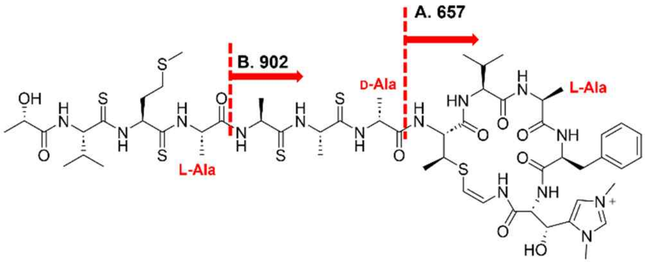

Authentic standard	+ L-FDAA
L-alanine	23.6
D-alanine	26.3

Figure S48. HR-MS data of amidized in thiogochangamide B and HR-MS/MS data with a possible structure.

(a)

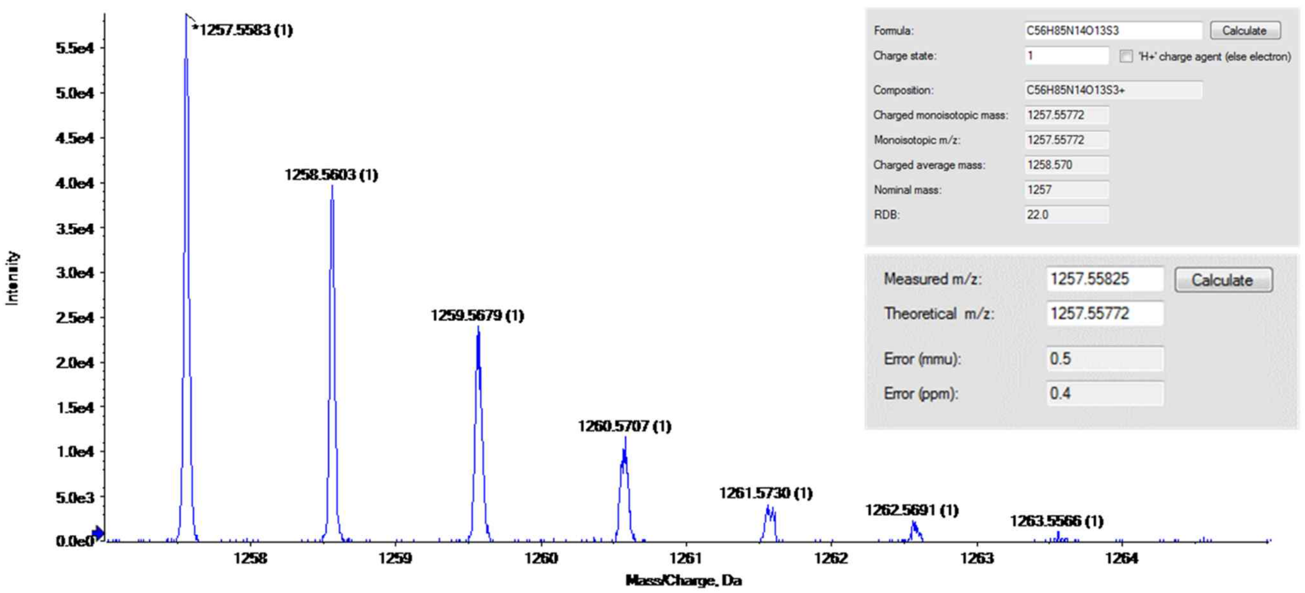

(b)

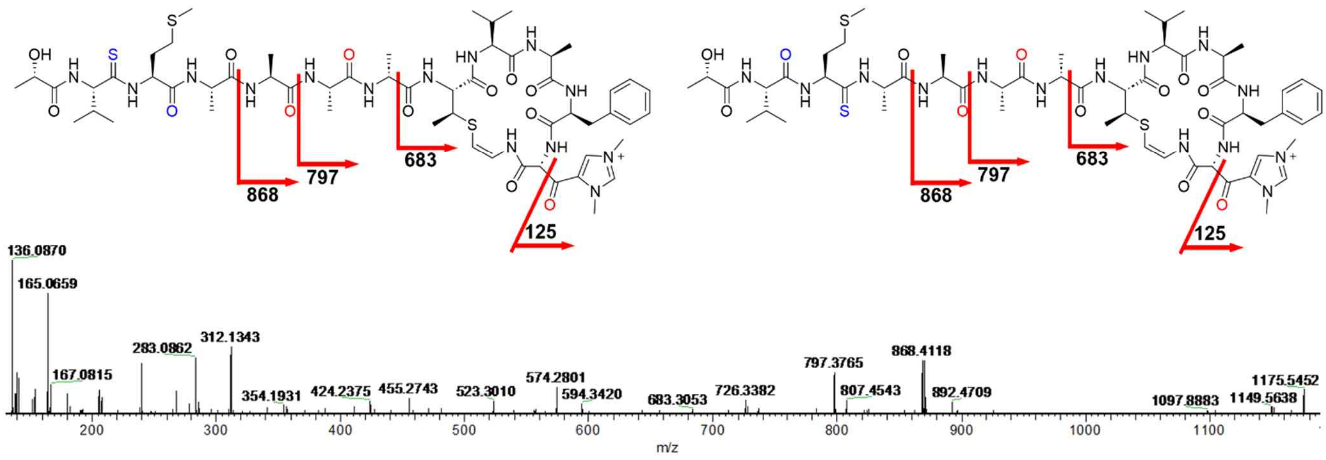

Table S3. LC/MS analysis of D-FDAA derivatives of amidized thiogochangamide B.

	Area ratio		Alanine area ratio
thiogochangamide B (**2**) + D-FDAA	valine < phenylalanine		1 : ~2
amidized thiogochangamide B + D-FDAA	valine > phenylalanine		1 : ~4
Retention time	33min	37min	21.8 min : 24.8 min
Result	The thioamide attached to one valine was changed to amide		Thioamide, which was attached to two alanine, were changed to amide and identified L-form

Figure S49. Scheme for 4-(2-amino-2-carboxyethyl)-1,3-dimethyl-1 H-imidazol-3-ium (**3**) synthesis.

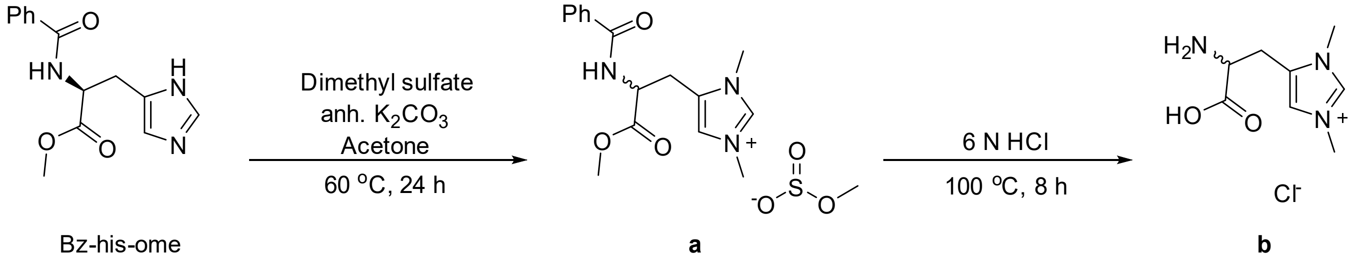

Figure S50. LC/MS chromatogram of D- and L-FDAA derivatives of the synthetic *N*, *N*-dimethylhistidinium-derived units.

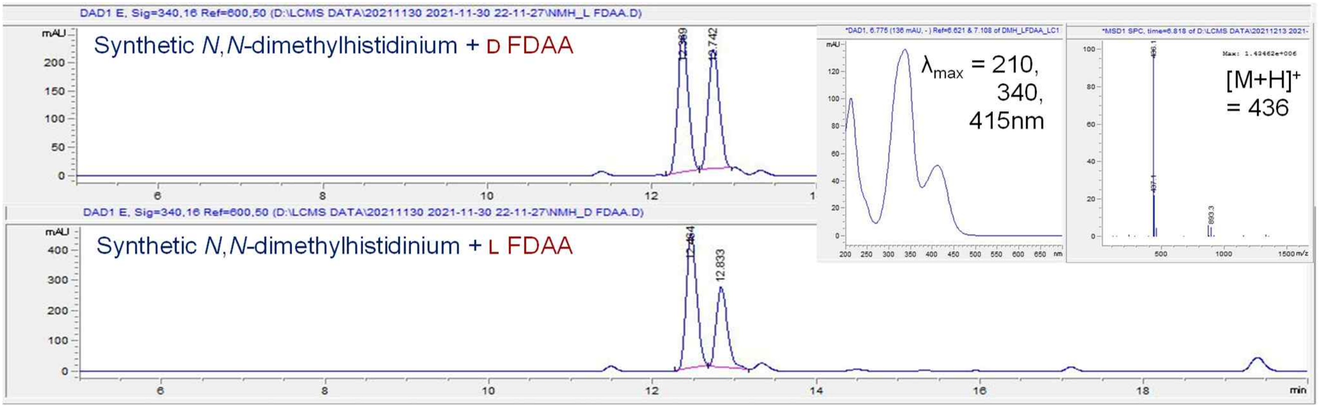

Table S4. The major conformers of (D-*N*,*N*-dimethylhistidinium + D FDAA) and (L-*N*,*N*-dimethylhistidinium + D FDAA) identified by conformational searches in MMFF94 force field using MacroModel.

**Conformers**[a]	**Boltzmann population (%)**[b]
(D-*N*,*N*-dimethylhistidinium + D FDAA)-1	28.154
(D-*N*,*N*-dimethylhistidinium + D FDAA)-2	25.459
(D-*N*,*N*-dimethylhistidinium + D FDAA)-3	23.998
(D-*N*,*N*-dimethylhistidinium + D FDAA)-4	14.371
(D-*N*,*N*-dimethylhistidinium + D FDAA)-5	3.999
(D-*N*,*N*-dimethylhistidinium + D FDAA)-6	1.845
(D-*N*,*N*-dimethylhistidinium + D FDAA)-7	0.877
(D-*N*,*N*-dimethylhistidinium + D FDAA)-8	0.796
(D-*N*,*N*-dimethylhistidinium + D FDAA)-9	0.502
(L-*N*,*N*-dimethylhistidinium + D FDAA)-1	46.837
(L-*N*,*N*-dimethylhistidinium + D FDAA)-2	9.612
(L-*N*,*N*-dimethylhistidinium + D FDAA)-3	9.562
(L-*N*,*N*-dimethylhistidinium + D FDAA)-4	9.321
(L-*N*,*N*-dimethylhistidinium + D FDAA)-5	7.985
(L-*N*,*N*-dimethylhistidinium + D FDAA)-6	7.302
(L-*N*,*N*-dimethylhistidinium + D FDAA)-7	7.200
(L-*N*,*N*-dimethylhistidinium + D FDAA)-8	2.182

Table S5. Experimental (Exp.) chemical shift values of **4** and **5** with calculated (Cal.) chemical shift values (CS, *δ*) of (D-*N*,*N*-dimethylhistidinium + D FDAA) and (L-*N*,*N*-dimethylhistidinium + D FDAA).

No.	**Exp. CS of** *N*,*N*-dimethylhistidinium D-FDAA-1 (**4**)	**Exp. CS of** *N*,*N*-dimethylhistidinium D-FDAA-2 (**5**)	**Cal. CS of** (D-*N*,*N*-dimethylhistidinium + D FDAA)	**Cal. CS of** (L-*N*,*N*-dimethylhistidinium + D FDAA)
C-1	136.2	136.2	132	133.93
C-4	131.6	131.5	137.63	136.86
C-5	121.4	121.3	126.3	128.69
C-6	33.3	33.3	39.04	38.22
C-10	35.6	35.5	39.42	39.28
C-14	24.3	23.9	30.37	30.05
C-15	55.6	55.5	68.38	62.99
C-19	168.8	168.7	170.48	172.63
C-24	146.6	146.9	145.32	148.05
C-26	124	124	134.53	135.90
C-27	91.6	91.9	114.05	115.48
C-28	146.3	146.3	149.39	149.15
C-29	128.9	128.8	130.79	132.02
C-30	123.4	123.3	136.71	137.78
C-40	51.5	51.8	63.2	61.35
C-42	173.2	173.8	171.58	174.55
C-43	18.3	18.3	23.89	23.48
H-7	3.75	3.7	3.29	3.21
H-8	3.75	3.7	3.41	3.45
H-9	3.75	3.7	3.37	3.39
H-11	3.77	3.75	3.23	3.52
H-12	3.77	3.75	3.5	3.45
H-13	3.77	3.75	3.49	3.43
H-16	3.09	3.12	2.63	2.61
H-17	3.29	3.24	2.58	2.66
H-22	4.01	4.1	3.54	3.50
H-25	9.27	9.3	6.65	6.71
H-31	5.72	5.74	6.1	6.15
H-32	9.02	9.01	8.27	8.34
H-41	8.67	8.43	5.28	5.34
H-44	1.45	1.49	1.3	1.28
H-45	1.45	1.49	1.51	1.42
H-46	1.45	1.49	0.99	0.91
H-47	4.37	4.3	3.35	3.24
H-52	7.34	7.3	6.97	6.74
H-53	8.91	8.88	6.74	6.9

Figure S51. CP3 calculation result of *N*, *N*-dimethylhistidinium D-FDAA derivatives

Calc A – (D-*N*,*N*-dimethylhistidinium + D FDAA)
Calc B – (L-*N*,*N*-dimethylhistidinium + D FDAA)
Expt A – *N*,*N*-dimethylhistidinium D-FDAA-1
Expt B – *N*,*N*-dimethylhistidinium D-FDAA-2

TOTAL

CP3 thinks ExpA goes with CalcA and ExpB with CalcB
ExpA goes with CalcA and ExpB with CalcB : 100.0%
ExpA goes with CalcB and ExpB with CalcA : 0.0%

CP3 values

	C data	H data	All data
ExpA - CalcA and ExpB - CalcB :	-1.31	-0.20	-0.75
ExpA - CalcB and ExpB - CalcA :	-3.25	-0.01	-1.63

Probabilities

	C data	H data	All data
ExpA - CalcA and ExpB - CalcB :	100%	25.1%	100.0%
ExpA - CalcB and ExpB - CalcA :	0.0%	74.9%	0.0%

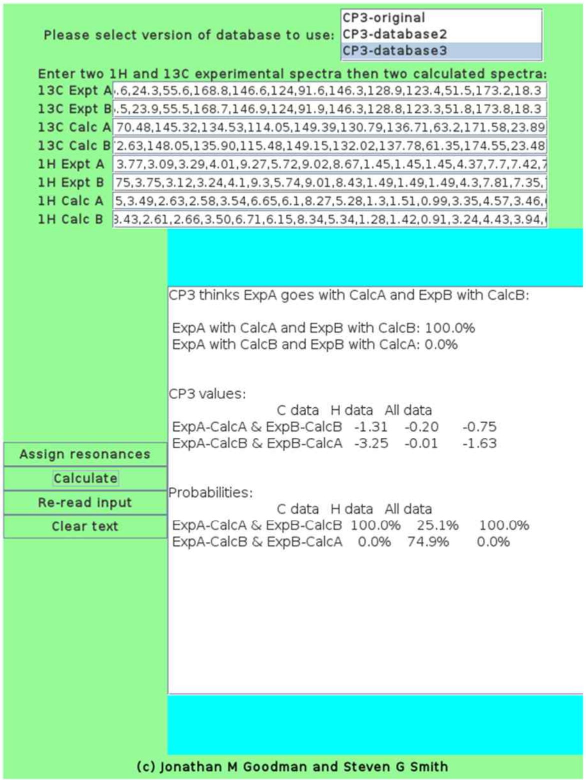

Figure S.52 *J*-resolved HMBC spectrum of thiogochangamide B (**1**) at 800 MHz in acetonitrile-*d*3 and determination absolute stereochemistry of *β*-hydroxyl-*N*, *N*-dimethylhistidinium.

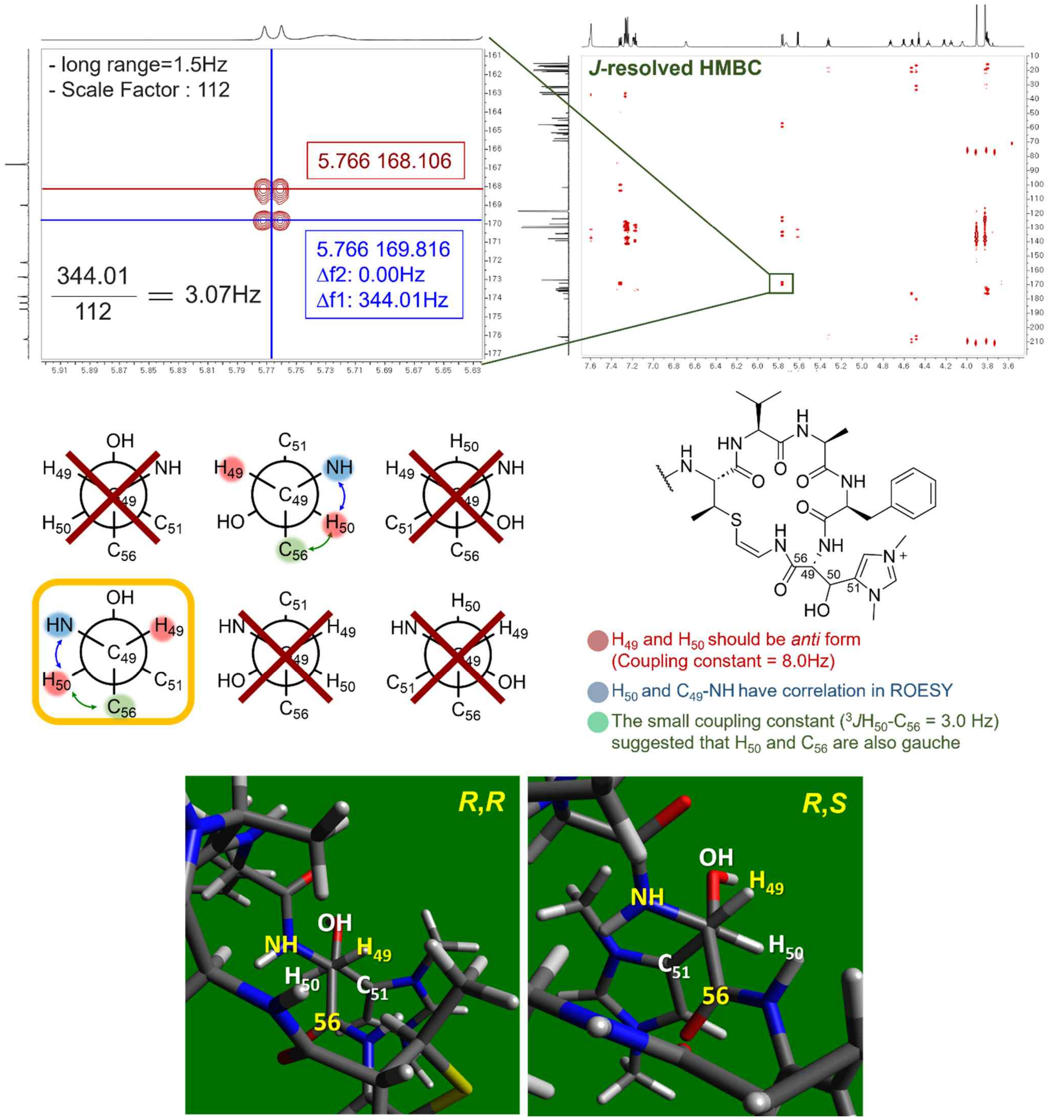

Figure S53. ROESY data analysis of thiogochangamide B (**2**) and determination absolute stereochemistry of *β*-hydroxyl-*N*, *N*-dimethylhistidinium.

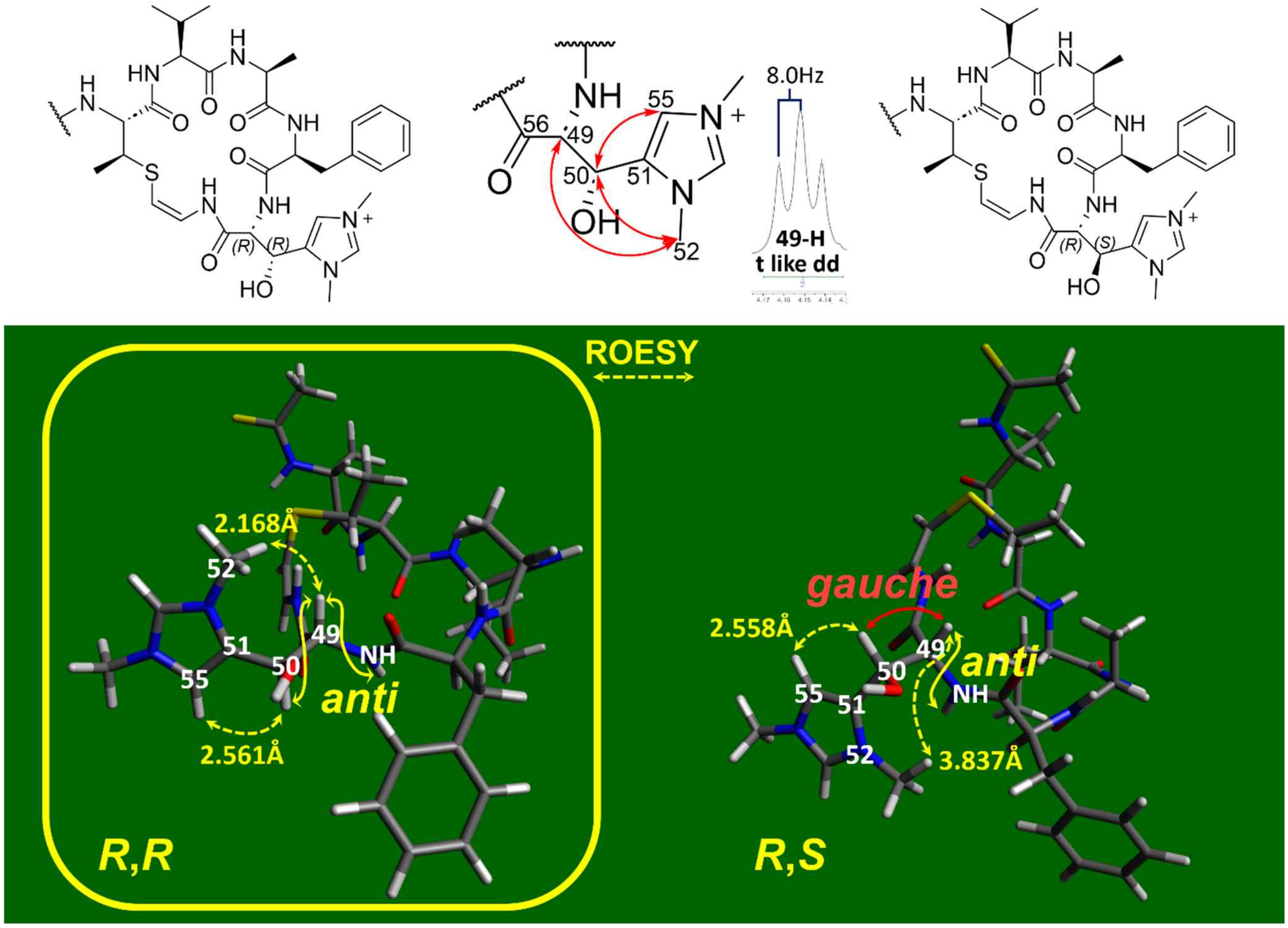

Figure S54. DFT modeling results of D-*N*, *N*-dimethylhistidinium and *β*-(*R*)-hydroxyl-D-*N*, *N*-dimethylhistidinium.

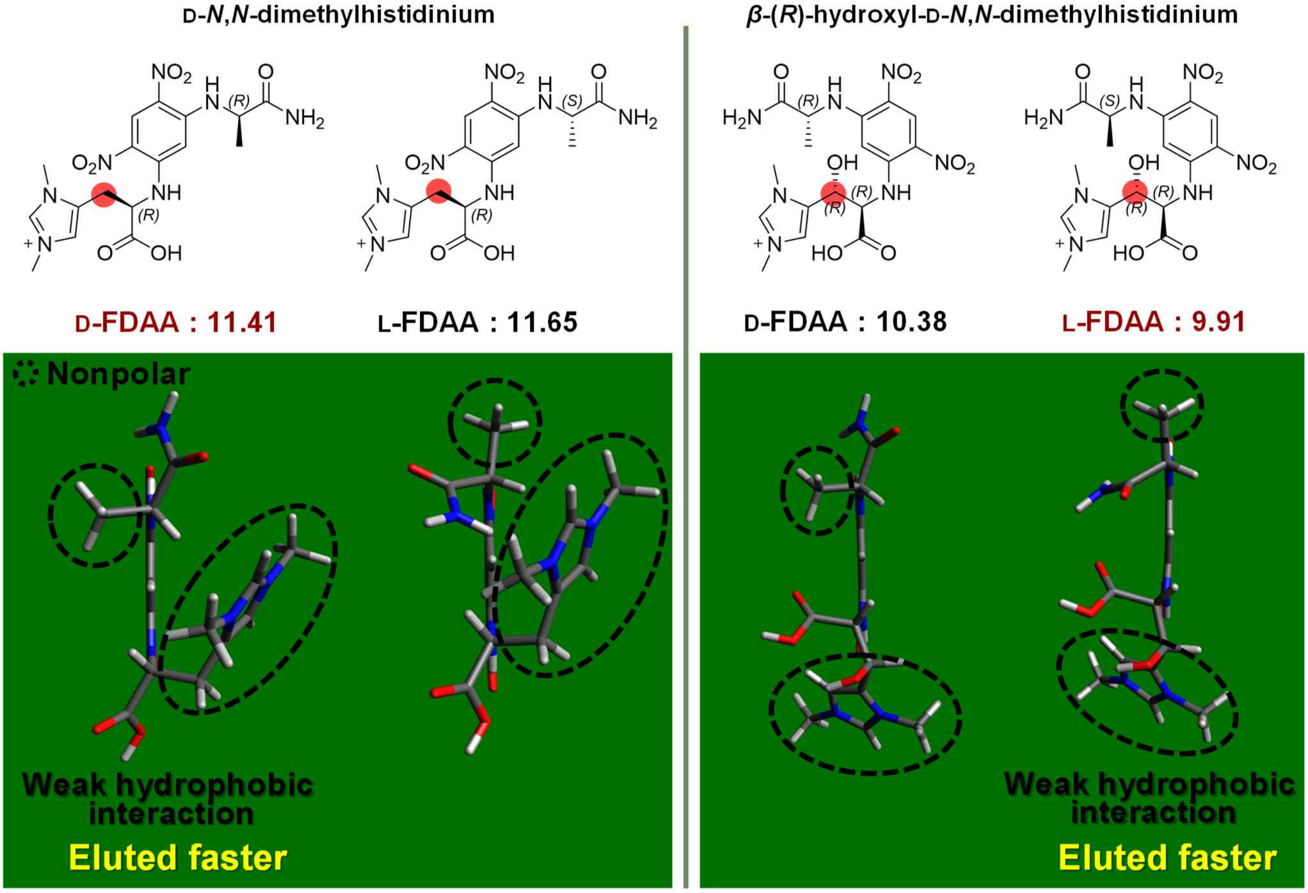

Figure S55. The simulated models of four possible diastereomers (**a**–**d**) of thiogochangamide A (**1**) and the result of DP4 calculation.

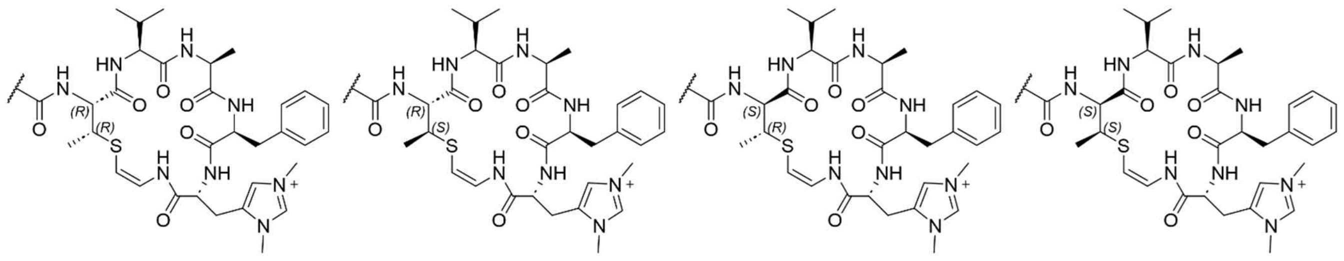

Diastereomer 1a (26*R* and 27*R*) **Diastereomer 1b (26*R* and 27*S*)** **Diastereomer 1c (26*S* and 27*R*)** **Diastereomer 1d (26*S* and 27*S*)**

Results of DP4 using the carbon and proton data

26*R* and 27*R* : 0.0% **26*R* and 27*S* : 100.0%** **26*S* and 27*R* : 0.0%** **26*S* and 27*S* : 0.0%**

Results of DP4 using the carbon data only

26*R* and 27*R* : 0.0% 26*R* and 27*S* : 100.0% 26*S* and 27*R* : 0.0% 26*S* and 27*S* : 0.0%

Results of DP4 using the proton data only

26*R* and 27*R* : 0.0% 26*R* and 27*S* : 100.0% 26*S* and 27*R* : 0.0% 26*S* and 27*S* : 0.0%

Table S6. The major conformers of cyclic partial structure identified by conformational searches in MMFF94 force field using MacroModel.

Conformers[a]	Boltzmann population (%)[b]
Diastereomer 1a_1	31.771
Diastereomer 1a_2	15.184
Diastereomer 1a_3	11.744
Diastereomer 1a_4	11.202
Diastereomer 1a_5	8.174
Diastereomer 1a_6	3.096
Diastereomer 1a_7	2.949
Diastereomer 1a_8	2.586
Diastereomer 1a_9	2.481
Diastereomer 1a_10	2.096
Diastereomer 1a_11	1.889
Diastereomer 1a_12	1.406
Diastereomer 1a_13	1.303
Diastereomer 1a_14	1.147
Diastereomer 1a_15	0.891
Diastereomer 1a_16	0.791
Diastereomer 1a_17	0.661
Diastereomer 1a_18	0.627
Diastereomer 1b_1	45.426
Diastereomer 1b_2	17.728
Diastereomer 1b_3	10.448
Diastereomer 1b_4	4.212
Diastereomer 1b_5	3.373
Diastereomer 1b_6	2.387
Diastereomer 1b_7	2.032
Diastereomer 1b_8	1.925
Diastereomer 1b_9	1.72
Diastereomer 1b_10	1.69
Diastereomer 1b_11	1.687
Diastereomer 1b_12	1.634
Diastereomer 1b_13	1.563
Diastereomer 1b_14	1.227
Diastereomer 1b_15	1.067

Diastereomer 1b_16	0.945
Diastereomer 1b_17	0.937
Diastereomer 1c_1	31.777
Diastereomer 1c_2	14.141
Diastereomer 1c_3	8.783
Diastereomer 1c_4	6.801
Diastereomer 1c_5	6.118
Diastereomer 1c_6	4.972
Diastereomer 1c_7	4.928
Diastereomer 1c_8	4.346
Diastereomer 1c_9	3.67
Diastereomer 1c_10	3.003
Diastereomer 1c_11	2.71
Diastereomer 1c_12	2.631
Diastereomer 1c_13	1.398
Diastereomer 1c_14	1.215
Diastereomer 1c_15	0.987
Diastereomer 1c_16	0.761
Diastereomer 1c_17	0.701
Diastereomer 1c_18	0.658
Diastereomer 1c_19	0.632
Diastereomer 1d_1	27.711
Diastereomer 1d_2	26.829
Diastereomer 1d_3	16.52
Diastereomer 1d_4	13.304
Diastereomer 1d_5	3.196
Diastereomer 1d_6	2.328
Diastereomer 1d_7	1.647
Diastereomer 1d_8	1.343
Diastereomer 1d_9	1.17
Diastereomer 1d_10	0.931
Diastereomer 1d_11	0.852
Diastereomer 1d_12	0.787
Diastereomer 1d_13	0.76
Diastereomer 1d_14	0.75
Diastereomer 1d_15	0.7

Diastereomer 1d_16	0.656
Diastereomer 1d_17	0.516

Table S7. Experimental (Exp.) and calculated (Cal.) chemical shift values (CS, *δ*) of cyclic diastereomers.

No.	Exp. CS of Thiogochangamide A (1)	Cal CS of diastereomer 1a	Cal CS of diastereomer 1b	Cal CS of diastereomer 1c	Cal CS of diastereomer 1d
2C	58.1	64.7	64.53	61.72	62.24
3C	172	178.51	173.78	177.89	177.73
6C	64.4	60.19	60.81	60.84	59.19
7C	31.1	34.29	34.93	34.03	34.79
8C	20.7	27.14	26.37	27.61	27.41
9C	19.6	21.26	23.1	21.02	21.27
10C	174.1	172.98	176.48	173.16	174.31
13C	53.4	56.63	56.72	56.46	56.89
14C	174.8	175.65	172.19	175.04	177.12
17C	54.9	64.75	61.25	64.62	65.31
18C	172.9	174.5	174.21	175.61	175
21C	37.3	41.91	41.95	40.97	42.13
22C	139.4	134.42	134.2	135.18	135
23C	130.1	129.6	128.11	128.84	128.96
24C	129	130.6	129.43	129.68	130.52
25C	127.4	128.47	128.3	128.35	128.5
26C	129	130.55	129.72	130.64	130.42
27C	130.1	127.79	130.74	128.41	128.48
28C	54.2	57.11	60.11	57.23	56.26
29C	168	167.78	166.34	166.36	168.04
30C	22.9	29.18	29.81	28.98	30.73
31C	134	140.43	139.65	141.1	141.56
33C	136.7	133.98	133.06	132.71	132.45
35C	122.7	127.23	126.48	127.56	127.55
36C	34.4	38.95	39.29	39.48	39.05
37C	36.6	38.9	39.2	38.91	38.88
40C	134.7	134.11	131.96	132.99	133.71
41C	100.3	99.13	104	99.43	103.51
43C	49.5	47.87	53.85	48.25	60.27
44C	22.4	25.81	26.63	25.35	28.05
45C	17.1	21.79	22.66	21.84	21.66
46C	174	169.6	170.75	170.36	169.27
50H	4	3.93	2.75	3.87	4.01
52H	3.75	3.42	3.46	3.35	3.56
53H	2.04	1.35	1.85	1.34	1.38
54H	1.08	0.81	0.69	0.82	0.83
55H	1.08	0.33	0.47	0.32	0.37
56H	1.08	0.79	0.6	0.94	0.87

57H	0.96	0.69	0.62	0.69	0.72
58H	0.96	0.76	0.89	0.6	0.73
59H	0.96	0.47	0.08	0.52	0.62
61H	3.84	3.02	3.17	3.01	3.18
63H	4.47	3.3	4.08	3.36	3.28
65H	2.85	2.38	2.91	2.77	2.39
66H	3.19	2.96	2.65	2.97	2.87
67H	7.31	6.53	6.74	6.58	6.66
68H	7.29	7.05	6.92	6.88	7
69H	7.18	6.89	6.81	6.87	6.87
70H	7.29	6.98	6.84	6.99	6.99
71H	7.31	6.79	6.7	6.79	6.66
72H	4.22	4.12	4.01	4.03	4.09
73H	3.46	2.85	2.78	2.82	2.9
74H	3.29	2.56	2.63	2.67	2.39
75H	8.28	6.79	6.77	6.69	6.72
76H	7.48	6.82	6.44	6.8	6.86
77H	3.75	3.29	3.71	3.26	3.28
78H	3.75	3.6	3.78	3.69	3.61
79H	3.75	3.38	3.28	3.74	3.66
80H	3.81	3.34	3.29	3.29	3.18
81H	3.81	3.29	3.53	3.28	3.42
82H	3.81	3.32	3.28	3.36	3.42
84H	7.3	6.56	6.4	6.68	6.6
85H	5.55	4.74	4.75	4.72	4.89
86H	3.08	3.09	2.76	2.37	2.22
87H	1.38	1.17	1.24	1.26	1.39
88H	1.38	0.88	0.71	1.07	0.79
89H	1.38	1.24	0.73	1.04	1.4
90H	1.1	1.18	0.86	0.71	0.8
91H	1.1	0.75	0.93	0.23	0.28
92H	1.1	0.26	0.68	1.29	1.33

Figure S56. The simulated models of two possible diastereomers (**e** and **f**) of thiogochangamide B (**2**) and the result of DP4 calculation.

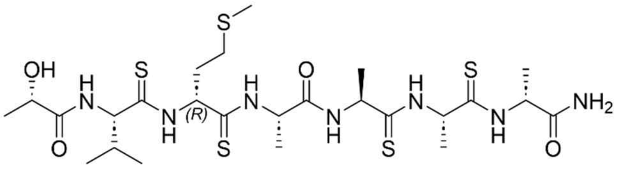

Diastereomer 2e (9*R*)

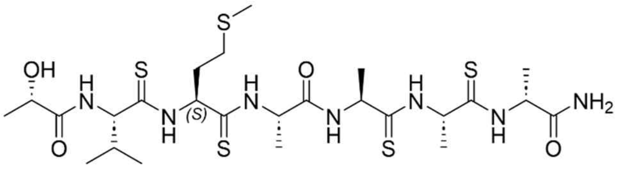

Diastereomer 2f (9*S*)

Results of DP4 using the carbon and proton data
9*R* : 0.0%   9*S* : 100.0%

Results of DP4 using the carbon data only
9*R* : 2.3%   9*S* : 97.7%
Results of DP4 using the proton data only
9*R* : 0.0%   9*S* : 100.0%

Table S8. The major conformers of linear partial structure identified by conformational searches in MMFF94 force field using MacroModel.

Conformers[a]	Boltzmann population (%)[b]
Diastereomer 2e_1	39.553
Diastereomer 2e_2	9.024
Diastereomer 2e_3	7.4
Diastereomer 2e_4	7.169
Diastereomer 2e_5	6.08
Diastereomer 2e_6	5.508
Diastereomer 2e_7	5.502
Diastereomer 2e_8	5.498
Diastereomer 2e_9	2.636
Diastereomer 2e_10	2.292
Diastereomer 2e_11	1.293
Diastereomer 2e_12	1.269
Diastereomer 2e_13	1.259
Diastereomer 2e_14	1.056
Diastereomer 2e_15	1.016
Diastereomer 2e_16	0.961
Diastereomer 2e_17	0.857
Diastereomer 2e_18	0.853
Diastereomer 2e_19	0.775
Diastereomer 2f_1	39.553
Diastereomer 2f_2	9.024
Diastereomer 2f_3	7.4
Diastereomer 2f_4	7.169
Diastereomer 2f_5	6.08
Diastereomer 2f_6	5.508
Diastereomer 2f_7	5.502
Diastereomer 2f_8	5.498
Diastereomer 2f_9	2.636
Diastereomer 2f_10	2.292
Diastereomer 2f_11	1.293

Table S9. Experimental (Exp.) and calculated (Cal.) chemical shift values (CS, *δ*) of linear diastereomers.

No.	Exp. CS of Thiogochangamide B (2)	Cal CS of diastereomer 2e	Cal CS of diastereomer 2f
C1	67.7	73.28	70.89
C2	206.4	213.91	214.26
C5	58.4	57.12	56.31
C6	176.3	173.78	176.80
C7	16.6	20.4	21.84
C10	61.4	65.84	68.32
C11	209.1	215.74	217.75
C12	19.4	20.76	20.91
C15	64.9	60.01	64.31
C16	206.4	217.29	216.42
C17	19.4	23.5	23.74
C19	58.8	56.61	58.25
C20	173.9	177.15	178.84
C24	31.9	35.94	37.49
C25	31.3	41.15	39.11
C27	15.1	24.59	23.13
C28	206.7	213.82	220.16
C29	69.2	68.15	66
C32	32.2	33.9	34.61
C33	20.2	26.17	27.44
C34	17.7	25.85	22.39
C35	179.9	177.84	178.61
C36	69.3	72.86	73.13
C38	20.8	24.99	25.13
C41	17.1	22.02	24.01
H42	5.73	4.72	4.43
H44	4.6	4.01	4.26
H45	1.5	0.99	1.01
H46	1.5	0.83	0.93
H47	1.5	0.72	0.58
H49	4.52	3.68	3.41
H50	1.57	0.78	0.77
H51	1.57	0.59	0.59
H52	1.57	2.13	1.95
H54	5.33	4.62	4.52
H55	1.79	1.02	1.16

H56	1.79	0.81	1.04
H57	1.79	1.32	1.17
H59	4.73	4.02	3.95
H63	2.24	1.73	1.57
H64	2.52	1.64	1.61
H65	2.53	1.82	1.89
H66	2.67	2.09	1.85
H67	2.07	1.53	1.25
H68	2.07	1.5	1.43
H69	2.07	1.25	1.4
H70	4.46	3.39	3.95
H72	2.47	1.9	1.91
H73	1.06	0.59	0.68
H74	1.06	0.88	0.87
H75	1.06	0.13	0.69
H76	0.96	0.59	0.56
H77	0.96	0.94	0.95
H78	0.96	0.24	0.33
H79	4.22	3.44	3.51
H80	1.27	1.07	1.06
H81	1.27	0.78	0.85
H82	1.27	0.75	0.87
H84	1.75	0.74	1.19
H85	1.75	1.09	0.94
H86	1.75	1.1	0.59

Table S10. ^{1}H NMR and ^{13}C NMR spectral data NMR of oxidized-thiostreptamide S4 in DMSO-d_6.

Position	oxidized-thiostreptamide S4				
	^{1}H NMR δ_H, mult (*J* in Hz)	^{13}C NMR δ_C	Position	^{1}H NMR δ_H, mult (*J* in Hz)	^{13}C NMR δ_C
**Residue 1**	**pyruvic acid**		**Residue 8**	***β*-methyl-*S*-aminovinyl cysteine**	
**1**	2.34, s	24.7	**29-NH**	Not detected	
**2**		196	**29**	4.75, broad s	53.9
**3**		160	**30**	3.39, overlap	76.7
**Residue 2**	**valine**		**31**	1.26, d (7.0)	20.8
**4-NH**	8.25, d (9.0)		**32**	5.32, broad s	Not detected
**4**	4.56, overlap	62.9	**33**	6.92, broad s	121.6
**5**	2.14, m	33.2	**33-NH**	10.45, broad s	
**6**	0.89, overlap	19.8	**34**		169.8
**7**	0.81, overlap	17.8	**Residue 9**	**valine**	
**8**		200.4	**35-NH**	Not detected	
**Residue 3**	**methionine sulfoxide**		**35**	3.78, overlap	70.8
**9-NH**	10.29, broad s		**36**	2.18, m	33.2
**9**	5.38, overlap	62.4	**37**	0.90, overlap	20.7
**10**	2.17, overlap	27.6	**38**	0.95, overlap	19.6
**11**	2.83, overlap	48.8	**39**		170.3
**12**	2.53, overlap	38.0	**Residue 10**	**alanine**	
**13**		200.5	**40-NH**	7.65, m	
**Residue 4**	**alanine**		**40**	3.98, overlap	49.7
**14-NH**	Not detected		**41**	1.14, broad s	17.9
**14**	4.83, q (7.0)	53.8	**42**		171.0
**15**	1.36, d (7.0)	28.6	**Residue 11**	**O-methyl-tyrosine**	
**16**		169.9	**43-NH**	Not detected	
**Residue 5**	**alanine**		**43**	3.97, overlap	59.4
**17-NH**	8.38, broad s		**44**	3.27, m<br>2.54, m	35.2
**17**	4.68, overlap	54.3	**45**		129.8
**18**	1.26, overlap	20.7	**46, 50**	7.02, d (7.5)	130.1
**19**		204.5	**47, 49**	6.75, d (7.5)	113.4
**Residue 6**	**isoleucine**		**48**		157.7
**20-NH**	9.74, s		**51**	3.70, s	55.0
**20**	5.13, broad s	67.1	**52**		172.0
**21**	1.97, m	38.9	**Residue 12**	***β*-hydroxy-*N*,*N*-dimethylhistidinium**	
**22**	1.52, m<br>1.13, m	24.5	**53-NH**	Not detected	
**23**	0.88, overlap	14.8	**53**	4.65, broad s	57.4
**24**	0.82, overlap	10.9	**54**	5.43, overlap	62.3
**25**		200.9	**55**		133.6
**Residue 7**	**alanine**		**56**	3.94, s	34.3
**26-NH**	Not detected		**57**	9.00, s	137.4
**26**	5.00, broad s	54.5	**58**	3.77, s	35.2
**27**	1.42, d (7.0)	17.8	**59**	7.25, broad s	121.5
**28**		171	**60**		166.2

Table S11. ^{1}H NMR and ^{13}C NMR spectral data NMR of thiogochangamides A (**1**) and B (**2**) in Acetonitrile-*d*$_3$.

Position	Thiogochangamide A (1)			Thiogochangamide B (2)	
	**^{1}H NMR δ_H, mult (*J* in Hz)**	**^{13}C NMR δ_C**	**^{15}N NMR δ_N**	**^{1}H NMR δ_H, mult (*J* in Hz)**	**^{13}C NMR δ_C**
**Residue 1**	**Lactic acid**			**Lactic acid**	
**1**	1.26, d (7.0)	20.8		1.27 d (7.0)	20.9
**2**	4.21, m	69.3		4.22 q (7.0)	69.3
**3**		180.2			180.0
**Residue 2**	**L -valine**			**L-valine**	
**4-NH**	7.60, d (5.0)		110.8	7.60, overlap	
**4**	4.46, m	69.3		4.46, dd (5.0)	69.3
**5**	2.48, m	32.2		2.47, m	32.2
**6**	0.97, d (7.0)	17.6		0.97, d(7.0)	17.7
**7**	1.06, d (7.0)	20.2		1.06, d(7.0)	20.2
**8**		206.8			206.7
**Residue 3**	**L-methionine**			**L-methionine**	
**9-NH**	10.60, br s		164.5	10.52, br s	
**9**	5.75, m	67.8		5.73, m	67.7
**10**	2.24, m<br>2.57, m	31.9		2.24, m<br>2.52, m	31.9
**11**	2.53, m<br>2.68, m	31.4		2.53, m<br>2.67, m	31.3
**12**	2.07, s	15.2		2.07, s	15.2
**13**		206.6			206.5
**Residue 4**	**L-alanine**			**L-alanine**	
**14-NH**	Not detected		170.2	10.87, br s	
**14**	4.60, q (7.0)	58.5		4.60, q (7.0)	58.4
**15**	1.51, d (7.0)	16.6		1.50, d (7.0)	16.6
**16**		176.4			176.3
**Residue 5**	**L-alanine**			**L-alanine**	
**17-NH**	8.25 br s		120.7	8.22 br s	
**17**	4.52 qd (7.0, 3.0)	61.6		4.52 qd (7.0, 3.0)	61.4
**18**	1.58, d (7.0)	19.4		1.57, d (7.0)	19.5
**19**		209.3			209.1
**Residue 6**	**L-alanine**			**L-alanine**	
**20-NH**	8.87 d (6.0)		158.9	8.87, d (6.0)	
**20**	5.32 m	65.1		5.33, m	65.0
**21**	1.81, d (7.0)	19.4		1.79, d (7.0)	19.5
**22**		206.5			206.5
**Residue 7**	**D-alanine**			**D-alanine**	
**23-NH**	8.80 d (6.0)		162.4	8.87, d (6.0)	
**23**	4.74, m	58.9		4.73, m	58.8
**24**	1.78, d (7.0)	17.1		1.75, d (7.0)	17.1
**25**		174.0			174.0
**Residue 8**	**27-(*S*)-methyl-*S*-30-aminovinyl D-cysteine**			**27-(*S*)-methyl-*S*-30-aminovinyl D-cysteine**	
**26-NH**	7.86, d (7.0)		108.3	7.84, d (7.0)	
**26**	4.00, m	58.1		4.04, m	58.0
**27**	3.08, q (7.0)	49.5		3.10, q (7.0)	49.8
**28**	1.38, d (7.0)	22.4		1.38, d (7.0)	22.4
**29**	5.55, d (7.0)	100.3		5.62, d (7.0)	101.8
**30**	7.29, dd (11.0, 7.0)	134.7		7.32, dd (11.0, 7.0)	134.0
**30-NH**	10.47, d (11.0)			10.60, d (11.0)	
**31**		172.0			172.1
**Residue 9**	**L-valine**			**L-valine**	
**32-NH**	6.64, d (6.0)		118.6	6.69, d (6.0)	

**32**	3.75, m	64.4		3.81, m	64.3
**33**	2.04, m	31.1		2.05, m	31.1
**34**	0.96, d (7.0)	19.6		0.98, d (7.0)	19.6
**35**	1.08, d (7.0)	20.7		1.09, d (7.0)	20.6
**36**		174.1			174.3
**Residue 10**	**L-alanine**			**L-alanine**	
**37-NH**	7.19, overlap		121.3	7.19, overlap	
**37**	3.84, m	53.4		3.80, m	53.4
**38**	1.10, d (7.0)	17.1		1.09, d (7.0)	17.0
**39**		174.8			174.6
**Residue 11**	**L-phenylalanine**			**L-phenylalanine**	
**40-NH**	7.84, d (8.5)		107.0	7.80, d (8.5)	
**40**	4.48, m	54.9		4.37, m	54.8
**41**	2.85, dd (13.0, 13.0)<br>3.19, dd (13.0, 3.0)	37.3		2.82, dd (13.0, 13.0)<br>3.15, dd (13.0, 3.0)	37.1
**42**		139.4			139.2
**43, 47**	7.31, d (7.0)	130.2		7.27, overlap	130.2
**44, 46**	7.29, dd (7.0, 7.0)	129.0		7.25, overlap	129.1
**45**	7.18, overlap	127.4		7.17, overlap	127.4
**48**		172.9			172.9
**Residue 12**	**D-*N*,*N*-dimethylhistidinium**			***β*-(*R*)-hydroxy-D-*N*,*N*-dimethylhistidinium**	
**49-NH**	7.63, d (7.0)		112.9	7.61 overlap	
**49**	4.22, m	54.2		4.15, dd (8.0, 8.0)	58.0
**50**	3.29, ddd (16.0, 4.0, 0.5)<br>3.46, ddd (16.0, 11.0, 0.5)	22.9		5.77, d (8.0)	63.6
**51**		134.0			134.3
**52**	3.75, s	34.4		3.91, s	35.4
**53**	8.28, s	136.7		8.31, s	137.9
**53-N**			171.9		
**54**	3.81, s	36.6		3.83, s	36.7
**55**	7.48, br s	122.7		7.60, br s	123.7
**55-N**			169.6		
**56**		168.0			169.0

Biosynthetic gene cluster (BCG) analysis

Table S12. Primers used in this study.

Name	Minimum	Maximum	Direction	Sequence	Length	%GC	Hairpin Tm	Self Dimer Tm	Tm
**F1**	106	124	forward	AGTTCCTGCCRCCCRTCGA	19	64.7	41.7	None	60.9 - 66.4
**F2**	135	151	forward	ATCGAYGCMCTCATGGC	17	60	61.5	None	54.6 - 59.5
**F3**	177	193	forward	ATCGGCATCRTCGACGG	17	62.5	44	35.1	56.8 - 59.6
**F4**	195	211	forward	AAGTTCCTSCARAGCTT	17	43.8	40.8	17.4	50.1 - 52.8
**F5**	217	235	forward	TCTCCCCCAAGGAGATCCT	19	57.9	63.2	7.4	58.6
**F6**	264	280	forward	TACGGCTCSTCCAGCAT	17	58.8	52	3.5	57.1 - 57.9
**R1**	366	382	reverse	ACCTCGTCGTCRTCCTC	17	62.5	38.3	1.3	55.1 - 58.0
**R2**	422	440	reverse	GATGTTGACCATGGGTTCG	19	52.6	35.3	18.1	56.3
**R3**	525	544	reverse	GTGCGCTGSGGGAAGTACAG	20	65	35.4	24.6	63.1 - 64.0
**R4**	558	575	reverse	GCCCTTCASCTSGTGCAG	18	66.7	42.7	22	60.7 - 61.1

Primer set	Amplicon size	Primer set	Amplicon size	Primer set	Amplicon size	Primer set	Amplicon size	Primer set	Amplicon size	Primer set	Amplicon size
F1R1	277bp	F2R1	248bp	F3R1	206bp	F4R1	188bp	F5R1	166bp	F6R1	119bp
F1R2	335bp	F2R2	306bp	F3R2	264bp	F4R2	246bp	F5R2	224bp	F6R2	177bp
F1R3	439bp	F2R3	410bp	F3R3	368bp	F4R3	350bp	F5R3	328bp	F6R3	281bp
F1R4	470bp	F2R4	441bp	F3R4	399bp	F4R4	381bp	F5R4	359bp	F6R4	312bp

● Too short amplicon size ○ Primer set used for PCR screening

Figure S57. Conserved region for TfuA enzyme PCR primer designing.

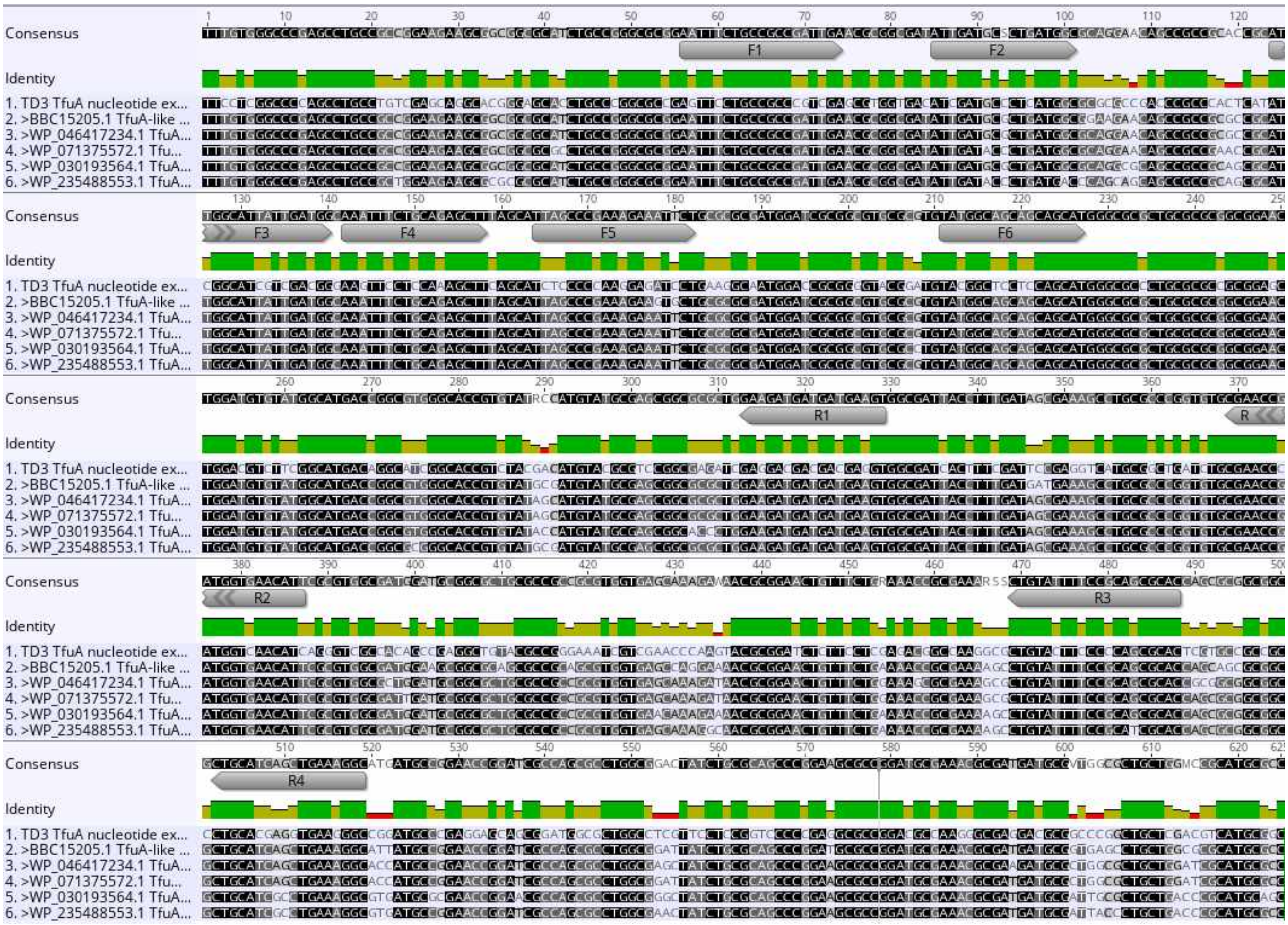

Biosynthetic gene cluster (BGC) analysis

Table S13. antiSMASH analysis result of *Streptomyces* sp. GC2 genome

Region	Type	From	To	Most similar known cluster	Similarity confidence
Region 1	terpene-precursor	93,560	114,579		
Region 2	butyrolactone	116,027	126,983		
Region 3	polyyne,betalactone	172,266	205,707	cepacin A	Low
Region 4	T2PKS,phosphonate	208,628	287,664	homopiloquinone/piloquinone	Medium
Region 5	T1PKS,NRPS	396,469	505,205	galtamycin C/galtamycin D	Low
Region 6	lanthipeptide-class-iii,RiPP-like	649,972	675,754	informatipeptin	Medium
Region 7	lanthipeptide-class-i,NRPS,lanthipeptide-class-ii	776,686	841,269		
Region 8	T1PKS,NRPS-like	1,017,149	1,065,106	cinnabaramide A	Low
Region 9	NRPS,RiPP-like	1,134,340	1,176,592		
Region 10	terpene	1,219,833	1,246,544	hopene	High
Region 11	NRPS,phosphonate,NRPS-like,betalactone,T1PKS	1,329,792	1,440,535		
Region 12	NI-siderophore,RRE-containing,NRPS,T1PKS	1,665,499	1,751,071		
Region 13	terpene	1,867,356	1,889,518	geosmin	High
Region 14	RiPP-like	1,906,731	1,918,056		
Region 15	PKS-like,T1PKS,azoxy-crosslink	1,970,882	2,029,104		
Region 16	phosphonate	2,095,812	2,117,792	rhizocticin A	Low
Region 17	T1PKS,NI-siderophore,NRPS	2,182,921	2,263,864	kinamycin	Low
Region 18	terpene-precursor	2,853,916	2,875,052		
Region 19	terpene	2,876,888	2,897,928	julichrome Q3-3/julichrome Q3-5	Low
Region 20	NRPS	3,029,546	3,089,943		
**Region 21**	**lanthipeptide-class-v,thioamitides**	**3,579,831**	**3,633,015**	**thioholgamide A/thioholgamide B**	**High**
Region 22	T1PKS,T2PKS	4,479,958	4,553,479	spore pigment	High
Region 23	triceptide	4,652,137	4,673,520		
Region 24	lanthipeptide-class-iii	4,697,538	4,735,951		
Region 25	NI-siderophore	5,211,113	5,240,882	desferrioxamin B/desferrioxamine E	High
Region 26	melanin	5,296,759	5,307,328	melanin	Medium
Region 27	lanthipeptide-class-i	5,324,533	5,349,531		
Region 28	T1PKS	5,765,501	5,808,608		
Region 29	ectoine	6,297,884	6,308,294	ectoine	High

Table S14. antiSMASH analysis result of *Streptomyces* sp. TD3 genome

Region	Type	From	To	Most similar known cluster	Similarity confidence
Region 1	T2PKS,terpene	42,935	115,462	spore pigment	High
Region 2	melanin	137,669	148,142	melanin	High
Region 3	terpene-precursor	283,170	323,034		
Region 4	NRPS-like,NRPS,T1PKS,RiPP-like	392,250	478,055	guadinomine/guadinomine B/guadinomic acid	Low
Region 5	ectoine	491,805	502,203	ectoine	High
Region 6	T1PKS,NRPS-like	646,575	698,826		
Region 7	terpene	829,218	850,270	steffimycin D	Low
Region 8	lanthipeptide-class-i	1,050,739	1,077,187		
Region 9	ectoine	1,413,979	1,424,377	ectoine	High
Region 10	terpene	1,894,139	1,915,170		
Region 11	terpene	2,078,977	2,101,127	pentalenolactone	Medium
Region 12	lanthipeptide-class-ii,lanthipeptide-class-iii	2,423,579	2,454,723		
**Region 13**	**lanthipeptide-class-v, thioamides**	**3,172,144**	**3,226,716**	**neothioviridamide**	**High**
Region 14	PKS-like	3,248,769	3,289,788	rustmicin	Low
Region 15	butyrolactone	3,382,222	3,393,133		
Region 16	terpene-precursor	4,739,781	4,760,830		
Region 17	NRPS,T1PKS	4,912,348	4,963,095	BD-12	Low
Region 18	RiPP-like	5,026,032	5,036,319		
Region 19	terpene	5,186,929	5,208,002		
Region 20	NI-siderophore	5,264,342	5,296,944	kinamycin	Low
Region 21	azoxy-crosslink,T1PKS,butyrolactone	5,391,938	5,444,396		
Region 22	NRPS,phosphonate,terpene	5,640,033	5,771,020	xiamycin A	Medium
Region 23	RiPP-like	5,851,857	5,863,146	14-hydroxyisochainin	Low
Region 24	T1PKS,NRPS-like	6,025,855	6,230,845	quinolidomicin A	Low
Region 25	terpene	6,637,339	6,663,897	hopene	Medium
Region 26	T1PKS,NRPS,NRP-metallophore,NRPS-like	6,711,147	6,878,692	venturicidin D/venturicidin E/venturicidin F/venturicidin A	High
Region 27	transAT-PKS,NRPS,NRPS-like,RiPP-like	6,892,259	6,997,027	inthomycin B	High
Region 28	NRPS	7,098,531	7,148,520	mirubactin	Medium
Region 29	terpene	7,149,434	7,177,693	isorenieratene	High

Table S15. Thiogochangamide biosynthetic gene cluster from *Streptomyces* sp. GC2.

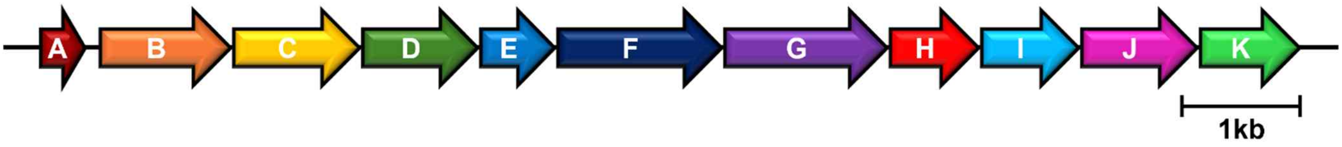

	Gene	Location
**A**	precursor peptide	
**B**	hypothetical protein (biosynthetic (rule-based-clusters) lanthipeptide-class-v: APH)	3,599,831 - 3,600,931, (total: 1101 nt)
**C**	hypothetical protein (biosynthetic (rule-based-clusters) lanthipeptide-class-v: HopA1)	3,600,928 - 3,601,932, (total: 1005 nt)
**D**	hypothetical protein (biosynthetic (rule-based-clusters) lanthipeptide-class-v: APH)	3,601,929 - 3,602,876, (total: 948 nt)
**E**	putative flavoprotein decarboxylase	3,602,873 - 3,603,478, (total: 606 nt)
**F**	putative methyltransferase	3,603,486 - 3,604,703, (total: 1218 nt)
**G**	YcaO-like protein	3,604,741 - 3,606,093, (total: 1353 nt)
**H**	TfuA-like core domain-containing protein	3,606,108 - 3,606,800, (total: 693 nt)
**I**	putative phytanoyl-CoA dioxygenase family protein	3,606,797 - 3,607,669, (total: 873 nt)
**J**	putative protease	3,607,666 - 3,608,499, (total: 834 nt)
**K**	hypothetical protein	3,608,641 - 3,609,483, (total: 843 nt)

Figure S58. Proposed biosynthetic pathways of thiogochangamide A (**1**) and B (**2**)

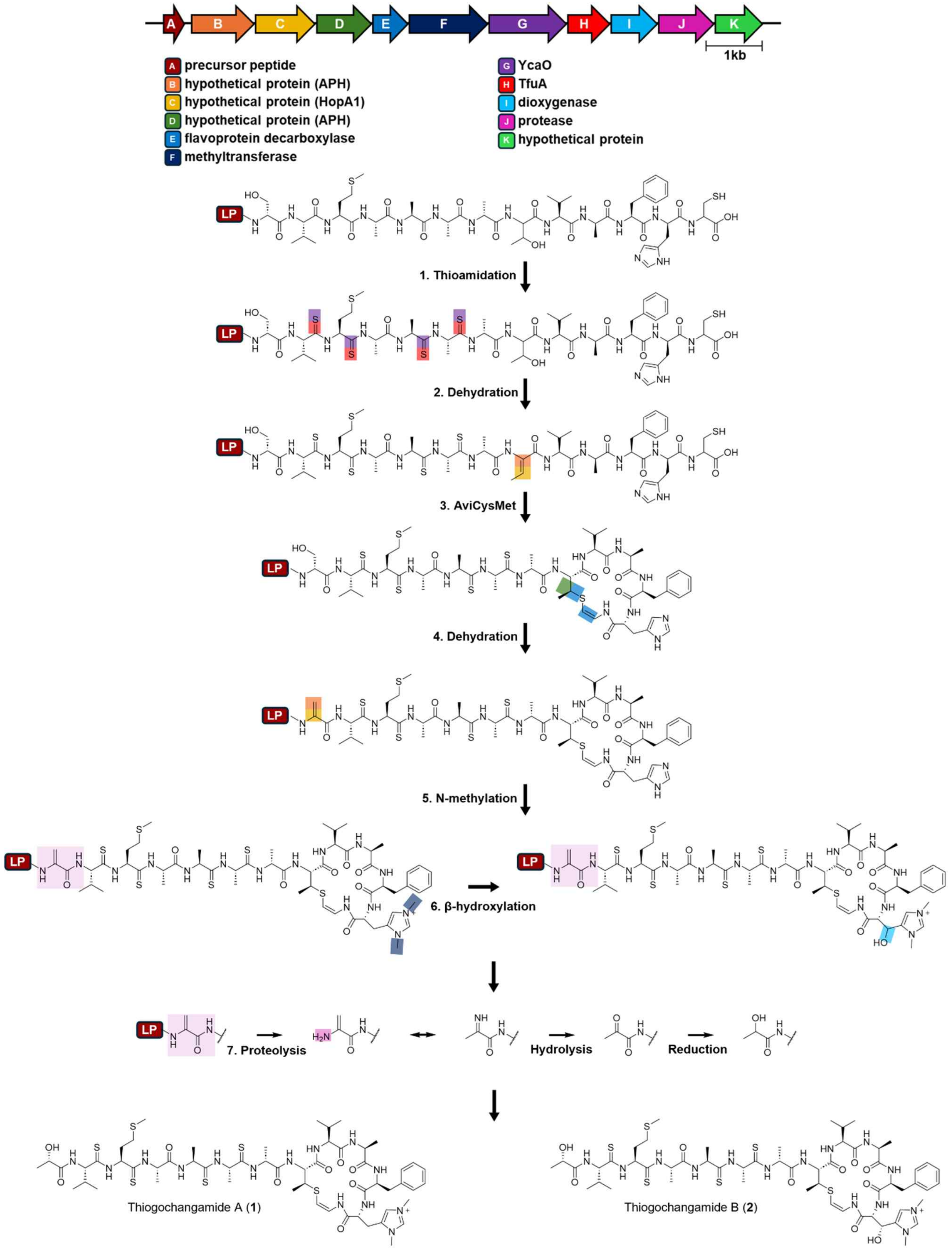

Additional bioassays

Table S16. Anti-proliferative activity of thiogochangamides A and B in a panel of human cancer cell lines.

IC_{50} (μM)	SNU638	SK-Hep-1	A549	HCT116	MDA-MB-231
thiogochangamide A	<0.4	1.53 ± 0.04	<0.4	<0.4	2.37 ± 0.14
thiogochangamide B	<0.4	1.34 ± 0.12	<0.4	<0.4	1.78 ± 0.25
Etoposide	0.17 ± 0.02	0.46 ± 0.02	0.19 ± 0.01	0.9 ± 0.06	4.84 ± 0.32

[a]Cancer cell lines: SNU638 (stomach), SK-HEP-1 (liver), A549 (lung), HCT116 (colon), MDA-MB-231 (breast)
[b]Etoposide was used as a positive control.

Figure S59. Sulforhodamine B (SRB) staining-based cytotoxicity assay in pancreatic cancer cells. PANC-1 and PANC-GR cells were treated with indicated concentrations of thiogochangamide B or gemcitabine for 72 h. Data are presented as the means ± SD from three independent experiments. Statistical significance was determined using one-way analysis of variance (ANOVA) comparing between treated and DMSO control. Statistical significance (***p < 0.001) was assessed using Student's t-test or one-way analysis of variance coupled with Dunnett's t-test.

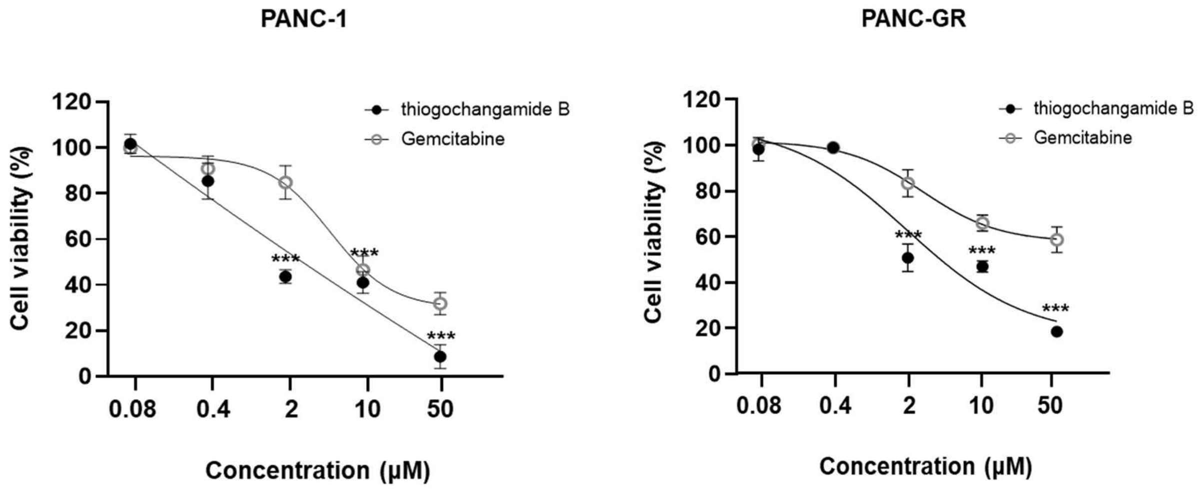

Figure S60. Correlation between CTNNB1 expression and overall survival in pancreatic adenocarcinoma and the effects of periplocin on gemcitabine-resistant pancreatic cancer. (a) CTNNB1 expression levels and overall survival in patients with PC analyzed by Kaplan–Meier method. (b) β-catenin expression levels in PANC-1 and PANC-GR cell lines analyzed with Western blot analysis. β-Actin was used as an internal control. Data are expressed as the means ± standard deviation (SD) of triplicates for each concentration.

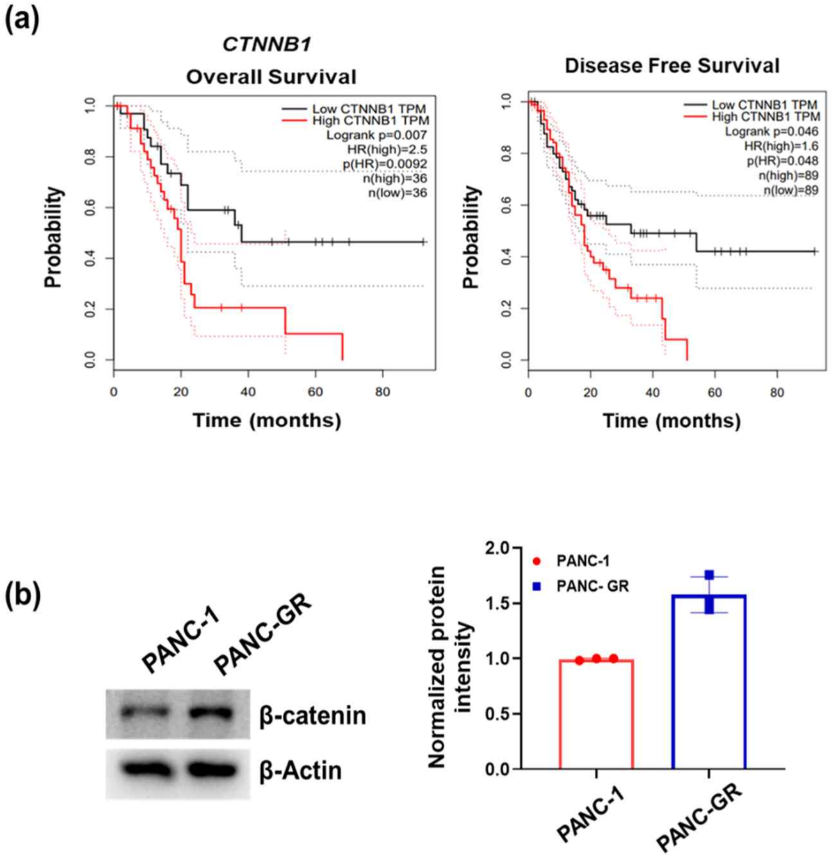

Figure S61. CETSA-based determination of thiogochangamide B-β-catenin protein binding. (a) The CETSA assay was performed using cell lysates, which were either treated with thiogochangamide B or remain untreated. They were heated at various temperatures, and protein samples were collected and analyzed using Western blotting. All data are representative of the results of at least three independent experiments. The relative intensity of β-catenin was quantified using NIH ImageJ 1.52a software.

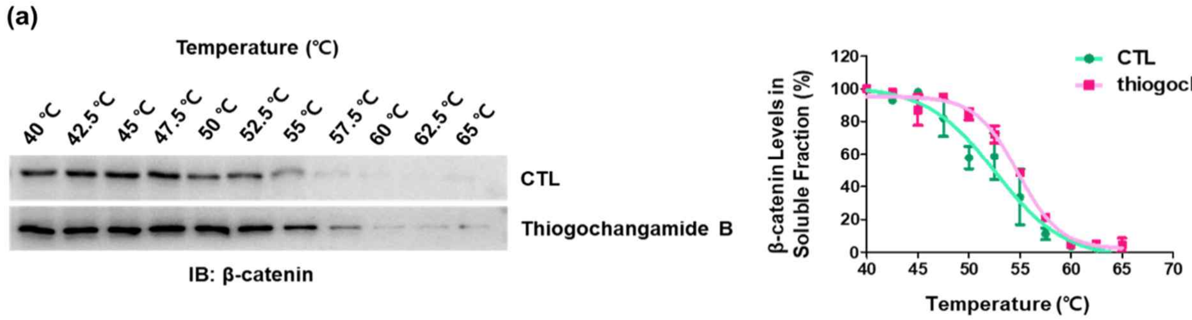

Figure S62. Experimental validation of the inhibitory effect of β-catenin knockdown on cell proliferation. (a) PANC-GR cells were transfected with scrambled siRNA or β-catenin siRNA for 24 h and seeded for an additional 24 h. The effects of β-catenin siRNA on β-catenin protein expression level was evaluated by western blotting analysis. β-Actin was used as an internal control. (b) The effects of β-catenin siRNA on the antiproliferative activity of gemcitabine in PANC-GR cells were evaluated by the sulforhodamine B (SRB) assay after 72 h of gemcitabine treatment. All data are representative of the results of at least three independent experiments. *** $p < 0.001$ statistically significant difference compared with the scrambled siRNA-transfected control.

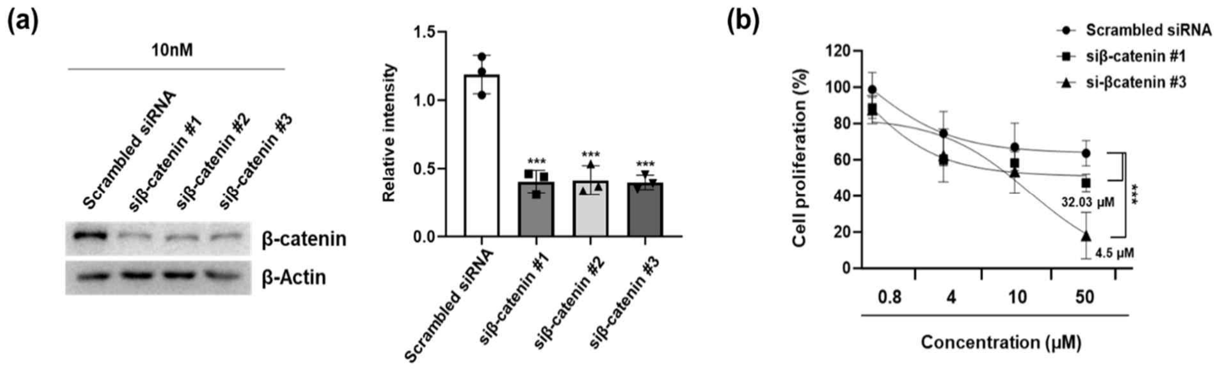

Figure S63. Effects of thiogochangamide B on cell cycle distribution in PANC-GR cells. (a) The cells were treated with represented thiogochangamide B concentrations for 24 h, then collected, and incubated with RNase A and PI. Cell populations were analyzed using flow cytometry. (b) PANC-GR cells were treated with the indicated thiogochangamide B concentrations for 24 h, and CDK4, CDK6, Cyclin D1, Cyclin E, and p21 expression levels were determined by western blot analysis. β-Actin was used as internal control. Data are presented as the means ± SD from three independent experiments.

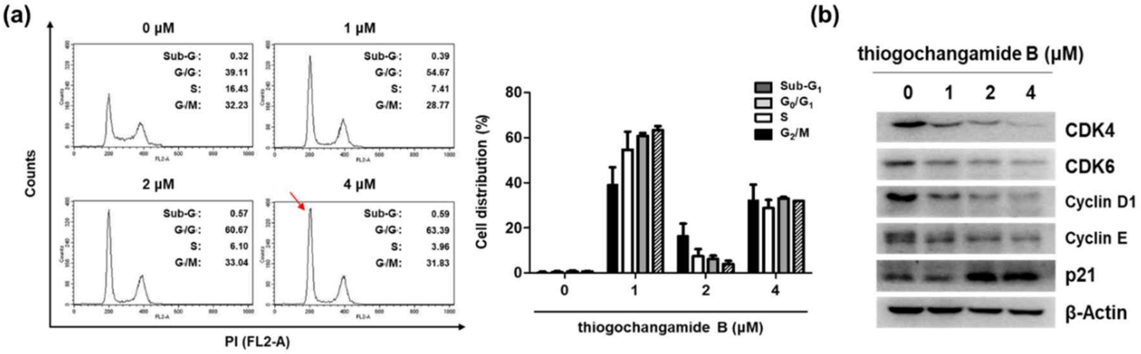

Figure S64. Induction of cell death by thiogochangamide B treatment. (a) Cells were treated with the indicated thiogochangamide B concentrations for 48 h, and stained with Annexin V-fluorescein isothiocyanate and PI. Apoptotic populations, annexin V/PI-positive cells, were analyzed by flow cytometry. Each cell population and the total cell death (early apoptosis + late apoptosis + necrosis) quantified and displayed as % of the control (bottom panel). (b) Cells were treated with the indicated thiogochangamide B concentrations for 48 h, and the expression levels of cleaved caspse-8 and cleaved caspase-9 analyzed by western blotting. β-Actin was used internal control. Data are expressed as the means ± SD from triplicates experiments.

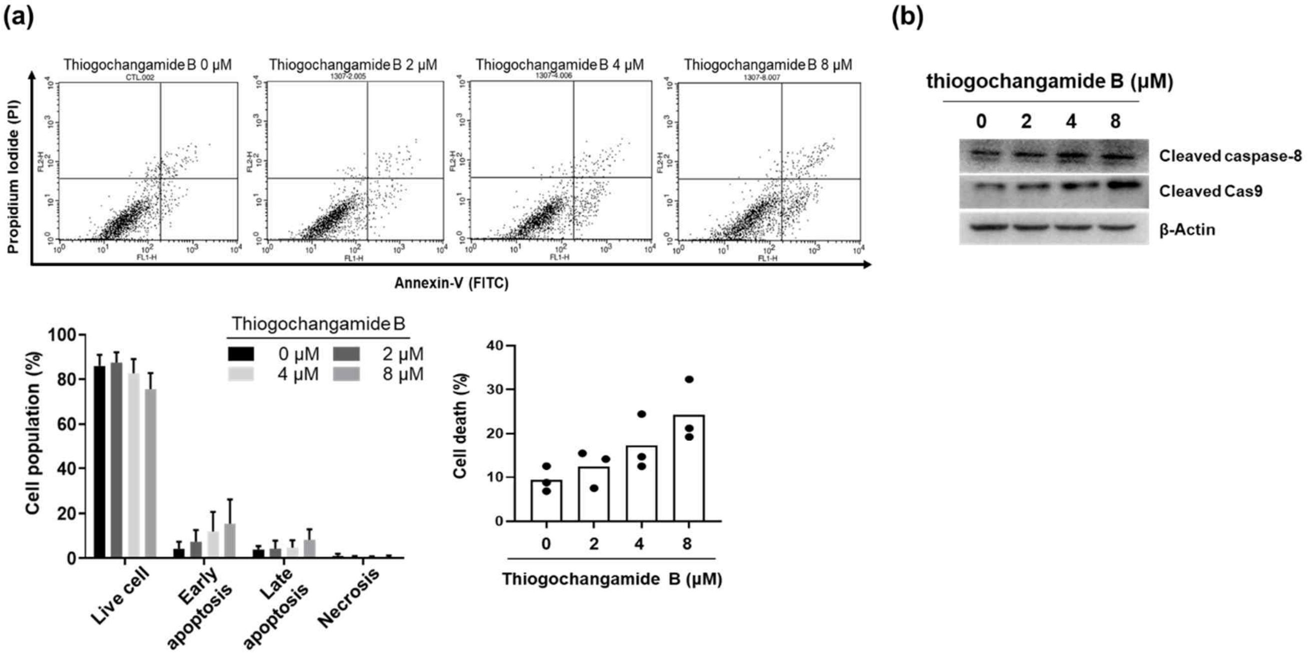

Figure S65. Effects of β-catenin knockdown on cell migration, invasion and EMT marker in pancreatic cancer cells. (a) Wound healing assay was performed to evaluate the migratory ability of pancreatic cancer cells after β-catenin knockdown. Cells were transfected with scrambled siRNA or β-catenin siRNA (siβ-cat), and wound closure was monitored at 0 and 24 hours. Quantification of wound closure area (%) shows a significant reduction in migration following β-catenin knockdown (right panel). Data represent mean ± SD from three independent experiments. ****p* < 0.01. (b) Transwell invasion assay further confirmed the impaired migratory capacity of cells with β-catenin knockdown. Cells were stained with crystal violet after 24 h of incubation. (c) Western blot analysis of epithelial-mesenchymal transition (EMT) markers. Knockdown of β-catenin led to increased expression of E-cadherin (epithelial marker) and decreased expression of Snail (mesenchymal markers), indicating suppression of EMT.

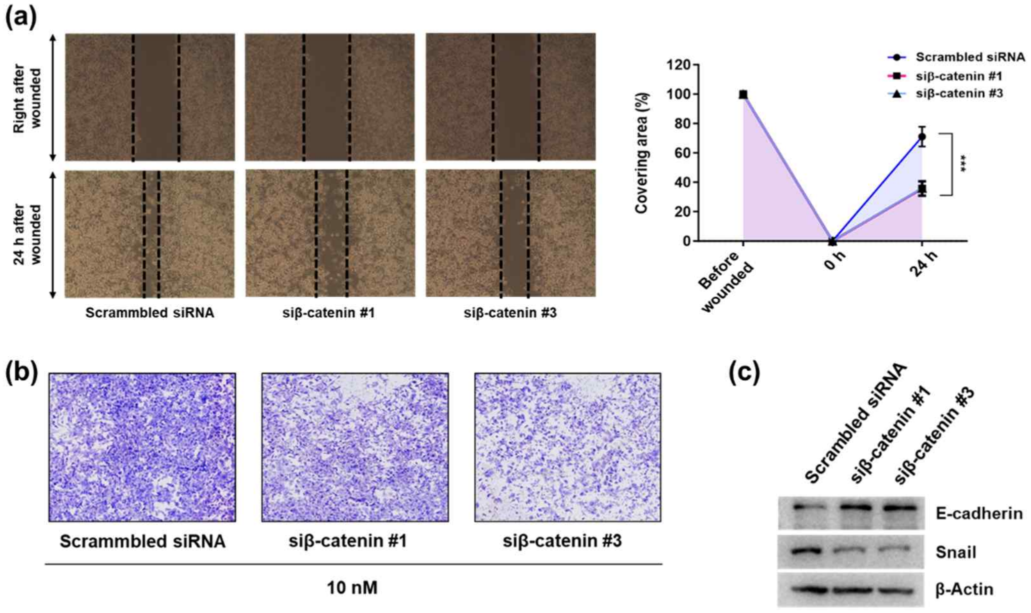

Figure S66. The changes of mouse body weight. The body weights of mice were measured every 3 days to monitor tumor growth and general toxicity.

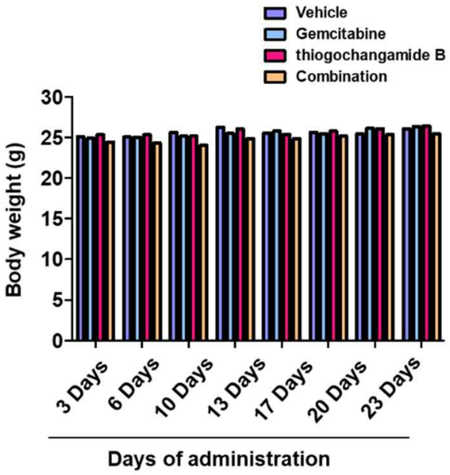

Figure S67. Calculation of the synergistic anti-tumor effect of thiogochangamide B in combination with gemcitabine in a gemcitabine-resistant pancreatic cancer xenograft model. A combination ratio greater than 1 indicates a synergistic effect, whereas a ratio less than 1 suggests an antagonistic or less-than-additive effect**.**

.

Combination effect of in vivo	
FTV of gemcitabine	0.76
FTV of thiogochangamide B	0.48
FTV of combination	0.35
CI value	**1.03**

Isothermal titration calorimetry (ITC) assay

Figure S68. Isothermal titration calorimetry (ITC) assay using recombinant β-catenin and thiogochangamide B. (a) Coomasie-stained 12% SDS-PAGE gel showing the purity of the recombinant β-catenin used for ITC experiment. (b) ITC thermogram (top) and integrated binding enthalpy (bottom). Titrations were performed at 25°C

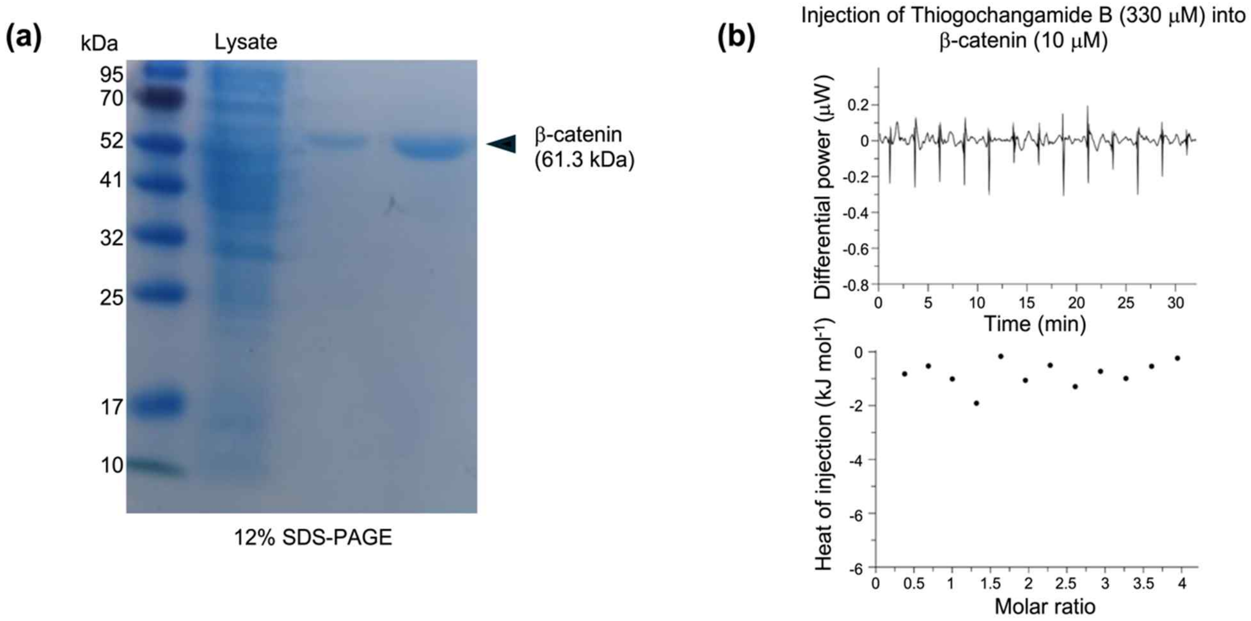

In vitro metabolic stability

Figure S69. *In vitro* metabolic and proteolytic stability of thiogochangamide B. (A) Metabolic stability in Balb/c mouse liver S9 fractions. The left panel shows the percentage of compound remaining over time, and the right panel displays the natural logarithm (Ln) of the percentage remaining used to calculate intrinsic clearance ($CL_{int,\ in\ vitro}$) and half-life ($t_{1/2}$). The compound was incubated with (red circles) or without (blue triangles) an NADPH-generating system. The solid lines in the right panel represent the linear regression fit. (B) Proteolytic stability in Balb/c mouse plasma. The compound was incubated in plasma at 37°C for 120 min. Data are presented as mean ± SD (n=4).

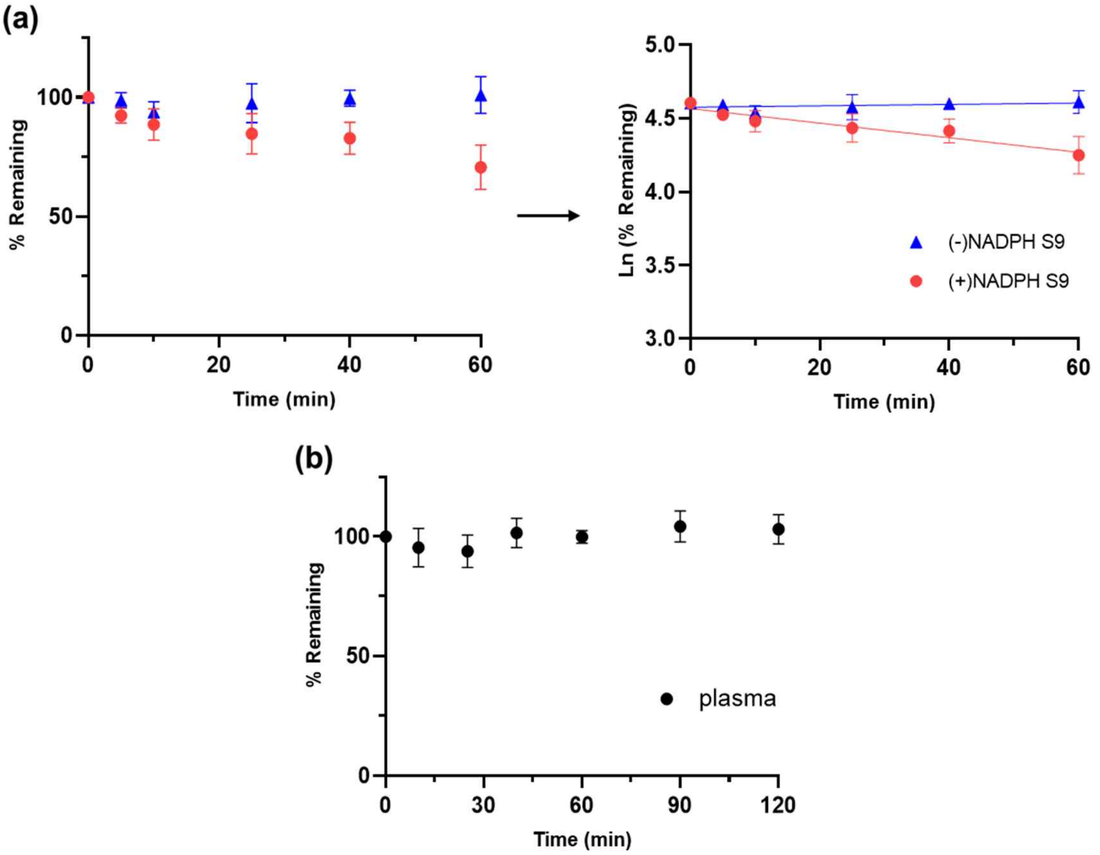

To provide a tentative *in vivo* context, the liver S9 clearance data were extrapolated using reported mouse physiological scaling parameters (S9 content = 143 mg protein/g liver, liver weight = 87.5 g/kg, and hepatic blood flow Qh = 90 mL/min/kg). Under the assumption of $f_{u,inc}$ = 1 (*i.e.*, no correction for incubation binding), the scaled intrinsic clearance was estimated to be approximately 62 mL/min/kg[9–11]. The hepatic extraction ratio (EH) was then predicted using the well-stirred model, assuming $f_{u,blood}$ = 1, which represents a conservative upper-bound scenario with respect to blood binding. Even under these assumptions, the predicted extraction ratio was approximately 0.41. This value suggests that thiogochangamide B is unlikely to behave as a high-extraction compound, and that the true hepatic extraction is expected to be lower once protein binding is accounted for[11]. Taken together with its high plasma stability, these results indicate a favorable metabolic stability profile and support thiogochangamide B as a promising lead compound for further pharmacokinetic and *in vivo* evaluation.

References

1. I. Bonnard, I. Manzanares, and K. L. Rinehart, "Stereochemistry of Kahalalide F," *Journal of Natural Products* 66 (2003): 1466–1470, https://doi.org/10.1021/np030334c
2. K. Bahrami, M. M. Khodaei, and Y. Tirandaz, "Desulfurization of Thioamides into Amides with $H_2O_2/ZrCl_4$ Reagent System," *Synthesis* 3 (2009): 369–372, https://doi.org/10.1055/s-0028-1083314
3. S. G. Smith and J. M. Goodman, "Assigning Stereochemistry to Single Diastereoisomers by GIAO NMR Calculation: The DP4 Probability," *Journal of the American Chemical Society* 132 (2010): 12946–12959, https://doi.org/10.1021/ja105035r
4. S. G. Smith and J. M. Goodman, "Assigning the Stereochemistry of Pairs of Diastereoisomers Using GIAO NMR Shift Calculation," *Journal of Organic Chemistry* 74 (2009): 4597–4607, https://doi.org/10.1021/jo900408d
5. E. S. Bae, W. S. Byun, C. W. Ock, W. K. Kim, H. J. Park and S. K. Lee, "Periplocin Exerts Antitumor Activity by Regulating Nrf2-Mediated Signaling Pathway in Gemcitabine-Resistant Pancreatic Cancer Cells," *Biomedicine & Pharmacotherapy* 157 (2023): 114039, https://doi.org/10.1016/j.biopha.2022.114039
6. W. K. Kim, D. H. Bach, H. W. Ryu, et al., "Cytotoxic Activities of *Telectadium Dongnaiense* and Its Constituents by Inhibition of the Wnt/β-Catenin Signaling Pathway," *Phytomedicine* 34 (2017): 136–142, https://doi.org/10.1016/j.phymed.2017.08.008
7. E. S. Bae, J. Hong, Y. Lim, et al., "Evo312: An Evodiamine Analog and Novel PKCβI Inhibitor with Potent Antitumor Activity in Gemcitabine-Resistant Pancreatic Cancer," *Journal of Medicinal Chemistry* 67 (2024): 14885–14911, https://doi.org/10.1021/acs.jmedchem.4c00213
8. Y. Chen, N. Guggisberg, M. Jorda, et al., "Combined Src and Aromatase Inhibition Impairs Human Breast Cancer Growth in vivo and Bypass Pathways are Activated in AZD0530-Resistant Tumors," *Clinical Cancer Research* 15 (2009): 3396–3405, https://doi.org/10.1158/1078-0432.CCR-08-3127
9. B. Davies and T. Morris, "Physiological Parameters in Laboratory Animals and Humans," *Pharmaceutical Research* 10 (1993): 1093-1095, https://doi.org/10.1023/A:1018943613122
10. A. Punt, A. Paini, M. G. Boersma, *et al*., "Use of Physiologically Based Biokinetic (PBBK) Modeling to Study Estragole Bioactivation and Detoxification in Humans as Compared with Male Rats," *Toxicological Sciences* 110 (2009): 255-269, https://doi.org/10.1093/toxsci/kfp102
11. J. B. Houston, "Utility of in vitro Drug Metabolism Data in Predicting in vivo Metabolic Clearance," *Biochemical Pharmacology* 47 (1994): 1469-1479, https://doi.org/10.1016/0006-2952(94)90520-7
